# Examining the Relationship between Aptamer Complexity and Molecular Discrimination of a Low-Epitope Target

**DOI:** 10.1021/acscentsci.4c01377

**Published:** 2024-11-11

**Authors:** Linlin Wang, Juan Canoura, Caleb Byrd, Thinh Nguyen, Obtin Alkhamis, Phuong Ly, Yi Xiao

**Affiliations:** Department of Chemistry, North Carolina State University, 2620 Yarbrough Dr., Raleigh, North Carolina 27695, United States

## Abstract

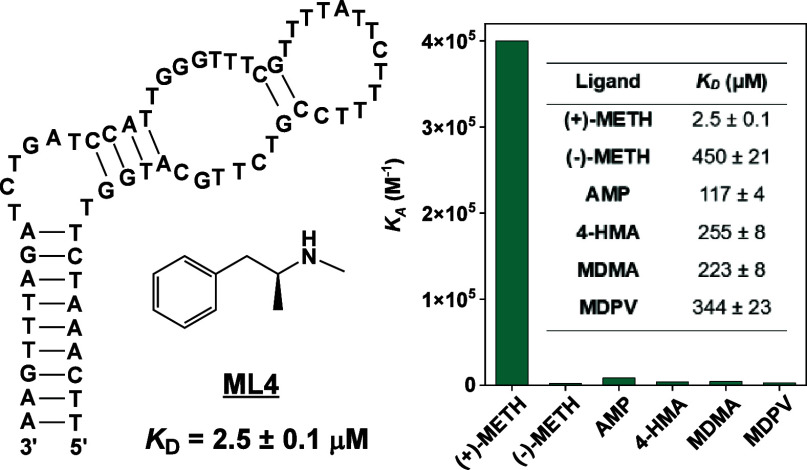

Aptamers are oligonucleotide-based affinity reagents that are increasingly being used in various applications. Systematic evolution of ligands by exponential enrichment (SELEX) has been widely used to isolate aptamers for small-molecule targets, but it remains challenging to generate aptamers with high affinity and specificity for targets with few functional groups. To address this challenge, we have systematically evaluated strategies for optimizing the isolation of aptamers for (+)-methamphetamine, a target for which previously reported aptamers have weak or no binding affinity. We perform four trials of library-immobilized SELEX against (+)-methamphetamine and demonstrate that N30 libraries do not yield high-quality aptamers. However, by using a more complex N40 library design, stringent counter-SELEX, and fine-tuned selection conditions, we identify aptamers with high affinity for (+)-methamphetamine and better selectivity relative to existing antibodies. Bioinformatic analysis from our selections reveals that high-quality aptamers contain long conserved motifs and are more informationally dense. Finally, we demonstrate that our best aptamer can rapidly detect (+)-methamphetamine at toxicologically relevant concentrations in saliva in a colorimetric dye-displacement assay. The insights provided here demonstrate the challenges in generating high-quality aptamers for low complexity small-molecule targets and will help guide the design of more efficient future selection efforts.

## Abstract 2

We here provide an account of the isolation of high-quality DNA aptamers with high specificity for the low-complexity small-molecule drug (+)-methamphetamine—a challenging target for which previously reported aptamers have exhibited weak or no binding affinity.

## Introduction

Aptamers are oligonucleotides that recognize specific molecules with high affinity.^1,2^ They have several favorable attributes relative to conventional protein-based receptors like antibodies, such as low cost, ease of synthesis, straightforward sequence engineering, high stability, reversible denaturation, and the capability to tune their binding properties.^3,4^ Aptamers are increasingly being utilized as bioreceptors in sensors for medical diagnostics, health monitoring, food safety, and forensics.^5^ Given their simplicity and ease of use, aptamer-based assays have unique advantages over methods such as mass spectrometry in terms of turnaround time and cost. Moreover, their distinctive advantages are enabling unprecedented biosensing applications that are beyond the reach of antibodies, such as real-time molecular detection in live cells,^6^ tissues,^7^ organs,^8^ and blood circulation.^9^ Aptamers are isolated from randomized libraries through an *in vitro* method known as systematic evolution of ligands by exponential enrichment (SELEX).^1,2^ Here, the library is incubated with the target, after which aptamers are separated from binding-incompetent sequences and amplified. The enriched pool of sequences resulting from this process is subjected to another cycle of selection until the pool exhibits satisfactory binding properties.

While there have been numerous successes in the generation of aptamers for protein targets, the isolation of aptamers for small molecules remains challenging. Historically, these selections have been impeded by the need to covalently attach the small-molecule target to a solid support to facilitate isolation of target-binding sequences^1,10^ Such conjugation is chemically challenging, especially for targets that lack functional groups amenable to covalent linkage, and also masks the very few structural elements that aptamers could bind to, resulting in aptamers with low affinity and specificity. Nutiu and Li effectively addressed these limitations by immobilizing the library—rather than the target—onto microbeads that have been coupled to complementary DNA (cDNA) strands that hybridize to a portion of the library sequence.^11^ When challenged with target, the target binders dissociate from the cDNA, facilitating their separation from the beads and binding-incompetent sequences. With further refinements and simplification by the Stojanovic group,^12^ this library-immobilized SELEX method now represents the most reliable means of identifying aptamers for small-molecule targets. For instance, we and others have utilized this selection strategy to isolate high-affinity DNA aptamers with good specificity for tetrahydrocannabinol^13^ and theophylline,^14^ and RNA aptamers for paromomycin^15^ as well as guanine and quinine.^16^ Nevertheless, the selection of aptamers for targets that have very few distinct epitopes still requires careful experimental design, trial and error, and optimization. With only two successes reported thus far in the form of DNA aptamers,^17,18^ there is still a lack of insight into how to reliably isolate high-quality aptamers for such challenging targets. For example, while isolating aptamers for γ-amino butyric acid, a molecule with just an amino and a carboxyl moiety, the Stojanovic group encountered multiple failures using an N36 library and only finally achieved success with an N44 library.^19^ A detailed account and examination of such selection trials and parameters (e.g., length of the random region) would be very valuable in terms of enabling the design of successful selection experiments for other problematic targets.

To address this knowledge gap and examine how selection conditions influence the aptamer isolation process, we have performed a series of independent SELEX experiments to isolate aptamers for the small-molecule drug (+)-methamphetamine. Our reasoning for choosing this target was 3-fold. First, aptamers that bind (+)-methamphetamine would be of high value given the prevalence of (+)-methamphetamine abuse and its impact on public health,^20,21^ and hence the need for sensors that can detect this drug in seized substances and biological samples. Second, (+)-methamphetamine is a very low-complexity target—and thus a formidable challenge for selecting aptamers—with essentially two functional groups available for recognition by oligonucleotides: a phenyl group and an amine. Finally, there are at least three different published studies from the past decade describing efforts to isolate aptamers that bind to methamphetamine,^22−24^ giving us the opportunity to systematically study the impact of different selection strategies and conditions on aptamer quality. In this work, we first determined that previously reported methamphetamine aptamers either have weak or no affinity for this target. We then performed our own selection experiments—four trials in total—facing numerous barriers throughout this process. Throughout these trials, we determined that while it is possible for aptamers to bind (+)-methamphetamine, the capability to discriminate this target from structurally similar molecules necessitated more complex aptamers with larger binding domains. Eventually, we identified new aptamers with excellent specificity for (+)-methamphetamine, with 50-fold and 89-fold greater affinity for this target relative to amphetamine and MDMA, respectively, surpassing the capabilities of existing antibodies. We believe this account will provide valuable insights into how best to execute selections for low-complexity targets in the future.

## Experimental Section

### Reagents and Materials

Phosphate buffered saline (10×, molecular biology grade) and molecular biology grade water were purchased from Corning. Magnesium chloride solution for molecular biology was purchased from Sigma-Aldrich. Procaine hydrochloride, l-tyrosine, l-phenylalanine, quinine hemisulfate salt monohydrate, caffeine, diphenhydramine HCl, cocaine HCl, sodium dodecyl sulfate, serotonin HCl, tyramine, 3,4-dihydroxyphenylacetic acid and dopamine HCl were purchased from Sigma-Aldrich. Lidocaine hydrochloride monohydrate was purchased from Alfa Aesar. Morphine sulfate hydrate, fentanyl HCl, alprazolam, (+)-methamphetamine HCl, (−)-methamphetamine HCl, amphetamine HCl, bupropion HCl, 3,4-methylenedioxypyrovalerone (MDPV) HCl, 3,4-methylenedioxymethamphetamine (MDMA) HCl, methylphenidate HCl, methadone HCl, 4-hydroxymethamphetamine HCl, 4-hydroxyamphetamine HCl, homovanillic acid, (+)-pseudoephedrine HCl, epinephrine HCl, and norepinephrine bitartrate hydrate were purchased from Cayman Chemicals. SYBR Gold and streptavidin-coated agarose resin were purchased from Thermo Fisher Scientific. X-732–91B dye was retrieved from the Max Weaver Dye Library at North Carolina State University. Microgravity columns (500 μL) were purchased from Bio-Rad. GoTaq Hot Start Colorless Master Mix was purchased from Promega. Amicon Ultra centrifugal filters (3 kDa MWCO) were purchased from Sigma-Aldrich. The QIAquick PCR purification kit was purchased from Qiagen. EDTA (0.5 M, pH 8.0) and formamide were purchased from Fisher Scientific. Exonuclease I (Exo I, *E. coli*, 20 U/μL) and Exonuclease III (Exo III, *E. coli*, 100 U/μL) and T5 exonuclease (T5 Exo, 10 U/μL) were purchased from New England Biolabs. All other chemicals were purchased from Sigma-Aldrich unless otherwise specified. Ultrapure water (resistivity = 18.2 MΩ•cm, 25 °C) was obtained from a Milli-Q EQ 7000 water purification system.

### Oligonucleotides

All DNA oligonucleotides for SELEX (see Supporting Information (SI), Table S1 for sequences) were purchased from Integrated DNA Technologies. The random library and sequencing primers were PAGE purified, and SELEX PCR primers and complementary DNA (cDNA15-bio) were HPLC purified. All other DNA oligonucleotides were purified by standard desalting. DNA was dissolved in molecular biology grade water, and concentrations were determined using a NanoDrop 2000 Spectrophotometer (Thermo Fisher Scientific).

### SELEX Procedure

Four different trials of library-immobilized SELEX were performed to isolate DNA aptamers that bind to (+)-methamphetamine. The basic procedure follows a previously reported protocol,^12^ and details of the selection process are provided in SI, Tables S2–5. Briefly, the random or enriched library was mixed with a 15-nt biotinylated complementary DNA (cDNA15-bio) at a molar ratio of 1:5 in 250 μL selection buffer, consisting of 1× PBS diluted from 10× PBS (101.4 mM Na_2_HPO_4_, 17.6 mM KH_2_PO_4_, 1369 mM NaCl, 27 mM KCl) and 1 mM MgCl_2_, heated at 95 °C for 10 min, then slowly cooled to room temperature over 20 min to allow the library to hybridize with the cDNA. Meanwhile, a 500 μL microgravity column was filled with 300 μL of molecular biology grade water and subjected to vacuum degassing for 1 min. 250 μL of streptavidin-coated agarose resin was then loaded into the column and washed five times with 250 μL selection buffer. The hybridized library-cDNA complexes were added to the column for immobilization onto the agarose resin. The eluate was collected and flowed through the column three times to maximize library loading onto the agarose resin. The column was then washed with 250 μL selection buffer 10–50 times to remove sequences that weakly bind to the cDNA. Afterward, 250 μL of (+)-methamphetamine dissolved in selection buffer was added to the column, displacing (+)-methamphetamine-binding sequences from the biotinylated cDNA into the eluate. This step was performed three times, and the eluate was combined and then purified using a 3 kDa MWCO filter to remove (+)-methamphetamine and salts and concentrate the solution to <100 μL. The enriched library was then PCR amplified using 600 μL GoTaq Hot Start Colorless Master Mix (2 × ) with 1 μM forward primer (FP) and 1 μM biotinylated reverse primer (RP-bio). PCR was performed using a Bio-Rad C1000 thermal cycler with the following conditions: 2 min at 95 °C; 9–13 cycles of 95 °C for 15 s, 58 °C for 30 s, and 72 °C for 45 s; 5 min at 72 °C. The optimal number of amplification cycles was confirmed by 15% PAGE. Single-stranded DNA was prepared as reported previously,^12^ and then purified and concentrated by a 3 kDa MWCO filter. The concentration was determined by a NanoDrop 2000 spectrophotometer. Counter-SELEX was performed from round 2 in trials 1, 3, and 4 to remove sequences that bound to interferent molecules. After the beginning of selection buffer wash, 250 μL of various counter-targets dissolved in selection buffer were added to the column. Detailed information regarding counter-targets is provided in SI, Tables S2–5. After counter-SELEX, the column was washed with selection buffer 10–40 times to remove any residual counter-targets and nonspecific binders. Positive selection with (+)-methamphetamine was then performed as described above.

### High-Throughput Sequencing (HTS) of Pools and Bioinformatic Analysis

Selection rounds 13 and 19 from trial 1; rounds 9 and 11 from trial 2; rounds 7, 13, and 18 from trial 3; and rounds 9, 10, 11, 13, 14, and 15 from trial 4 were subjected to Illumina-based HTS by Azenta Life Sciences. To prepare SELEX pools for sequencing, 100 nM of each SELEX pool was subjected to PCR amplification using 1 μM of customized forward and reverse primers containing partial Illumina adapters. The PCR conditions employed were as follows: 2 min at 95 °C; 10 cycles of 95 °C for 15 s, 58 °C for 30 s, and 72 °C for 45 s; and 5 min at 72 °C. The PCR products were then cleaned using the QIAqick PCR purification kit, and the successful addition of adapters was confirmed using denaturing polyacrylamide gel electrophoresis (PAGE), after which 25 μL of the 20 ng/μL purified pool was submitted for sequencing. Each pool yielded ∼300,000–1,345,800 reads. Prior to analysis, complementary sequences were converted to their reverse complement using the fastx toolkit and combined with the forward reads. The constant region was then removed using cutadapt,^25^ and sequences containing ‘N’ nucleotides in the random region were discarded. Finally, FASTAptamer^26^ was used to determine the abundance of each unique sequence as well as its enrichment-fold throughout several SELEX rounds. Summary HTS statistics for trials 1–4 is provided in SI, Table S6. Aptamer families were discovered using the Raptgen software.^27^

### Exonuclease Digestion Fluorescence Assay for Aptamer Specificity Screening

The exonuclease digestion fluorescence assay was performed as previously described.^28,29^ Each aptamer (final concentration: 0.5 μM) was diluted in PBS buffer (final concentration: 1 × , pH 7.4) and heated to 95 °C for 10 min, then immediately cooled on ice for 1 min. MgCl_2_ (final concentration: 1 mM), and BSA (final concentration: 0.1 mg/mL) were added immediately. Five μL of this aptamer solution was added to 20 μL of selection buffer, (+)-methamphetamine dissolved in selection buffer (final concentration: 250 or 500 μM), or interferents (procaine, lidocaine, caffeine, quinine, diphenhydramine, amphetamine, cocaine, homovanillic acid, methylphenidate, (+)-pseudoephedrine, alprazolam, epinephrine, bupropion, methadone, morphine, 3,4-Methylenedioxypyrovalerone (MDPV), 3,4-Methylenedioxymethamphetamine (MDMA), fentanyl, dopamine, 4-hydroxymethamphetamine (4-HMA), 4-hydroxyamphetamine (4-HA), phenylalanine, 3,4-dihydroxyphenylacetic acid (DOPAC), norepinephrine, tyrosine, tyramine, or serotonin) dissolved in selection buffer (final concentration: 250 μM, except for alprazolam which was 50 μM and included 5% DMSO in the buffer). The mixture was incubated at 25 °C for 30 min, after which 25 μL of Exo III and Exo I (final concentrations 0.025 U/μL and 0.05 U/μL, respectively) or T5 Exo and Exo I (final concentrations 0.2 U/μL and 0.015 U/μL, respectively) in selection buffer containing 0.1 mg/mL BSA was added to begin the digestion reaction. Five μL of sample was collected at various time-points and added to 30 μL of quenching solution (1× PBS, 1.16× SYBR Gold, 25 mM EDTA,14.6% (v/v) formamide) in the wells of a 384-well black microplate. SYBR Gold fluorescence was recorded using a Tecan Spark plate reader (excitation: 495 nm, emission: 537 nm, bandwidth: 5 nm). Fluorescence was plotted against each time-point to construct time-course digestion plots of each sample. Enzymatic inhibition was measured in terms of the resistance value, which is calculated using the formula (AUC_t_/AUC_0_) – 1, where AUC_t_ and AUC_0_ are the areas under the curve of the time-course data with and without target, respectively. The integration time was customized for each aptamer and was chosen as the point at which fluorescence reached 10% of its initial value. The fluorescence of each sample was recorded 10 times, and average values were used for analysis.

### Isothermal Titration Calorimetry (ITC)

All experiments to determine binding aptamer affinity using ITC were conducted on a Malvern MicroCal PEAQ-ITC, and the data were analyzed with MicroCal PEAQ-ITC Analysis Software using a one-site binding model. Each aptamer was tested at 23 °C with both aptamer and target dissolved in either selection buffer described here or buffers reported previously in the literature. For the determination of methamphetamine affinity, 300 to 5000 μM (+)-methamphetamine HCl was titrated into 20 to 100 μM aptamer during a single titration run. The run consisted of a 60 s equilibration followed by one 0.4-μL injection to purge the syringe, then 19 successive 2-μL injections with either 180 or 150 s spacing. For amphetamine affinity measurements, 500 or 1,500 μM amphetamine HCl was titrated into 20 or 40 μM aptamer. The runs again consisted of a 60 s equilibration with a single 0.4-μL purge injection and 19 times of 2-μL injections, all with 180 s spacing. For some of the aptamers, a double titration of amphetamine was required to reach saturation. This double titration was performed by first running a single titration as described above, but not emptying the cell upon completion. Instead, only the overflow of the sample cell was removed; the syringe was reloaded with target, and a second titration was started with the same parameters as the first. To combine the two experiments, MicroCal ITC software was used. Specific conditions and experimentally determined binding affinity and thermodynamic parameters are presented in SI, Tables S7–8.

### Optimizing Aptamer-Dye Ratio for Detection of (+)-Methamphetamine via Dye-Displacement Assay

All aptamer-based dye-displacement assays were conducted at room temperature. 400 μM dye X-732–91B was prepared in DMSO, and 1 μL was pipetted to the bottom of PCR tubes. Then, 99 μL of aptamer ML4 solution, prepared at a final concentration of 0–10 μM in selection buffer containing 0.01% SDS and 0.001% Triton X-100, was added to each PCR tube to form aptamer-dye complexes. After gentle mixing and centrifugation, 75 μL of the aptamer-dye complex was loaded into the wells of a transparent 384-well plate. Absorbance spectra were recorded at 0, 5, and 10 min from 300–900 nm with 5 nm step size using a Tecan Spark microplate reader. All plots were generated in Origin 2023b. Dye monomers and aggregates were respectively calculated as the area under the curve (AUC) from 505–620 nm and 400–505 nm. The samples were then transferred to a white 384-well plate for photography with a Nikon D750 camera.

### (+)-Methamphetamine Detection in Buffer and Saliva via Dye-Displacement Assay

For detection in buffer, aptamer and dye were mixed in the PCR tube at a final concentration of 4 μM dye and 6 μM aptamer. 50 μL of the aptamer-dye complex was immediately added to PCR tubes containing 50 μL of (+)-methamphetamine or interferents in selection buffer. For the (+)-methamphetamine calibration curve, the final concentration ranged from 0–200 μM. For specificity testing, the interferents were present at 50 μM with target at either 25 or 50 μM. For detection in saliva, pooled saliva was collected from four drug-free, healthy, consenting individuals (three male, one female). The saliva was first centrifuged for 30 min at 20,000 rcf to remove any solid matter, and the supernatant was then filtered using a 0.22-μm filter. Drug-spiked saliva was prepared by adding 5 μL of target at various concentrations into 50 μL of 100% saliva. Then, 45 μL of dye-aptamer complex was added to the spiked saliva and mixed for 10 s. Thus, the saliva is diluted by 50% in 1× buffer. The final concentrations of dye-aptamer complexes and target were the same as for the calibration in buffer. 75 μL of the sample mixture was immediately loaded into the wells of a 384-well plate for absorbance measurements in a Tecan Spark microplate reader using the same settings as for the aptamer optimization. Data analysis was conducted in Origin 2023b, using signal gain as the metric for target detection. Signal gain was calculated as (R–R_0_)/R_0_, where R and R_0_ are the ratio of aggregate (400–505 nm) to monomer (505–620 nm) AUC for samples with and without target/interferent. Samples were photographed after transfer to a white 384-well plate with a Nikon D750 camera.

## Results

### Assessment of Previously Reported Methamphetamine Aptamers

**Figure 1:**
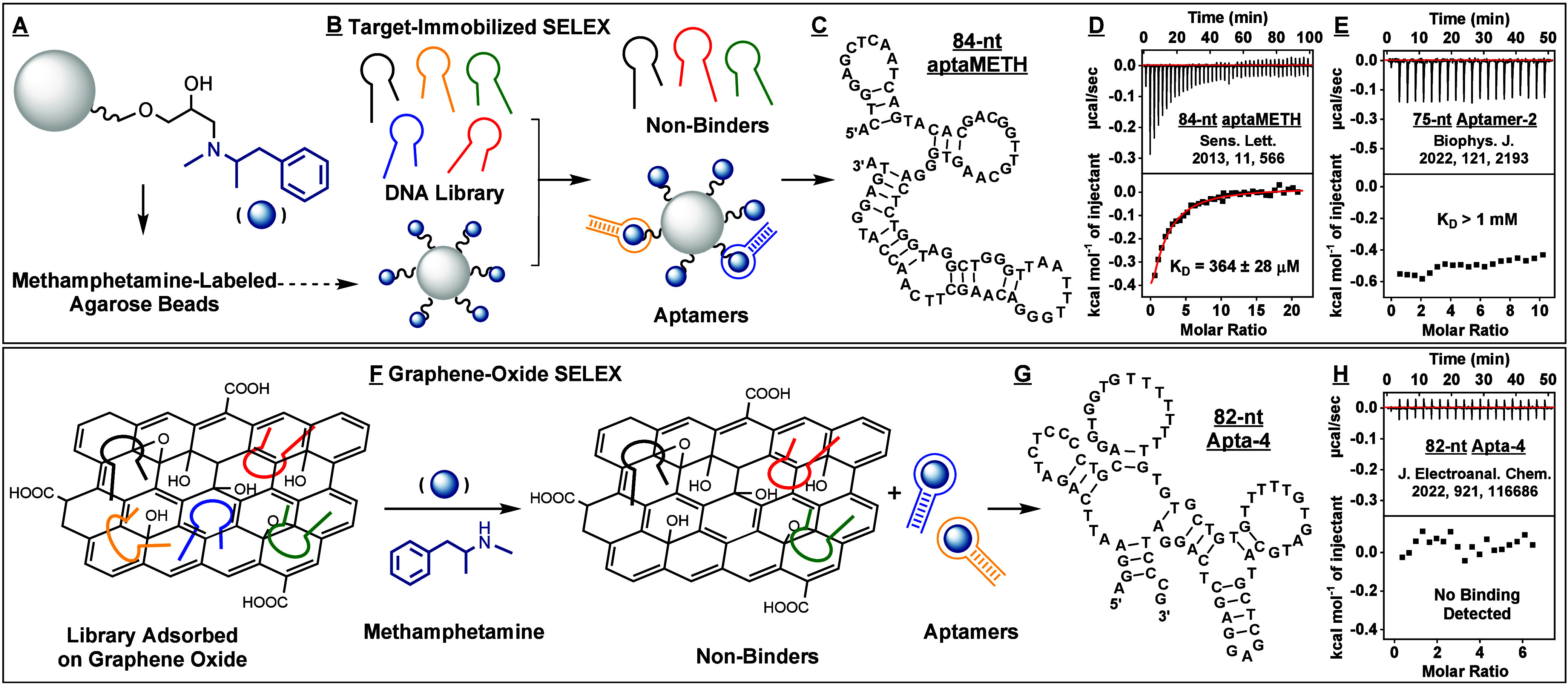
Affinity characterization of aptamers previously isolated for methamphetamine by other groups. Aptamers have been previously isolated using target-immobilized SELEX, in which (A) methamphetamine is conjugated to agarose beads via its amino group. (B) These beads are incubated with the DNA library, and binders are physically partitioned from binding-incompetent sequences. (C) Ebrahimi et al.^22^ identified 84-nt aptaMETH in this way and reported that this aptamer binds (+)-methamphetamine with a *K*_D_ of 100 nM. (D) However, our isothermal calorimetry (ITC) results showed a far higher *K*_D_ of 364 μM in their selection buffer. (E) Sester et al.^24^ used a similar approach to isolate 75-nt Aptamer-2, with a reported *K*_D_ of 244 nM, but our ITC results again indicated a higher *K*_D_ of >1 mM for (+)-methamphetamine in their reported selection buffer. (F) Bor et al.^23^ utilized graphene-oxide SELEX to isolate DNA aptamers for methamphetamine, based on binding-induced desorption of target-specific aptamers from graphene oxide. (G) The resulting 82-nt Apta-4 aptamer reportedly bound methamphetamine with a *K*_D_ of 1.3 μM. (H) In contrast, our ITC results indicate no binding at all in their reported selection buffer.

The first SELEX experiment to isolate aptamers for methamphetamine was conducted by Ebrahimi et al. more than a decade ago.^22^ They covalently attached methamphetamine onto epoxy-modified agarose via its amino group (Figure 1A) to partition aptamers from binding-incompetent sequences in a randomized N40 DNA library using target-immobilized SELEX (Figure 1B). The selection buffer consisted of 20 mM Tris (pH 7.4) with a relatively high ionic strength (200 mM NaCl and 5 mM MgCl_2_). After 14 rounds of SELEX, they identified aptaMETH (Figure 1C), an 84-nt aptamer that reportedly binds methamphetamine with a *K*_D_ of 100 nM as determined using a bead-based binding assay.^22^ To confirm the ability of this aptamer to bind methamphetamine, we synthesized aptaMETH (SI, Table S9) and utilized our exonuclease fluorescence assay^29^ to determine its relative target affinity. Here, aptamers are digested by T5 exonuclease (T5 Exo) and exonuclease I (Exo I); ligand-bound aptamers exhibit resistance to digestion that is proportional to their ligand-binding affinity. We observed that digestion was inhibited in the presence of racemic methamphetamine, indicating that the aptamer indeed binds to this target (SI, Figure S1). However, the degree of enzymatic inhibition was relatively low, with maximal inhibition occurring at an unexpectedly high concentration of 500 μM methamphetamine. To formally quantify the affinity of this aptamer, we used the gold-standard method isothermal titration calorimetry (ITC). Methamphetamine exists as two enantiomers: (+)- and (−)-methamphetamine, we assessed aptamer affinity to each enantiomer separately. We confirmed that aptaMETH binds (+)-methamphetamine, but its affinity is more than 3 orders of magnitude weaker (*K*_D_ = 364 ± 28 μM) (Figure 1D) than the previously reported *K*_D_ of 100 nM. The affinity of the aptamer for (−)-methamphetamine was too weak to confidently quantify (*K*_D_ > 1 mM), demonstrating that aptamer-target interactions are stereospecific (SI, Figure S2). The low enthalpy of binding indicated by ITC, coupled with the fact that the target was conjugated to a solid support during SELEX, indicates that the aptamer recognizes a part, but not the entirety, of the methamphetamine molecule. It is likely that the aptamer binds well to bead-immobilized methamphetamine (Figure 1A), but poorly to methamphetamine free in solution. However, Xie et al. recently utilized a truncated variant of aptaMETH (38-nt aptaMETH; SI, Table S9) to detect methamphetamine in an electrochemical aptamer-based sensor, with a reported limit of detection (LOD) of 30 nM.^30^ We performed ITC to determine if this truncated aptamer had affinity for methamphetamine, but did not observe any affinity for either (+)- or (−)-methamphetamine (SI, Figure S3). Similarly, Sester et al. performed target-immobilized SELEX to isolate a DNA aptamer for methamphetamine with an N40 random library in 2 mM Tris-HCl buffer with relatively low ionic strength (10 mM NaCl, 0.5 mM KCl, 0.2 mM MgCl_2_, and 0.1 mM CaCl_2_).^24^ Their highest-affinity aptamer (Aptamer-2; SI, Table S9) exhibited a *K*_D_ of 244 nM in a dye-displacement assay. However, in our exonuclease fluorescence assay, Aptamer-2 did not resist digestion even in the presence of 500 μM (+)-methamphetamine (SI, Figure S4), indicating minimal target binding. Our ITC data indicated that Aptamer-2 has very weak or no binding affinity for both enantiomers of methamphetamine (*K*_D_ > 1 mM) (Figure 1E, SI, Figure S5). We thus concluded that target-immobilized SELEX is not a suitable means of isolating high-affinity aptamers for a target like methamphetamine, with so few functional groups.

In another recent report, Bor et al. performed graphene-oxide SELEX to isolate DNA aptamers for methamphetamine from an N40 DNA library.^23^ The library was first nonspecifically absorbed onto a graphene oxide surface, after which the target was added. Aptamers capable of binding the target should desorb from graphene oxide and can be collected in the supernatant (Figure 1F). Their PBS selection buffer had a pH of 7.0 with moderate ionic strength (100 mM NaCl, 2.7 mM KCl, and 2 mM MgCl_2_). After eight rounds, they identified an 82-nt aptamer termed Apta-4 (Figure 1G, and SI, Table S9), and determined that it binds methamphetamine with a *K*_D_ of 1.3 μM based on ITC analysis.^23^ However, our exonuclease assay revealed that this aptamer did not resist enzymatic digestion, even in the presence of 500 μM racemic methamphetamine—again, indicating weak or no affinity (SI, Figure S6). Likewise, when we performed ITC under the same conditions as reported by Bor et al., we did not observe any affinity between Apta-4 and (+)-methamphetamine (Figure 1H) or (−)-methamphetamine (SI, Figure S7). Even when we performed ITC with a 3-fold higher aptamer and target concentration, we still did not observe any affinity (SI, Figure S8). These data indicate that Apta-4 does not bind to methamphetamine, and that the graphene-oxide SELEX effort had failed.

### First Trial of SELEX: An Alternative Partitioning Strategy–Library-Immobilized SELEX

**Figure 2:**
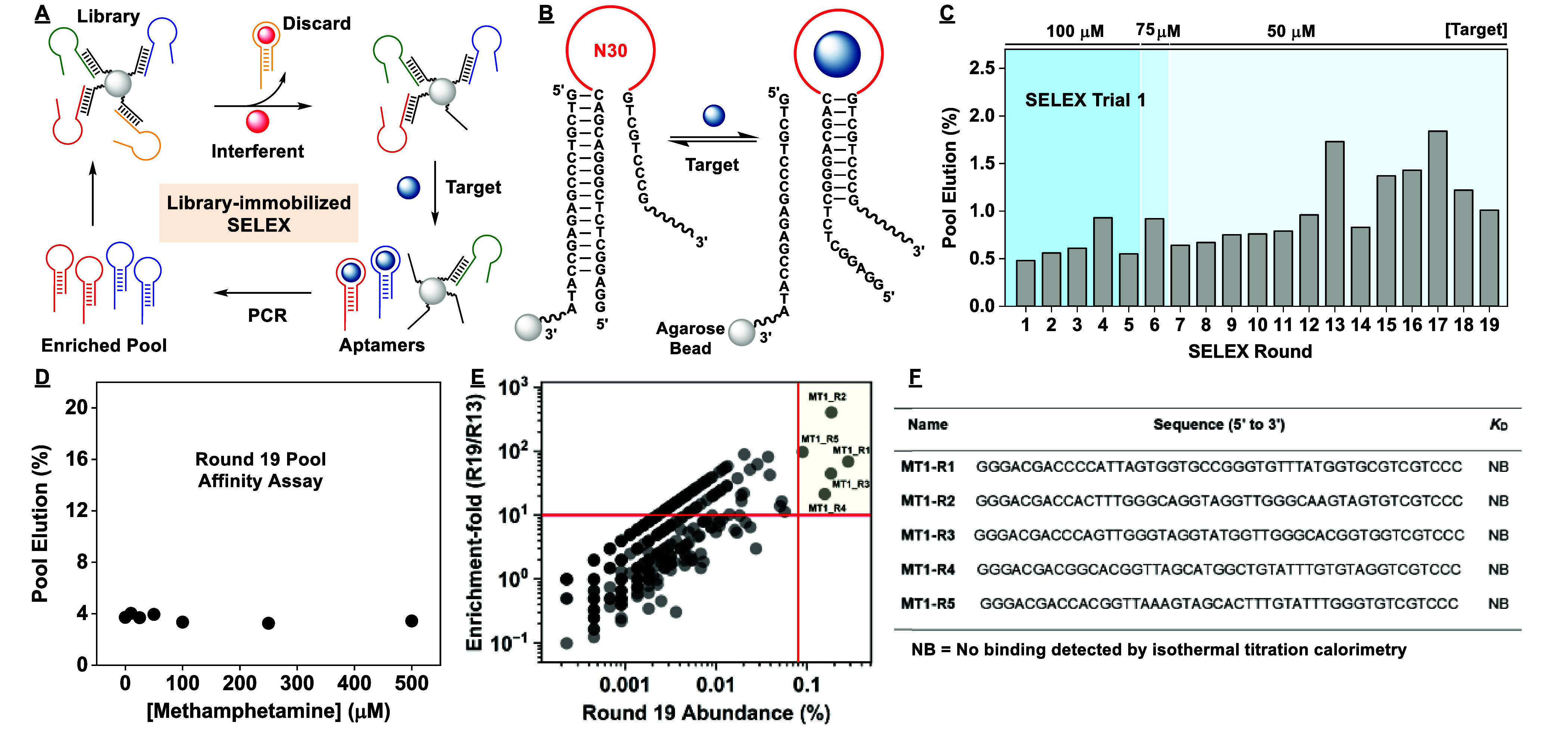
Results of the first SELEX trial. (A) Simplified scheme of library-immobilized SELEX. (B) The aptamer is initially hybridized to a biotinylated cDNA strand immobilized on agarose microbeads. Aptamer-target binding displaces the aptamer from the cDNA, releasing the aptamer into solution. (C) Pool elution in each round of the first SELEX trial. (D) Binding affinity of the Round 19 pool to (+)-methamphetamine was determined using a gel-elution assay. The pool displayed no apparent affinity for the target. (E) Round 13 and 19 pools were subjected to high-throughput sequencing (HTS). Enrichment-fold between Rounds 13 and 19 was plotted as a function of Round 19 abundance. Five top-ranked sequences (upper right) had abundance >0.08% and enrichment-fold >10. (F) None of these sequences displayed measurable affinity for (+)-methamphetamine based on ITC.

Having established the ineffectiveness of target- and graphene-oxide based SELEX, we performed library-immobilized SELEX to isolate DNA aptamers that bind (+)-methamphetamine. An advantage of this method relative to target-immobilized SELEX is that the target is not immobilized onto a solid support, allowing the aptamer and target to interact freely without masking functional groups on the target. The library is instead immobilized onto streptavidin-immobilized agarose beads via a biotinylated cDNA strand hybridized to the aptamer. Aptamers that bind the target dissociate from the cDNA and are released from the beads, after which they are amplified by PCR and used for the next round of selection (Figure 2A). We performed SELEX using a 73-nt stem-loop DNA library (Figure 2B) that we and others have used previously^17,31^ containing an 9-bp stem and a 30-nt random loop in buffer mimicking physiological conditions (1× PBS with 1 mM MgCl_2_). As the target, we used (+)-methamphetamine, which is the more pharmacologically potent of the two enantiomers. For the first five rounds, we used 100 μM (+)-methamphetamine, which we reduced to 50 μM thereafter. In the second round, we initiated counter-SELEX^32^ against a variety of interferents (SI, Figure S9) including ligands known to bind to three-way-junction structured oligonucleotides (e.g., procaine, lidocaine, and quinine); closely related analogs such as 4-hydroxymethamphetamine (4-HMA), amphetamine and 3,4-methylenedioxymethamphetamine (MDMA); other psychoactive drugs (e.g., heroin, fentanyl, and cocaine); structurally similar molecules (e.g., dopamine, bupropion, norepinephrine, and ephedrine); and endogenous compounds (e.g., serotonin, tyramine, tyrosine, and phenylalanine). We monitored the progress of SELEX by collecting aliquots of all eluents from washes with buffer, counter-target, or target, and performing polyacrylamide gel electrophoresis (PAGE) to quantify DNA in these aliquots. Monitoring pool eluted by target every round helps to determine whether target-binding aptamers are being enriched. Typically, as rounds progress, aptamers become more prevalent in enriched pools, and target-induced pool elution should increase. However, throughout the entirety of this trial, we observed consistently low pool elution by (+)-methamphetamine (1–2%), even after 19 rounds (Figure 2C). We also observed the Round 19 pool had no meaningful affinity for (+)-methamphetamine in a gel-elution assay^33^ (Figure 2D). To investigate this apparent failure more closely, we performed high-throughput sequencing (HTS) of the SELEX pools from this trial. The proportion of unique sequences did not change significantly between Rounds 13 and 19 (∼42%), indicating a lack of aptamer enrichment (SI, Table S6). We synthesized five top-ranked aptamer candidates for individual affinity characterization with an abundance >0.08% in Round 19 and an enrichment-fold >10 between Rounds 13 and 19 (Figure 2E, SI, Table S9, Trial 1). ITC indicated that none of these aptamers displayed measurable target affinity (Figure 2F, SI, Table S7, Trial 1, and Figure S10), clearly showing that this selection trial was unsuccessful.

### Second Trial of SELEX: Eliminating Counter-SELEX

**Figure 3:**
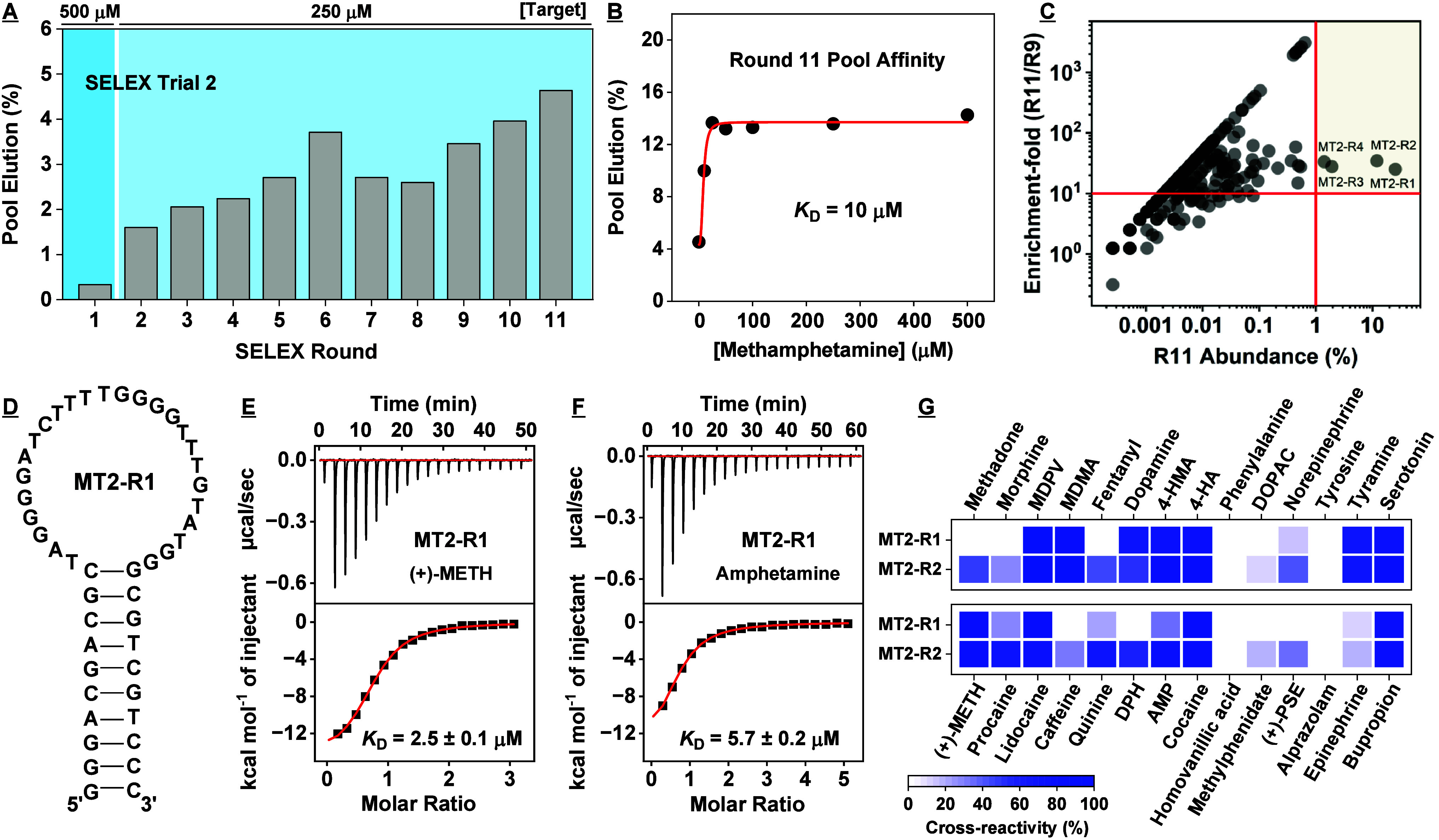
Results of the second SELEX trial. (A) Pool elution by target in each round of the second SELEX trial. (B) Binding affinity of the Round 11 pool to (+)-methamphetamine as determined using a gel-elution assay. (C) Enrichment-fold between Rounds 9 and 11 plotted as a function of Round 11 abundance. (D) Secondary structure of the most abundant aptamer discovered in this trial, MT2-R1. The binding affinity of MT2-R1 to (E) (+)-methamphetamine and (F) amphetamine was determined using ITC. (G) We assessed the specificity of MT2-R1 and MT2-R2 toward several interferents using the exonuclease digestion assay. Heat-map indicates cross-reactivity relative to (+)-METH. The concentration of target and interferent was 250 μM, except for alprazolam, which was 50 μM due to solubility limits.

These failures to select aptamers for (+)-methamphetamine by both us and others suggested that it may not be possible to isolate oligonucleotides for this target. However, we instead hypothesized that viable methamphetamine aptamers in the library were being removed by the very stringent counter-selection against structurally similar interferents like amphetamine. To determine whether this was true, we performed a second, lower-stringency trial of SELEX in which we used the same N30 library as the first trial, but omitted counter-SELEX. From Rounds 1–6, the quantity of pool eluted by (+)-methamphetamine slowly rose from 0.5% to 4%; by Round 11, pool elution reached nearly 5% (Figure 3A). We performed a gel-elution assay to determine the affinity of the Round 11 pool, and obtained a *K*_D_ of 10 μM, with a maximal pool elution of 17% at 25 μM (+)-methamphetamine (Figure 3B). Having apparently enriched binders to (+)-methamphetamine, we subjected the Round 9 and 11 pools to HTS to identify enriched aptamers. The proportion of unique sequences decreased from 39% in Round 9 to 22% in Round 11, which is a sign of aptamer enrichment (SI, Table S6, Trial 2). We synthesized four top-ranked aptamers with Round 11 abundance >1% and enrichment-fold >10 between Rounds 9 and 11 (Figure 3C, SI, Table S9, Trial 2) and determined their affinity for (+)-methamphetamine using ITC. These aptamers had micromolar affinities, with *K*_D_ ranging between 2.5–18 μM (SI, Table S7, Trial 2 and Figure S11), which indicated that oligonucleotides can indeed interact with (+)-methamphetamine with relatively good affinity. Unfortunately, our exonuclease assay showed that these aptamers had poor specificity. For instance, the highest affinity aptamer (MT2-R1) had a *K*_D_ of 2.5 ± 0.1 μM for (+)-methamphetamine (Figure 3E) but also had a *K*_D_ of 5.7 ± 0.2 μM for amphetamine (Figure 3E) and showed similar affinity for a wide range of other structurally similar molecules including lidocaine, cocaine, bupropion, MDPV, MDMA, dopamine, tyramine, and serotonin (Figure 3G and SI, Figure S12). The second most abundant aptamer, MT2-R2, likewise had poor specificity. These results indicate that these aptamers generally bind to compounds featuring both an aromatic moiety and an amino group. Therefore, while we were able to confirm the feasibility of isolating aptamers for (+)-methamphetamine, the poor specificity of these aptamers made them unusable. The SELEX results from Trials 1 and 2 imply that aptamers exhibiting both high affinity and specificity for methamphetamine were either very rare or nonexistent in the initial N30 library we employed.

### Third Trial of SELEX: Increasing Library Randomness

**Figure 4:**
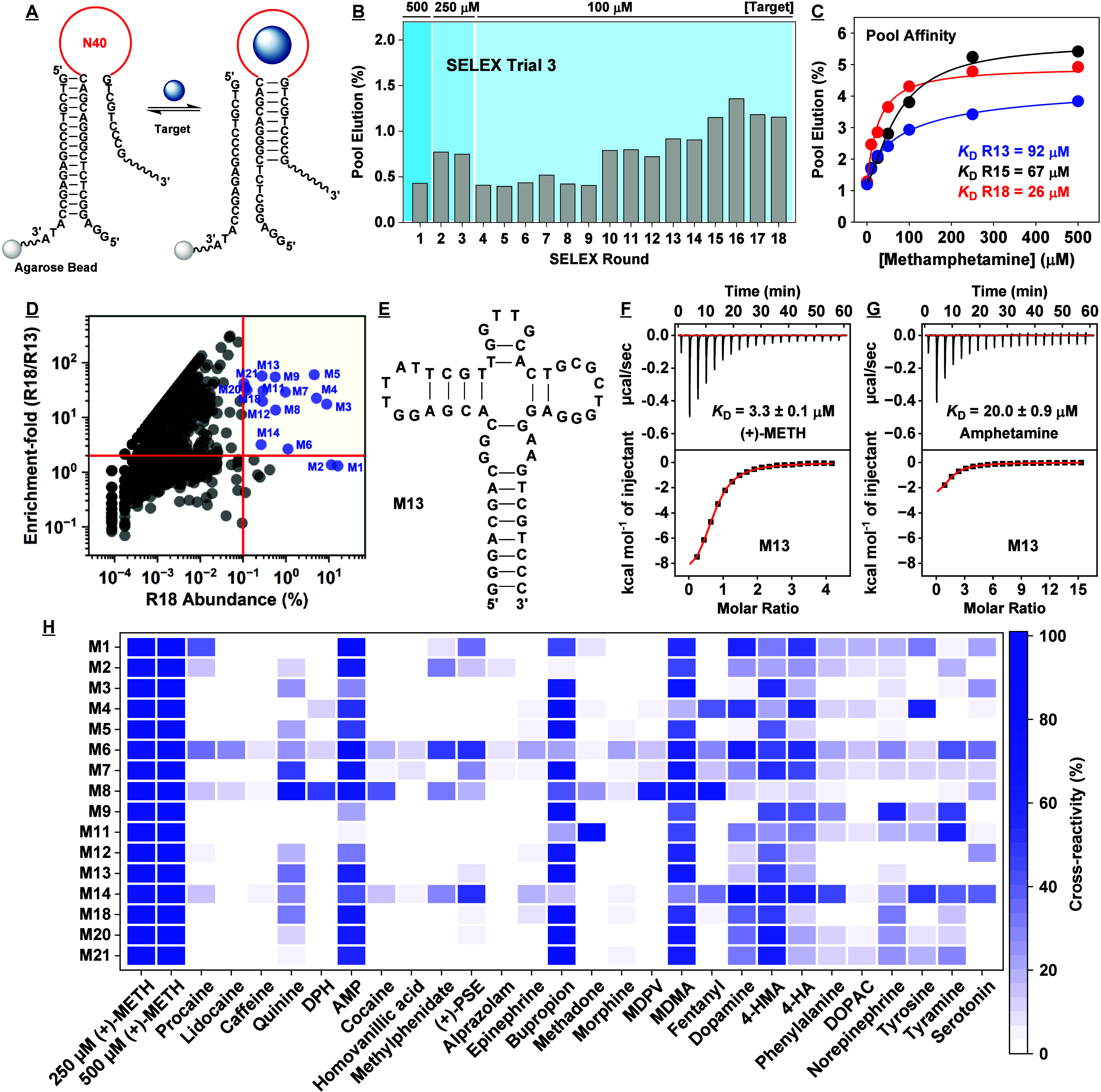
Results for the third SELEX trial. (A) In this trial, we employed an N40 library for library-immobilized SELEX. (B) Pool elution by (+)-methamphetamine for each round of SELEX. (C) Binding affinity for (+)-methamphetamine as determined using the gel-elution assay for the Round 13, 15, and 18 pools. (D) Enrichment-fold of sequences between Rounds 13 and 18 plotted as a function of Round 18 abundance. Sequences with abundance > 0.1% and enrichment-fold > 2 are named and marked in blue. (E) Secondary structure of one of the highly enriched aptamers, M13, as predicted by NUPACK. Binding affinity of M13 to (F) (+)-methamphetamine and (G) amphetamine as determined using ITC. (H) Specificity of aptamers discovered in this trial to a panel of interferents as assessed via exonuclease digestion assay. Heat-map indicates cross-reactivity relative to 250 μM (+)-METH. The concentration of interferent employed was 250 μM, but 100 μM for quinine and 50 μM for alprazolam due to solubility limitations.

Having established the potential to isolate (+)-methamphetamine-binding aptamers, our next goal was to obtain an aptamer with both high affinity and specificity for this target. However, we hypothesized that such an aptamer could not be found in an N30 library. This notion was based on two previous studies. The first, performed by the Szostak group, found that longer binding domains play an important role in improved aptamer binding performance.^34^ In the second study, the Stojanovic group successfully isolated aptamers for the small molecule γ-aminobutyric acid (GABA) using an N44 library after previously failing to do so with an N36 library.^19^ These reports suggested that a larger, higher-complexity binding pocket may be necessary for high-performance molecular recognition. Hence, for our third trial of SELEX, we used a new N40 stem-loop library (Figure 4A). From Rounds 1 to 7, we observed relatively static pool elution ranging between 0.4–0.7% (Figure 4B). We again employed counter-SELEX from Round 2 onward, and noticed that in Round 7 a sizable portion of the library was being eluted by procaine (1.5%), lidocaine (2.4%), diphenhydramine (1.4%), tyramine (1.2%), methylphenidate (1%), alprazolam (1.3%), bupropion (1.5%), and 4-hydroxymethamphetamine (4-HMA) (0.8%). This was expected, as all these molecules have a close resemblance to (+)-methamphetamine, with the exception of alprazolam. In Round 8, we performed additional counter-SELEX washes for structurally similar molecules such as amphetamine, 4-HMA, norepinephrine, bupropion, and MDMA, and observed increased pool elution in turn for these interferents. In Rounds 8 and 9, pool elution by (+)-methamphetamine remained stagnant at 0.4% (Figure 4B). However, in Round 10, target-induced pool elution doubled to 0.8% and remained similarly high in Rounds 11 and 12. This suggested that although the counter-SELEX stringency was relatively high, specific aptamers for (+)-methamphetamine could potentially continue to be enriched. Notably, in Round 13, target-induced pool elution increased again to 0.9% (Figure 4B), and elution by counter-targets such as diphenhydramine, 4-HMA, tyramine, and dopamine was reduced by half relative to previous rounds. This shift in the binding properties of the pool may indicate a change in the abundance of different aptamers in the pool. However, while pool elution rose again to 1.2% for (+)-methamphetamine in Round 15, we noted a sizable increase in pool elution by amphetamine (10%). We performed the gel-elution assay for the Round 13 and 15 pools to determine binding affinity to (+)-methamphetamine and specificity against the counter-targets. These pools bound to (+)-methamphetamine with a *K*_D_ of 92 and 67 μM with maximal pool elution of 3% and 5% at 500 μM (+)-methamphetamine, respectively (Figure 4C). However, the Round 15 pool responded to amphetamine, procaine, 4-HMA, tyramine, pseudoephedrine, norepinephrine, bupropion, and MDMA with cross-reactivity >30% relative to 500 μM target (SI, Figure S13A). While pool elution by target rose to 1.4% in Round 16, amphetamine eluted 12% of the pool, and this pattern and level of elution continued in Rounds 17 and 18 (Figure 4B). A gel-elution assay for the Round 18 pool revealed an improved *K*_D_ of 26 μM for (+)-methamphetamine (Figure 4C), however, the pool also responded to several counter-targets, such as amphetamine, with cross-reactivity >40% relative to 500 μM (+)-methamphetamine (SI, Figure S13B). Since pool specificity was not improving in these later selection rounds, we ended this trial of SELEX.

We subjected the SELEX pools from this trial to HTS. The percentage of unique sequences dropped from 46% to 31% between Rounds 7 and 13, and further decreased to 19% in Round 18, indicating that the pools were being enriched (SI, Table S6). To select aptamer candidates for binding characterization, we synthesized those with Round 18 abundance >0.1% and enrichment-fold >2 between Rounds 13 and 18, as well as the two most abundant sequences in the Round 18 pool (Figure 4D, SI, Table S9, Trial 3). We performed ITC to determine the affinity of 16 different aptamers, and obtained *K*_D_s ranging between 3.3–12.4 μM for (+)-methamphetamine (SI, Table S7, Figures S14–15) and 6.4 to 171 μM for amphetamine (SI, Table S7 and Figures S16–17). Therefore, the affinities of these aptamers were not meaningfully different from those discovered in the N30 pool, although our highest-affinity aptamer, M13 (Figure 4E), had 6-fold higher binding affinity for (+)-methamphetamine relative to amphetamine (Figure 4F, G). We next assessed the specificity of 11 of these aptamers using our exonuclease-based assay, and determined that essentially all aptamers had poor to moderate specificity, with cross-reactivity mainly apparent with amphetamine, quinine, bupropion, MDMA, 4-HMA, norepinephrine, phenylalanine, and dopamine (Figure 4H and SI, Figures S18–20). The aptamer with the best specificity, M4, had a *K*_D_ of 12 μM for (+)-methamphetamine with minimal cross-reactivity to all counter-targets except for amphetamine, bupropion, tyrosine, dopamine, MDMA, and 4-HMA. This was a notable improvement over the best aptamer from the previous trial of SELEX, which cross-reacted to at least 10 different compounds with similar affinity to (+)-methamphetamine. We therefore concluded that whereas an N40 library yielded aptamers with better specificity than N30 libraries, they were still not sufficiently specific.

### Fourth Trial of SELEX: Altering Buffer Conditions Leads to High-Performance Aptamers

**Figure 5:**
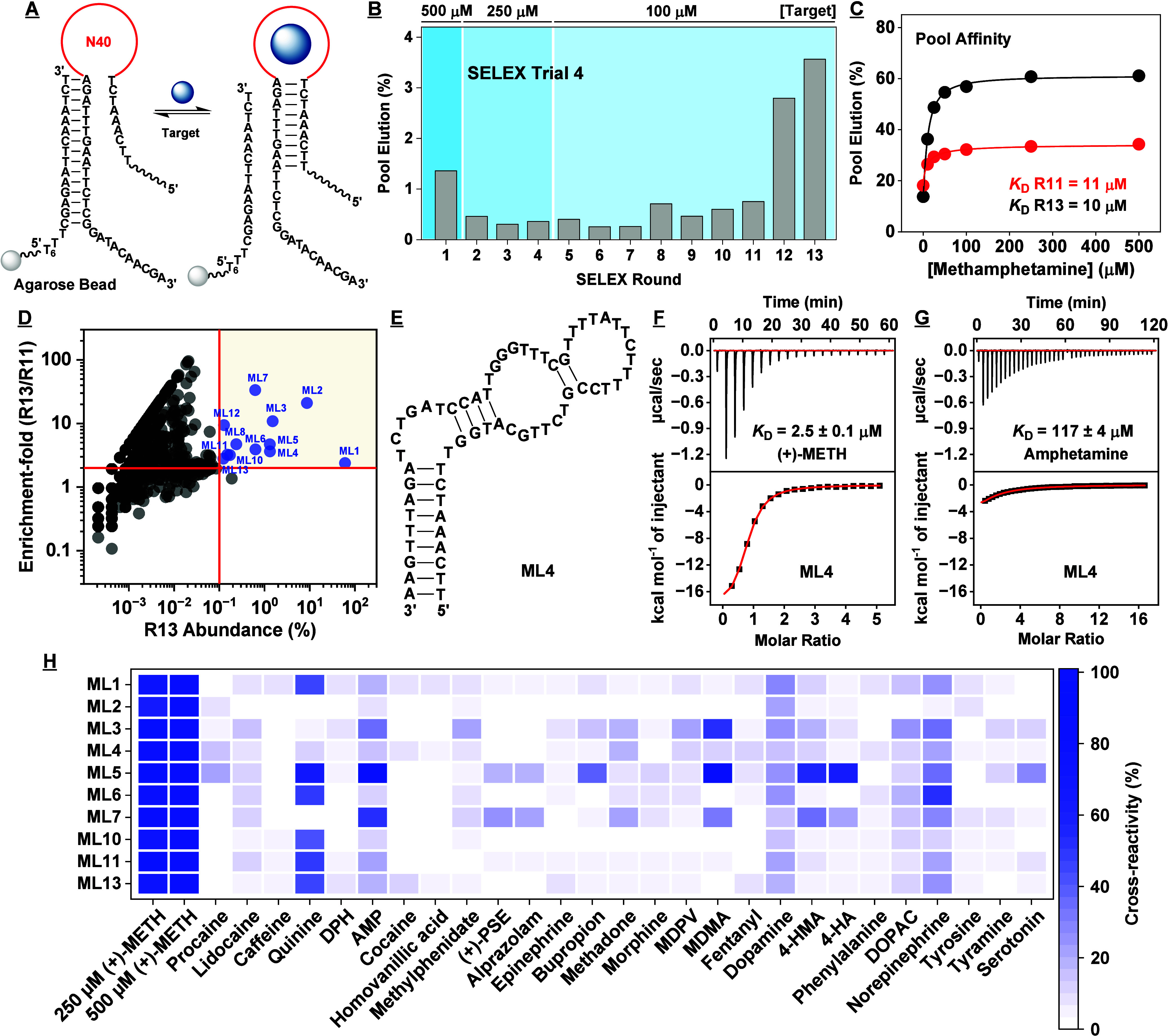
Results for the fourth SELEX trial. (A) In this trial, we employed an N40 library for library-immobilized SELEX. (B) Pool elution by the target for each round of SELEX. (C) Binding affinity for (+)-methamphetamine based on a gel-elution assay for the Round 11 and 13 pools. (D) Enrichment-fold of sequences between Rounds 11 and 13 plotted as a function of Round 13 abundance. Sequences with abundance >0.1% and enrichment-fold >2 are named and marked in blue. (E) Secondary structure of aptamer ML4 as predicted by NUPACK. Binding affinity of ML4 for (F) (+)-methamphetamine and (G) amphetamine as determined using ITC. (H) Specificity of aptamers discovered in this trial to a panel of interferents as assessed via exonuclease digestion assay. Heat-map indicates cross-reactivity relative to 500 μM (+)-methamphetamine. The concentration of interferent employed was 250 μM; alprazolam was 50 μM.

In our final trial of SELEX, we investigated if changing the composition of the selection buffer could yield higher quality aptamers. A systematic study by Carothers et al. determined that higher concentrations of Mg^2+^ (1 mM versus 5 mM) in the selection buffer led to the enrichment of higher-affinity aptamers.^35^ We similarly found in our own past selection of cocaine aptamers with an N30 library in buffer containing 5 mM Mg^2+^ yielded aptamers with 2.5-fold higher affinity^36^ than cocaine aptamers isolated by the Stojanovic group^37^ with an N36 library in buffer containing 2 mM Mg^2+^. For our fourth trial, we therefore increased the concentration of Mg^2+^ from 1 mM to 5 mM. Since 5 mM Mg^2+^ is insoluble in 1× PBS, we used 0.5× PBS instead. In addition, during our negative and counter-selection steps, we incorporated 0.005% (v/v) Triton in the selection buffer based on the presumption that it would increase the separation efficiency with which nonspecific binders are removed from the library. This was supported by preliminary testing with our naive N40 library, in which we observed a 2-fold increase in library elution after washing with Triton-containing versus Triton-free buffer (SI, Figure S21). In Round 1, pool elution of the N40 library by target was 1.4% (Figure 5B). In Round 2, we initiated counter-SELEX, this time including the problematic counter-targets amphetamine, bupropion, and MDMA earlier on and with more washes; we then introduced 4-HMA, norepinephrine, epinephrine, dopamine, and tyramine in Round 3. Pool elution by (+)-methamphetamine hovered between 0.3–0.4% in Rounds 2–7, and as in previous trials, counter-targets closely related to (+)-methamphetamine in structure such as amphetamine, 4-HMA, and MDMA eluted more pool than the target. In Round 8, we observed a shift in the binding properties of the pool, with relatively lower elution by counter-targets in general and a near-doubling of pool elution by (+)-methamphetamine to 0.7%. This general trend continued in Rounds 9–11. In Round 12, pool elution by (+)-methamphetamine surged to 2.8%, with only amphetamine and quinine eluting meaningful proportions (>1.5%) of the pool. Target-induced pool elution increased again in Round 13 to 3.6%, indicating the successful enrichment of (+)-methamphetamine binders. To assess pool binding properties, we performed the gel-elution assay for Rounds 11 and 13 and obtained a *K*_D_ of 11 and 10 μM for (+)-methamphetamine, respectively (Figure 5C), the highest pool affinity obtained thus far. We also determined the specificity of the Round 13 pool using the gel-elution assay and observed meaningful cross-reactivity only to quinine, 4-HMA, and pseudoephedrine (SI, Figure S22). Notably, the level of pool elution by amphetamine, dopamine, norepinephrine, epinephrine, and MDMA were similar to buffer alone, indicating a considerable improvement in pool specificity. Therefore, we concluded SELEX at this round.

HTS analysis of SELEX pools from this trial confirmed that (+)-methamphetamine-binding aptamers were enriched. The proportion of unique sequences decreased from 44% in Round 9 to 9.6% in Round 13 (SI, Table S6). To select aptamer candidates for further characterization, we chose 13 sequences that had a Round 13 abundance >0.1% and an enrichment-fold >2 between Rounds 11 and 13 (Figure 5D, SI, Table S9, Trial 4). Using ITC, we determined *K*_D_s for (+)-methamphetamine ranging between 1.3–8 μM (SI, Table S7 and Figures S23–24), which represents a clear improvement relative to aptamers identified in the previous trials. We also determined their affinity for amphetamine using ITC, and observed that all had lower affinity compared to (+)-methamphetamine (SI, Table S7 and Figures S25–26). The most specific aptamer, ML4 (K_D_ = 2.5 ± 0.1 μM) (Figure 5E, F), had a 50-fold lower affinity for amphetamine (K_D_ = 117 ± 4 μM) (Figure 5G). Next, we used the exonuclease fluorescence assay to determine aptamer specificity, and found that ML4 did not meaningfully respond to amphetamine, pseudoephedrine, bupropion, MDPV, MDMA, dopamine, phenylalanine, or tyramine, except to 4-HMA with 15% cross-reactivity (Figure 5H and SI, Figures S27–28). More generally, all aptamers had improved specificity relative to those identified from previous trials. The highest-affinity aptamer, ML7 (K_D_ = 1.30 ± 0.03 μM), also had high specificity, but had nearly 33% cross-reactivity to 4-HMA and 53% cross-reactivity to amphetamine. Collectively, these results confirm that high-quality aptamers for (+)-methamphetamine can indeed be isolated with an appropriately designed SELEX trial.

Since we employed a relatively high concentration of Mg^2+^ for selection, we next determined the importance of the concentration of this divalent cation on the binding affinity of the isolated aptamers. To do so, we performed ITC with ML3 and ML4 for (+)-methamphetamine in 0.5× PBS (pH 7.4) plus 1, 2, or 5 mM MgCl_2_. In general, the affinity of both aptamers increased as the concentration of Mg^2+^ increased. Specifically, the *K*_D_ of ML3 for (+)-methamphetamine was 34.3 μM, 9.9 μM, and 1.5 μM in 0.5× PBS buffer containing 1, 2, or 5 mM MgCl_2_ (SI, Figures S29A–C), respectively; for ML4, *K*_D_s were respectively 65.2 μM, 16.2 μM, and 2.5 μM (SI, Figures S29D–F). In comparison to the work by Carothers et al., the authors observed that the largest decrease in affinity (measured as ΔΔ*G*) when Mg^2+^ was decreased from 5 mM to 1 mM was ∼1,000 cal/mol.^35^ In contrast, our aptamers ML3 and ML4 experienced a much larger decrease in affinity, with a ΔΔ*G* of ∼1,800 cal/mol and 1,920 cal/mol, respectively. These results therefore indicate that ML3 and ML4 require Mg^2+^ to bind methamphetamine, and it is possible that this ion stabilizes a critical binding-competent conformation of the aptamers.

### High-Specificity Binding by (+)-Methamphetamine Aptamers and Comparison to Antibodies

For sensing applications, receptors for (+)-methamphetamine must be able to reject structurally similar interferent molecules. It is well-documented in the literature that immunoassays for (+)-methamphetamine often cross-react to analogs like amphetamine, bupropion, and MDMA.^38−40^ In stark contrast, our aptamer ML4 rejects these structurally similar molecules, which indicates that aptamers have superior specificity over antibodies for this target. The capability of aptamers like ML4 to discriminate between (+)-methamphetamine and amphetamine is quite impressive, as they only differ by a single methyl group. Similar discrimination has been seen with a previously published theophylline aptamer that favors theophylline binding 10,000-fold relative to caffeine, which also differs by a single methyl group.^32^ However, recognizing (+)-methamphetamine but not amphetamine is arguably a more challenging feat, because the methyl group of (+)-methamphetamine leaves its amino group with one less hydrogen bond, which one would expect to result in weaker affinity. Despite this, we see that ML4 prefers (+)-methamphetamine relative to amphetamine, by 50-fold (Figure 5G). While it has been documented that some antibodies can distinguish between (+)-methamphetamine and amphetamine, these antibodies are unable to discriminate between (+)-methamphetamine and MDMA and other analogs.^41−43^ To determine whether our aptamers could achieve this level of molecular discrimination, we quantified the affinity of ML4 to a variety of structurally similar interferents using ITC. Impressively, we found that ML4 has 180-, 100-, 150-, 90-, 300-, and 140-fold lower affinity for (−)-methamphetamine (*K*_D_ = 450 ± 21 μM), 4-HMA (*K*_D_ = 255 ± 8 μM), (+)-pseudoephedrine (*K*_D_ = 372 ± 24 μM), MDMA (*K*_D_ = 223 ± 8 μM), methylphenidate (*K*_D_ = 749 ± 10 μM), and MDPV (*K*_D_ = 344 ± 23 μM), respectively, relative to (+)-methamphetamine (SI, Figures 30–31). Based on three-dimensional structures of reported high-affinity riboswitches,^44,45^ we hypothesize that this improved specificity from aptamers may arise because they have greater flexibility and can completely envelope their small molecule target, thus enforcing strict requirements for guest size, shape, and electrostatics.

### Analysis of the SELEX Trials and Comparison of (+)-Methamphetamine Aptamers

Through the four independent SELEX experiments performed here, we have demonstrated the dramatic influence that different selection parameters have on the outcome of SELEX. In the first trial using an N30 library, where we performed counter-SELEX with numerous structurally similar compounds, we were not able to identify any aptamers. When we skipped counter-SELEX in the second trial, we discovered several (+)-methamphetamine aptamers with an average K_D_ of 8.9 ± 6.9 μM for (+)-methamphetamine, but these aptamers cross-reacted to more than a dozen other molecules, such as amphetamine, MDMA, 4-HMA, 4-HA, and lidocaine. This suggests that there were most likely no highly specific sequences in the N30 library that could bind (+)-methamphetamine while also rejecting structurally similar interferent molecules. In the third trial, we employed an N40 library and restored the counter-SELEX process, and observed a notable improvement in aptamer affinity and specificity. We obtained aptamers with an average K_D_ of 6.6 ± 2.3 μM for (+)-methamphetamine, with specificities that were likewise improved relative to the previous trial. Indeed, the best aptamer only cross-reacted to five of our nontarget molecules. In the final trial, we adjusted buffer ionic conditions and the counter-SELEX process with an N40 library, and we were able to isolate higher affinity and specific aptamers with an average K_D_ of 3.6 ± 2.4 μM. For the best aptamer, we observed near-perfect specificity, with only minor cross-reactivity (<20%) to 4-HMA. These findings suggest that the recognition of low-complexity small molecules such as (+)-methamphetamine may require relatively larger aptamers to achieve high-affinity, highly specific recognition.

**Figure 6:**
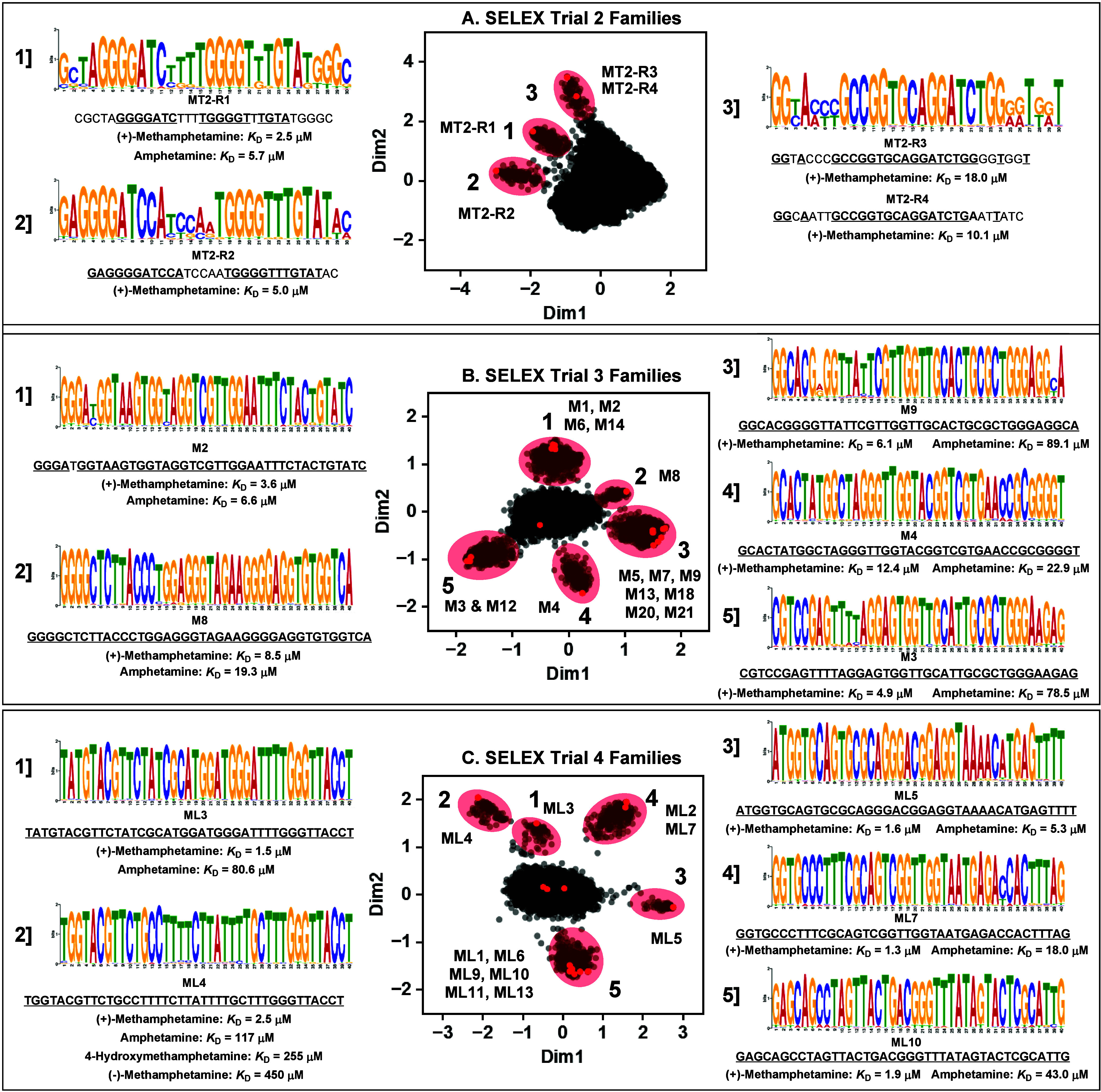
Identification of aptamer families and conserved sequence motifs from each trial of SELEX for (+)-methamphetamine via bioinformatic analysis. Families and motifs discovered in the final pool of the (A) second, (B) third, and (C) fourth SELEX trials. Plots in the middle represent the sequence space produced by Raptgen, with each dot representing a unique sequence. Aptamers close to each other in space are related to each other in sequence. Aptamer families in these plots are highlighted in red, and representative members are named. The primary motif in each family was determined using GLAM2, and a representative aptamer of that family is listed below the sequence logo along with its target-binding affinity and affinity for structurally related analogs of (+)-methamphetamine.

Analysis of the HTS data from the different trials provides some clues for the basis of the differing binding properties of the resulting (+)-methamphetamine aptamers. To demonstrate this, we used the bioinformatics software Raptgen to identify families in each trial based on their sequence similarity. Raptgen uses variational autoencoders to map HTS data onto a low-dimensional latent space, enabling the convenient identification of families with conserved motifs based on the formation of clusters in two-dimensional plots.^27^ To gain structural insights, we then used NUPACK^46^ to predict the secondary structures of a few aptamers from each family (SI, Figure S32). For the second SELEX trial, we identified three different families of sequences (Figure 6A). The first family contained a 17-nt consensus sequence flanked by regions of low consensus. The other two families were similar in sequence, containing two high-consensus GGGG repeats linked by four or five nucleotides of low consensus. Only 50–60% of the 30 nt binding domain was highly consensus-prone, indicating that only a portion of the binding domain is crucial for target recognition. We therefore characterized these families as having low complexity—and unsurprisingly, these aptamers also had the poorest affinity and specificity among all the aptamers we identified. This relationship between binding performance and sequence complexity mirrors previous studies showing that sequences with low information content have inferior binding properties relative to those that are more information-rich.^34^ In contrast, when we applied Raptgen to the final-round SELEX pool from the third trial, we identified five different families, all of which had high-consensus regions spanning nearly the entire binding domain (∼90%) (Figure 6B). As expected, these sequences also had better affinity and specificity than aptamers from N30 libraries, further supporting the notion that more information-rich libraries with larger randomized domains yield aptamer candidates with superior overall binding properties. When examining the final SELEX pool from the fourth trial, we observed five families that all had very high consensus (>95% conservation) for all 40 nt (Figure 6C). These aptamers had the best affinity and specificity, with some aptamers (e.g., ML3 and ML4) capable of discriminating amphetamine and (−)-methamphetamine from (+)-methamphetamine with a ≥ 50-fold affinity difference. This provides strong evidence that the more nucleobases that are involved in target recognition, the better the binding performance of the aptamer. Notably, the best performing aptamer, ML4, was a member of a family that was unusually T-rich: > 50% of the nucleobases were Ts. However, there were no more than three As in the sequences from this family, implying that these Ts were not participating in canonical A-T Watson–Crick pairing but were instead contributing to a noncanonical structure or directly involved in target recognition. Other well-performing aptamers were members of another distinct family that was relatively T-rich (40%), but with greater representation of A and G bases. This indicates that are multiple different oligonucleotide architectures capable of (+)-methamphetamine recognition. Finally, we saw no overlap in sequences between any of the SELEX trials, which is unsurprising since N40 libraries have a theoretical sequence space of 10^24^—well beyond our initial sampling of 10^14^ library sequences.

To more clearly define the contribution of various nucleobases in ML3 and ML4 for target recognition, we designed various point mutants of ML3 and ML4 and determined their affinity for (+)-methamphetamine using the exonuclease digestion assay and ITC. For ML3, we created six different mutants by either changing C16 to A, T19 to A, G34 to T, T39 to A, G41 to T, or T49 to A, which we termed ML3-mut1, -mut2, -mut3, -mut4, -mut5, and -mut6, respectively (SI, Figure S33A and Table S10). The exonuclease assay indicated that ML3-mut1, -mut5, and -mut6 had little or no affinity for methamphetamine, while ML3-mut2 and -mut4 had weaker affinity relative to ML3, and ML3-mut3 retained similar affinity to ML3 (SI, Figure S33B). The ITC data corroborated well with the exonuclease assay, with K_D_s for (+)-methamphetamine of 408 ± 16 μM, 39 ± 2 μM, 1.4 ± 0.1 μM, 73 ± 3 μM, > 1 mM, and >1 mM for ML3-mut1, -mut2, -mut3, -mut4, -mut5, and -mut6, respectively (SI, Figures S33C–H and Table S8). These data indicate that C16, G41, and T49 are essential for methamphetamine binding. For ML4, we were interested in determining the role of the thymine bases in this pyrimidine-rich aptamer. Therefore, we created seven different point mutants by respectively changing T18, C23, T25, T27, T33, T35, and T39 to A (termed ML4-mut1, -mut2, -mut3, -mut4, -mut5, -mut6, and -mut7, respectively) (SI, Figure S34A and Table S10). The exonuclease assay indicated that only ML4-mut1 and -mut7 had heavily impaired affinity relative to ML4, with ML4-mut5 and -mut6 only having slightly lower affinity (SI, Figure S34B). Again, ITC data corroborated well with these data from the enzyme assay, with K_D_s of 67 ± 4 μM, 4 ± 0.1 μM, 3.2 ± 0.1 μM, 4.9 ± 0.2 μM, 3.3 ± 0.1 μM, 3.8 ± 0.1 μM, 64 ± 4 μM for (+)-methamphetamine (SI, Figure S34C–I and Table S8), indicating that T18 and T39 are important contributors to target binding.

### Detection of (+)-Methamphetamine in Oral Fluid with an Aptamer-Based Dye Displacement Assay

**Figure 7:**
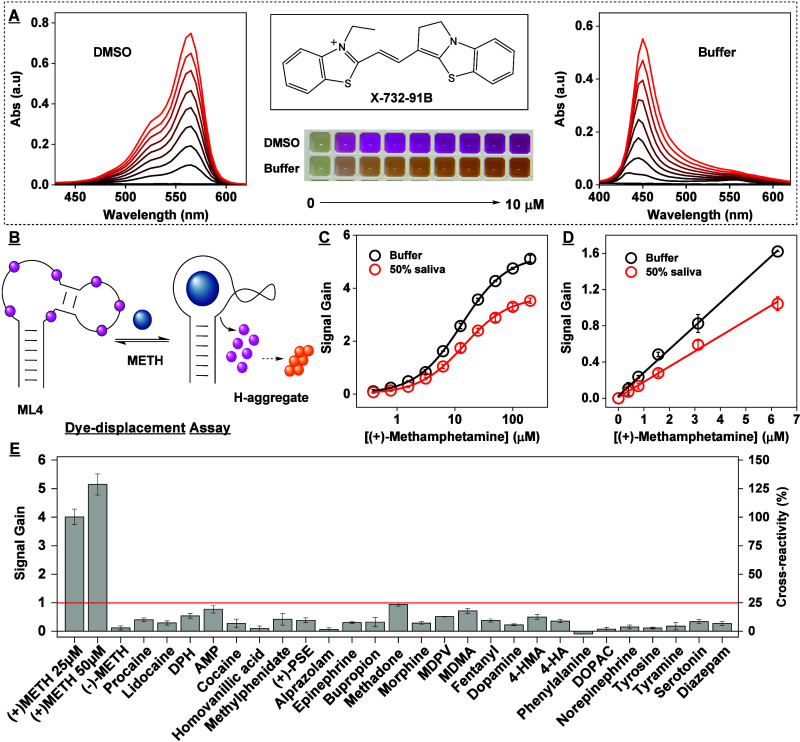
Colorimetric detection of (+)-methamphetamine with an aptamer-based dye-displacement assay. (A) Absorbance spectra of the cyanine dye X-732–91B dissolved in DMSO (left) and aqueous buffer (right) at concentrations of 0–10 μM. The structure of the dye is shown at the top center, and a photograph of solutions containing various concentrations of dye is shown at bottom center. (B) Scheme of the dye-displacement assay using aptamer ML4 and X-732–91B. Target binding displaces the dye from the aptamer into solution, causing the dye to aggregate and inducing a concomitant color change. (C) Calibration curve of this assay in both buffer (black) and 50% saliva (red). (D) Response of the assay to 0–6.4 μM (+)-methamphetamine. (E) Assay cross-reactivity to 50 μM interferents. The red line demarcates 25% cross-reactivity relative to 25 μM (+)-methamphetamine.

Finally, we demonstrated that our new aptamers are capable of rapid and facile detection of (+)-methamphetamine in oral fluid. We developed a dye-displacement assay^47^ based on aptamer ML4 and the cyanine dye X-732–91B. This dye maximally absorbs at 568 nm as a monomer in DMSO, producing a hot-pink color (Figure 7A), but forms H-aggregates with an absorbance maximum at 450 nm in aqueous solution, yielding a yellow color. When the dye is titrated with increasing concentrations of aptamer in aqueous buffer, the absorbance peak at 450 nm diminishes while the peak at 568 nm grows, indicating the conversion of free aggregates to aptamer-bound monomers (SI, Figure S35). When we titrated (+)-methamphetamine into a mixture of 6 μM aptamer and 4 μM dye, the target displaces the dye from the aptamer-dye complexes and these released dye molecules form H-aggregates in solution (Figure 7B). This resulted in a nearly immediate decrease in monomer absorbance and an increase in H-aggregate absorbance. We quantified the concentration of (+)-methamphetamine based on the ratio of aggregate:monomer absorbance, obtaining an instrumental LOD of 390 nM and a linear range of 0–6.25 μM (Figure 7C–D). In a control experiment, we confirmed that (+)-methamphetamine did not affect the absorbance spectrum of the dye itself (SI, Figure S36). Notably, this assay worked equally well in both buffer and 50% saliva (Figure 7C–D, SI, Figure S37) with identical detection limits. We also determined the response of the assay to a panel of structurally similar compounds and common drugs of abuse and did not observe any meaningful cross-reactivity (Figure 7E and SI, Figure S38). We finally demonstrated that the assay can specifically detect (+)-methamphetamine even when it is present in a mixture of drugs. Specifically, we observed that the response of the assay to 2.5 μM or 5 μM (+)-methamphetamine was similar whether the target was alone or in a mixture of 1 μM morphine, 3 μM cocaine, 2 μM methadone, and 1 μM fentanyl. Additionally, the assay did not respond to the drug mixture itself (SI, Figure S39). These results thus demonstrate that this assay is highly specific for (+)-methamphetamine.

The concentration of methamphetamine in human saliva can reach ∼1–3 μM between 2–4 h after consumption and declines slowly thereafter, reaching ∼0.5–0.8 μM at 8 h postconsumption, and ∼0.1 μM by 24 h.^48−50^ Accordingly, we hypothesized that our assay can be used to determine recent (+)-methamphetamine use in oral fluid. To demonstrate this, we determined the response of the assay to clinically relevant methamphetamine concentrations and interferent concentrations that are, in some cases, 100-fold higher than maximum clinically relevant levels. We observed a clearly detectable signal at this target concentration, with <20% cross-reactivity to almost all interferents; we saw 26% cross-reactivity to a 6-fold higher concentration of MDMA (15 μM) relative to 2.5 μM (+)-methamphetamine, and 24% cross-reactivity to procaine at a 16-fold higher concentration (40 μM) (SI, Figure S40). Thus, our assay is specific enough to detect methamphetamine in saliva for clinical/toxicological purposes.

## Discussion

Here, we have systematically investigated the difficulties associated with isolating high-performance aptamers for the small-molecule target (+)-methamphetamine via *in vitro* selection, and identified strategies to successfully isolate aptamers for such targets. We first confirmed that previously reported aptamers for methamphetamine have either low or no affinity for this target, and hypothesized that this is due to the unsuitability of the selection strategies employed to isolate these aptamers. These include target-immobilized SELEX, which masks functional groups on targets and hence prevents aptamers from fully interacting with the target, and graphene-oxide SELEX, which has an order of magnitude lower separation efficiency than library-immobilized SELEX based on recent findings from the Liu group.^51^ Here, we studied these aptamers using ITC. Since in some cases ligand binding does not release a meaningful amount of heat, ITC will not be able to determine binding affinity. There are alternative gold standard affinity determination methods such as surface plasmon resonance, biolayer interferometry, and microscale thermophoresis. However, we presumed that the aptamer-methamphetamine binding may be too subtle to produce a detectable signal in these platforms (i.e., a change in surface refractive index, biolayer thickness, or thermophoretic mobility) due to the low molecular weight of the target (149 Da). For this reason, we utilized our exonuclease digestion assay, a well-established approach for determining aptamer-ligand binding properties,^52^ to support our findings, which we found to be concordant with our ITC results.

We next demonstrated that the selection of high-performance aptamers for low-epitope targets such as methamphetamine is challenging. After performing four SELEX trials, we identified several strategies to facilitate isolation of aptamers for this target. In the first trial of SELEX using an N30 library where we performed stringent counter-SELEX, we observed that we could not enrich any aptamers for (+)-methamphetamine. This was most likely because there were no highly specific (+)-methamphetamine aptamers in the N30 library, and performing counter-SELEX against amphetamine and MDMA removed all of these (+)-methamphetamine aptamers from the pools. In the second trial, we performed SELEX without counter-SELEX to determine if aptamers could be isolated for (+)-methamphetamine, and found that it was indeed possible. Thus, we advise that when SELEX is initially performed for a low-complexity target, it may be preferable to withhold counter-SELEX to determine whether aptamers for that target can be enriched and how specific those aptamers could be. If these aptamers exhibit poor specificity, this information can be used to determine which molecules to include for counter-SELEX in the next trial. We also found that the discovery of highly specific aptamers for a low-complexity molecule like (+)-methamphetamine requires libraries with longer random regions, given our success with the N40 libraries used in the third and fourth trials relative to the failures with N30 libraries in the first two trials. This is in agreement with increasing evidence from the literature that high-quality aptamers for small molecule analytes with few epitopes can only be discovered with more complex random libraries. Additionally, if a selection fails, one should consider altering the ionic strength of the buffer; in particular, adjusting the concentration of Mg^2+^ can increase the likelihood of successfully identifying aptamers by influencing the conformation—and hence the function—of nucleic acids. Finally, the inclusion of surfactants like Triton X-100 in the selection buffer seems to increase the efficiency with which binding-incompetent sequences are eliminated during library-immobilized SELEX, although this will require further testing to verify. If there are concerns about the influence of surfactants on target dissolution or stability, these surfactants can be excluded from the buffer when the library is challenged with the target, as we have done in the present work. We would also like to note that this work specifically demonstrates the difficulty in generating aptamers for one particular low-epitope target, methamphetamine, and we hypothesize that selections performed against other challenging targets will require their own specific optimization process, as has been recently demonstrated by the Stojanovic group.^19^

## Supporting Information Available

List of DNA and aptamer sequences used in this work; selection strategies and conditions for four trials of methamphetamine SELEX; HTS statistics from SELEX trials 1–4; ITC conditions and results for methamphetamine aptamers; assessing the binding of previously reported methamphetamine aptamers using an exonuclease digestion assay and ITC; chemical structures of the interferents used in this work; binding affinity of aptamers discovered in SELEX trials 1–4 for (+)-methamphetamine and amphetamine based on ITC; specificity of isolated aptamer candidates using our exonuclease digestion assay; specificity of enriched pools as determined using a gel-elution assay; effect of including Triton X-100 in the selection buffer on washing efficiency during library-immobilized SELEX; binding affinity of ML4 for interferents based on ITC; secondary structures of methamphetamine aptamers obtained from trials 2–4; mutational analysis of ML3 and ML4; binding of dye X-732–91B to aptamer ML4 and target detection using dye X-732–91B and aptamer ML4; effect of (+)-methamphetamine on the absorbance spectrum of dye X-732–91B; detection of (+)-methamphetamine in a mixture of interferents using the dye-displacement assay; specificity of a dye-displacement assay for (+)-methamphetamine based on dye X-732–91B and aptamer ML4 (PDF)
