# Supplementary material for: Examining the Relationship between Aptamer Complexity and Molecular Discrimination of a Low-Epitope Target
Source: ACS Cent Sci. 2024 Nov 11;10(12):2213–28. doi: 10.1021/acscentsci.4c01377 (PMC11672540; doi:10.1021/acscentsci.4c01377)
Supplement: Supplementary file 1 — oc4c01377_si_001.pdf [file oc4c01377_si_001.pdf]

Supporting Information for:

Examining the Relationship Between Aptamer Complexity and Molecular Discrimination of a Low-Epitope Target

Linlin Wang, Juan Canoura, Caleb Byrd, Thinh Nguyen, Obtin Alkhamis, Phuong Ly and Yi Xiao*

Department of Chemistry, North Carolina State University, 2620 Yarbrough Dr., Raleigh, NC, USA, 27695

*Corresponding author: yxiao34@ncsu.edu

Table S1. List of DNA sequences used for SELEX.

Name	Sequence
Trial 1 & 2 Library	GGAGGCTCTCGGGACGAC (N30) GTCGTCCCGCCTTTAGGATTTACAG
Trial 3 Library	GGAGGCTCTCGGGACGAC (N40) GTCGTCCCGCCTTTAGGATTTACAG
Trial 4 Library	TCCATAGCTCCTTGTTCAAATCT (N40) AGTCTAGATTTGAATTCTCGGATACAACGA
Trial 1–3 FP	GGAGGCTCTCGGGACGAC
Trial 1–3 RP-bio	/5Bio/-CTGTAAATCCTAAAGGCGGGACGAC
Trial 1–3 cDNA15-bio	GTCGTCCCGAGAGCCATA-/3Bio/
Trial 1–3 FP-HTS	ACACTCTTTCCCTACACGACGCTCTTCCGATCT (N45) GGAGGCTCTCGGGACGAC
Trial 1–3 RP-HTS	GACTGGAGTTCAGACGTGTGCTCTTCCGATCT (N43) CTGTAAATCCTAAAGGCGGGACGAC
Trial 4 FP	TCCATAGCTCCTTGTTCAAATCT
Trial 4 RP-bio	/5Bio/TCGTTGTATCCGAGAATTCAAATCT
Trial 4 cDNA15-bio	/5Bio/CGAGAATTCAAATCT
Trial 4 FP-HTS	ACACTCTTTCCCTACACGACGCTCTTCCGATCT (N44) TCCATAGCTCCTTGTTCAAA
Trial 4 RP-HTS	GACTGGAGTTCAGACGTGTGCTCTTCCGATCT (N45) TCGTTGTATCCGAGAATTCA

/5Bio/ = 5′ biotin modification; /3Bio/ = 3′ biotin modification.

Table S2. Selection strategy and conditions for the first trial of methamphetamine SELEX.

Round	Pool Size (pmol)	Buffer Wash (×)	Counter-SELEX targets	Buffer Wash (×)	[Target] (µM)
**1**	1,000	10	None	None	100
**2**	447	20	100 µM PRO (250 µL ×3), 100 µM LIDO (250 µL ×3), 100 µM CAF (250 µL ×3), 100 µM PMZ (250 µL ×3), 100 µM QUI (250 µL ×3), 100 µM DPH (250 µL ×3), 100 µM FEN (250 µL ×3), 100 µM COC (250 µL ×3)	28	100
**3**	300	20	100 µM PRO (250 µL ×3), 100 µM LIDO (250 µL ×3), 100 µM CAF (250 µL ×3), 100 µM PMZ (250 µL ×3), 100 µM QUI (250 µL ×3), 100 µM DPH (250 µL ×3), 100 µM FEN (250 µL ×3), 100 µM COC (250 µL ×3), 100 µM AMP (250 µL ×3),100 µM DOP (250 µL ×3), 100 µM 4-HMA (250 µL ×3), 100 µM TYM (250 µL ×3), 100 µM 4-HA (250 µL ×3), 100 µM SER (250 µL ×3)	30	100
**4**	300	20	100 µM PRO (250 µL ×3), 100 µM LIDO (250 µL ×3), 100 µM CAF (250 µL ×3), 100 µM PMZ (250 µL ×3), 100 µM QUI (250 µL ×3), 100 µM DPH (250 µL ×3), 100 µM FEN (250 µL ×3), 100 µM COC (250 µL ×3), 100 µM AMP (250 µL ×3),100 µM DOP (250 µL ×3), 100 µM 4-HMA (250 µL ×3), 100 µM TYM (250 µL ×3), 100 µM 4-HA (250 µL ×3), 100 µM SER (250 µL ×3), 100 µM DOPAC (250 µL ×3), 100 µM HVA (250 µL ×3)	30	100
**5**	300	20	100 µM PRO (250 µL ×3), 100 µM LIDO (250 µL ×3), 100 µM PMZ (250 µL ×3), 100 µM CAF + QUI (250 µL ×3), 100 µM DPH (250 µL ×3), 100 µM FEN (250 µL ×6), 100 µM COC (250 µL ×3), 100 µM DOPAC + HVA (250 µL ×3), 100 µM PSU (250 µL ×3), 100 µM NE + EPI (250 µL ×3), 100 µM BPP (250 µL ×3), 100 µM METD + MOR (250 µL ×3), 100 µM MDPV + MDMA (250 µL ×3),100 µM AMP (250 µL ×3),100 µM DOP (250 µL ×3), 100 µM 4-HMA (250 µL ×3), 100 µM TYM (250 µL ×3), 100 µM 4-HA (250 µL ×3), 100 µM SER (250 µL ×3)	30	100
**6**	300	30	100 µM PRO (250 µL ×3), 100 µM LIDO (250 µL ×3), 100 µM PMZ (250 µL ×3), 100 µM CAF + QUI (250 µL ×3), 100 µM DPH (250 µL ×3), 100 µM FEN (250 µL ×6), 100 µM COC (250 µL ×3), 100 µM DOPAC + HVA (250 µL ×3), 100 µM MPD (250 µL ×3), 100 µM PSU (250 µL ×3), 100 µM NE + EPI (250 µL ×3), 100 µM BPP (250 µL ×3), 100 µM METD + MOR (250 µL ×3), 100 µM MDPV + MDMA (250 µL ×3),100 µM AMP (250 µL ×3),100 µM DOP (250 µL ×3), 100 µM 4-HMA (250 µL ×3), 100 µM TYM (250 µL ×3), 100 µM 4-HA (250 µL ×3), 100 µM SER (250 µL ×3)	30	75
**7**	300	30	250 µM PRO (250 µL ×3), 250 µM LIDO (250 µL ×3), 250 µM PMZ (250 µL ×3), 250 µM CAF + QUI(250 µL ×3), 250 µM DPH (250 µL ×3), 250 µM FEN (250 µL ×6), 250 µM COC (250 µL ×3), 250 µM DOPAC + HVA (250 µL ×3), 250 µM MPD (250 µL ×3), 50 µM ALP (250 µl ×3), 250 µM PSU (250 µL ×3), 250 µM NE + EPI (250 µL ×3), 250 µM BPP (250 µL ×3), 250 µM METD + MOR (250 µL ×3), 250 µM MDPV + MDMA (250 µL ×3),250 µM AMP (250 µL ×3), 250 µM DOP (250 µL ×3), 250 µM 4-HMA (250 µL ×3), 250 µM TYM (250 µL ×3), 250 µM 4-HA (250 µL ×3), 250 µM SER (250 µL ×3), 250 µM TYR + PHE (250 µL ×3)	30	50
**8**	300	30	250 µM PRO (250 µL ×3), 250 µM LIDO (250 µL ×3), 250 µM PMZ (250 µL ×3), 250 µM CAF + QUI(250 µL ×3), 250 µM DPH (250 µL ×3), 250 µM FEN (250 µL ×6), 250 µM COC (250 µL ×3), 250 µM DOPAC + HVA (250 µL ×3), 250 µM MPD (250 µL ×3), 50 µM ALP (250 µl ×3), 250 µM PSU (250 µL ×3), 250 µM NE + EPI (250 µL ×3), 250 µM BPP (250 µL ×6), 250 µM METD + MOR (250 µL ×3), 250 µM MDPV + MDMA (250 µL ×3),250 µM AMP (250 µL ×3), 250 µM DOP (250 µL ×3), 250 µM 4-HMA (250 µL ×3), 250 µM TYM (250 µL ×3), 250 µM 4-HA (250 µL ×3), 250 µM SER (250 µL ×3), 250 µM TYR + PHE (250 µL ×3)	30	50
**9**	300	30	Same as previous round	30	50
**10**	300	30	Same as previous round	30	50
**11**	300	30	250 µM PRO (250 µL ×3), 250 µM LIDO (250 µL ×3), 250 µM PMZ (250 µL ×3), 250 µM CAF + QUI(250 µL ×3), 250 µM DPH (250 µL ×3), 250 µM AMP (250 µL ×3), 250 µM COC (250 µL ×3), 250 µM DOPAC + HVA (250 µL ×3), 250 µM MPD (250 µL ×3), 50 µM ALP (250 µl ×3), 250 µM PSU (250 µL ×3), 250 µM NE + EPI (250 µL ×3), 250 µM BPP (250 µL ×6), 250 µM METD + MOR (250	30	50

			µL ×3), 250 µM MDPV + MDMA (250 µL ×3), 250 µM FEN (250 µL ×6), 250 µM DOP (250 µL ×3), 250 µM 4-HMA (250 µL ×3), 250 µM TYM (250 µL ×3), 250 µM 4-HA (250 µL ×3), 250 µM SER (250 µL ×3), 250 µM TYR + PHE (250 µL ×3)		
**12**	300	30	Same as previous round	30	50
**13**	300	30	Same as previous round	30	50
**14**	300	30	Same as previous round	30	50
**15**	300	30	Same as previous round	30	50
**16**	300	30	Same as previous round	30	50
**17**	300	30	250 µM PRO (250 µL ×3), 250 µM LIDO (250 µL ×3), 250 µM PMZ (250 µL ×3), 250 µM CAF + QUI(250 µL ×3), 250 µM DPH (250 µL ×3), 250 µM AMP (250 µL ×3), 250 µM COC (250 µL ×3), 250 µM DOPAC + HVA (250 µL ×3), 250 µM MPD (250 µL ×3), 50 µM ALP (250 µl ×3), 250 µM PSU (250 µL ×3), 250 µM NE + EPI (250 µL ×3), 250 µM BPP (250 µL ×6), 250 µM METD + MOR (250 µL ×3), 250 µM MDPV + MDMA (250 µL ×3), 250 µM DOP (250 µL ×3), 250 µM 4-HMA (250 µL ×3), 250 µM TYM (250 µL ×3), 250 µM 4-HA (250 µL ×3), 250 µM SER (250 µL ×3), 250 µM TYR + PHE (250 µL ×3)	30	50
**18**	300	30	Same as previous round	30	50
**19**	300	30	250 µM PRO (250 µL ×3), 250 µM LIDO (250 µL ×3), 250 µM CAF + QUI(250 µL ×3), 250 µM DPH (250 µL ×3), 250 µM AMP (250 µL ×3), 250 µM COC (250 µL ×3), 250 µM DOPAC + HVA (250 µL ×3), 250 µM MPD (250 µL ×3), 50 µM ALP (250 µl ×3), 250 µM PSU (250 µL ×3), 250 µM NE + EPI (250 µL ×3), 250 µM BPP (250 µL ×6), 250 µM METD + MOR (250 µL ×3), 250 µM MDPV + MDMA (250 µL ×3), 250 µM DOP (250 µL ×3), 250 µM 4-HMA (250 µL ×3), 250 µM TYM (250 µL ×3), 250 µM 4-HA (250 µL ×3), 250 µM SER (250 µL ×3), 250 µM TYR + PHE (250 µL ×3)	30	50

PRO (Procaine), LIDO (Lidocaine), CAF (Caffeine), QUI (Quinine), DPH (Diphenhydramine), AMP (Amphetamine), COC (Cocaine), DOPAC (3,4-Dihydroxyphenylacetic acid), HVA (Homovanillic acid), MPD (Methylphenidate), ALP (Alprazolam), PSU ((+)-Pseudoephedrine), NE (Norepinephrine), EPI (Epinephrine), BPP (Bupropion), METD (Methadone), MOR (Morphine), MDPV (3,4-Methylenedioxypyrovalerone), MDMA (3,4-Methylenedioxymethamphetamine), FEN (Fentanyl), DOP (Dopamine), 4-HMA (4-Hydroxymethamphetamine), TYM (Tyramine), 4-HA (4-Hydroxyamphetamine), SER (Serotonin), TYR (Tyrosine), PHE (Phenylalanine), METH ((+)-Methamphetamine).

Table S3. Selection strategy and conditions for the second trial of methamphetamine SELEX.

Round	Pool Size (pmol)	Buffer Wash (×)	Counter-targets	Buffer Wash (×)	[Target] (µM)
**1**	1,000	10	None	None	500
**2**	300	20	None	None	250
**3**	300	30	None	None	250
**4**	300	40	None	None	250
**5**	300	50	None	None	250
**6**	300	50	None	None	250
**7**	300	50	None	None	250
**8**	300	50	None	None	250
**9**	300	50	None	None	250
**10**	300	50	None	None	250
**11**	300	50	None	None	250

Table S4. Selection strategy and conditions for the third trial of methamphetamine SELEX.

Round	Pool Size (pmol)	Buffer Wash (×)	Counter-SELEX targets	Buffer Wash (×)	[Target] (µM)
**1**	1,000	10	None	None	500
**2**	300	20	100 µM PRO (250 µL ×3), 100 µM LIDO (250 µL ×3), 100 µM CAF (250 µL ×3), 100 µM QUI (250 µL ×3), 100 µM DPH (250 µL ×3), 100 µM AMP (250 µL ×3)	20	250
**3**	300	20	100 µM PRO (250 µL ×3), 100 µM LIDO (250 µL ×3), 100 µM CAF (250 µL ×3), 100 µM QUI (250 µL ×3), 100 µM DPH (250 µL ×3), 100 µM AMP (250 µL ×3), 100 µM DOP (250 µL ×3), 100 µM TYM (250 µL ×3), 100 µM 4-HA (250 µL ×3), 100 µM SER (250 µL ×3), 100 µM DOPAC (250 µL ×3), 100 µM HVA (250 µL ×3), 100 µM EPI (250 µL ×3)	30	250
**4**	300	20	250 µM PRO (250 µL ×3), 250 µM LIDO (250 µL ×3), 250 µM CAF (250 µL ×3), 250 µM QUI (250 µL ×3), 250 µM DPH (250 µL ×3), 250 µM AMP (250 µL ×3), 100 µM DOP (250 µL ×3), 100 µM 4-HMA (250 µL ×3), 100 µM TYM (250 µL ×3), 100 µM 4-HA (250 µL ×3), 100 µM SER (250 µL ×3), 100 µM DOPAC (250 µL ×3), 100 µM HVA (250 µL ×3), 100 µM MPD (250 µL ×3), 100 µM ALP (250 µL ×3), 100 µM PSU (250 µL ×3), 100 µM NE (250 µL ×3), 100 µM EPI (250 µL ×3), 100 µM BPP (250 µL ×3)	30	100
**5**	300	24	250 µM PRO (250 µL ×3), 250 µM LIDO (250 µL ×3), 250 µM CAF (250 µL ×3), 250 µM QUI (250 µL ×3), 250 µM DPH (250 µL ×3), 250 µM AMP (250 µL ×3), 250 µM DOP (250 µL ×3), 250 µM 4-HMA (250 µL ×3), 250 µM TYM (250 µL ×3), 250 µM 4-HA (250 µL ×3), 250 µM SER (250 µL ×3), 250 µM DOPAC (250 µL ×3), 250 µM HVA (250 µL ×3), 100 µM MPD (250 µL ×3), 100 µM ALP (250 µL ×3), 100 µM PSU (250 µL ×3), 100 µM NE (250 µL ×3), 100 µM EPI (250 µL ×3), 100 µM BPP (250 µL ×3), 100 µM METD (250 µL ×3), 100 µM MOR (250 µL ×3), 100 µM MDPV (250 µL ×3), 100 µM MDMA (250 µL ×3), 100 µM TYR + PHE (250 µL ×3)	30	100
**6**	300	30	250 µM PRO (250 µL ×3), 250 µM LIDO (250 µL ×3), 250 µM CAF (250 µL ×3), 250 µM QUI (250 µL ×3), 250 µM DPH (250 µL ×3), 250 µM AMP (250 µL ×3), 250 µM DOP (250 µL ×3), 250 µM 4-HMA (250 µL ×3), 250 µM TYM (250 µL ×3), 250 µM 4-HA (250 µL ×3), 250 µM SER (250 µL ×3), 250 µM DOPAC (250 µL ×3), 250 µM HVA (250 µL ×3), 250 µM MPD (250 µL ×3), 100 µM ALP (250 µL ×3), 250 µM PSU (250 µL ×3), 250 µM NE (250 µL ×3), 250 µM EPI (250 µL ×3), 250 µM BPP (250 µL ×3), 250 µM METD (250 µL ×3), 250 µM MOR (250 µL ×3), 250 µM MDPV (250 µL ×3), 250 µM MDMA (250 µL ×3), 250 µM TYR + PHE (250 µL ×3)	30	100
**7**	300	30	250 µM PRO (250 µL ×3), 250 µM LIDO (250 µL ×3), 250 µM CAF (250 µL ×3), 250 µM QUI (250 µL ×3), 250 µM DPH (250 µL ×3), 250 µM AMP (250 µL ×3), 250 µM DOP (250 µL ×3), 250 µM 4-HMA (250 µL ×3), 250 µM TYM (250 µL ×3), 250 µM 4-HA (250 µL ×3), 250 µM SER (250 µL ×3), 250 µM DOPAC (250 µL ×3), 250 µM HVA (250 µL ×3), 250 µM MPD (250 µL ×3), 100 µM ALP (250 µL ×3), 250 µM PSU (250 µL ×3), 250 µM NE (250 µL ×3), 250 µM EPI (250 µL ×3), 250 µM BPP (250 µL ×6), 250 µM METD (250 µL ×3), 250 µM MOR (250 µL ×3), 250 µM MDPV (250 µL ×3), 250 µM MDMA (250 µL ×3), 250 µM TYR + PHE (250 µL ×3)	30	100
**8**	300	30	250 µM PRO (250 µL ×3), 250 µM LIDO (250 µL ×3), 250 µM CAF (250 µL ×3), 250 µM QUI (250 µL ×3), 250 µM DPH (250 µL ×3), 250 µM AMP (250 µL ×9), 250 µM DOP (250 µL ×3), 250 µM 4-HMA (250 µL ×12), 250 µM TYM (250 µL ×9), 250 µM 4-HA (250 µL ×9), 250 µM SER (250 µL ×3), 250 µM DOPAC (250 µL ×3), 250 µM HVA (250 µL ×3), 250 µM MPD (250 µL ×3), 100 µM ALP (250 µL ×3), 250 µM PSU (250 µL ×3), 250 µM NE (250 µL ×9), 250 µM EPI (250 µL ×6), 250 µM BPP (250 µL ×12), 250 µM METD (250 µL ×3), 250 µM MOR (250 µL ×3), 250 µM MDPV (250 µL ×3), 250 µM MDMA (250 µL ×12), 250 µM TYR + PHE (250 µL ×3)	30	100
**9**	300	30	Same as previous round	30	100
**10**	300	30	Same as previous round	30	100
**11**	300	30	Same as previous round	30	100

**12**	300	30	Same as previous round	30	100
**13**	300	30	Same as previous round	30	100
**14**	300	30	Same as previous round	30	100
**15**	300	30	250 µM PRO (250 µL ×3), 250 µM LIDO (250 µL ×3), 250 µM CAF (250 µL ×3), 250 µM QUI (250 µL ×3), 250 µM DPH (250 µL ×3), 250 µM AMP (250 µL ×12), 250 µM DOP (250 µL ×3), 250 µM 4-HMA (250 µL ×12), 250 µM TYM (250 µL ×9), 250 µM 4-HA (250 µL ×9), 250 µM SER (250 µL ×3), 250 µM DOPAC (250 µL ×3), 250 µM HVA (250 µL ×3), 250 µM MPD (250 µL ×3), 100 µM ALP (250 µL ×3), 250 µM PSU (250 µL ×3), 250 µM NE (250 µL ×9), 250 µM EPI (250 µL ×6), 250 µM BPP (250 µL ×12), 250 µM METD (250 µL ×3), 250 µM MOR (250 µL ×3), 250 µM MDPV (250 µL ×3), 250 µM MDMA (250 µL ×16), 250 µM TYR + PHE (250 µL ×3)	30	100
**16**	300	30	250 µM PRO (250 µL ×3), 250 µM LIDO (250 µL ×3), 250 µM CAF (250 µL ×3), 250 µM QUI (250 µL ×3), 250 µM DPH (250 µL ×3), 250 µM AMP (250 µL ×16), 250 µM DOP (250 µL ×3), 250 µM 4-HMA (250 µL ×12), 250 µM TYM (250 µL ×9), 250 µM 4-HA (250 µL ×9), 250 µM SER (250 µL ×3), 250 µM DOPAC (250 µL ×3), 250 µM HVA (250 µL ×3), 250 µM MPD (250 µL ×3), 100 µM ALP (250 µL ×3), 250 µM PSU (250 µL ×3), 250 µM NE (250 µL ×9), 250 µM EPI (250 µL ×6), 250 µM BPP (250 µL ×12), 250 µM METD (250 µL ×3), 250 µM MOR (250 µL ×3), 250 µM MDPV (250 µL ×3), 250 µM MDMA (250 µL ×16), 250 µM TYR + PHE (250 µL ×3)	30	100
**17**	300	30	Same as previous round	30	100
**18**	300	30	Same as previous round	30	100

PRO (Procaine), LIDO (Lidocaine), CAF (Caffeine), QUI (Quinine), DPH (Diphenhydramine), AMP (Amphetamine), DOPAC (3,4-Dihydroxyphenylacetic acid), HVA (Homovanillic acid), MPD (Methylphenidate), ALP (Alprazolam), PSU ((+)-Pseudoephedrine), NE (Norepinephrine), EPI (Epinephrine), BPP (Bupropion), METD (Methadone), MOR (Morphine), MDPV (3,4-Methylenedioxypyrovalerone), MDMA (3,4-Methylenedioxymethamphetamine), DOP (Dopamine), 4-HMA (4-Hydroxy methamphetamine), TYM (Tyramine), 4-HA (4-Hydroxy amphetamine), SER (Serotonin), TYR (Tyrosine), PHE (Phenylalanine), METH ((+)-Methamphetamine).

Table S5. Selection strategy and conditions for the fourth trial of methamphetamine SELEX.

Round	Pool Size (pmol)	Buffer Wash (×)	Counter-SELEX targets	Buffer Wash (×)	Last 3 Washes	[Target] (μM)
**1**	1,000	9	None	None	w/o Triton	500
**2**	300	20	100 μM PRO (250 μL ×3), 100 μM LIDO (250 μL ×3), 100 μM CAF (250 μL ×3), 100 μM QUI (250 μL ×3), 100 μM DPH (250 μL ×3), 100 μM AMP (250 μL ×6), 100 μM BPP (250 μL ×6), 100 μM MDMA (250 μL ×6)	17		250
**3**	300	20	100 μM PRO (250 μL ×3), 100 μM LIDO (250 μL ×3), 100 μM CAF (250 μL ×3), 100 μM QUI (250 μL ×3), 100 μM DPH (250 μL ×3), 100 μM AMP (250 μL ×6), 100 μM BPP (250 μL ×6), 100 μM MDMA (250 μL ×6), 100 μM 4-HMA (250 μL ×6), 100 μM NE (250 μL ×6), 100 μM EPI (250 μL ×6), 100 μM DOP (250 μL ×3), 100 μM TYM (250 μL ×3), 100 μM 4-HA (250 μL ×3), 100 μM TYR (250 μL ×3)	27		250
**4**	300	20	250 μM PRO (250 μL ×3), 250 μM LIDO (250 μL ×3), 250 μM CAF (250 μL ×3), 100 μM QUI (250 μL ×3), 250 μM DPH (250 μL ×3), 100 μM AMP (250 μL ×6), 100 μM BPP (250 μL ×6), 100 μM MDMA (250 μL ×6), 100 μM 4-HMA (250 μL ×6), 100 μM NE (250 μL ×6), 100 μM EPI (250 μL ×6), 100 μM DOP (250 μL ×3), 100 μM TYM (250 μL ×3), 100 μM 4-HA (250 μL ×3), 100 μM TYR (250 μL ×3), 100 μM MPD (250 μL ×3), 100 μM ALP (250 μL ×3), 100 μM PSU (250 μL ×3), 100 μM METD (250 μL ×3), 100 μM MOR (250 μL ×3), 100 μM MDPV (250 μL ×3), 100 μM PHE (250 μL ×3), 100 μM SER (250 μL ×3), 100 μM DOPAC (250 μL ×3), 100 μM HVA (250 μL ×3)	27		250
**5**	300	24	250 μM PRO (250 μL ×3), 250 μM LIDO (250 μL ×3), 250 μM CAF (250 μL ×3), 100 μM QUI (250 μL ×3), 250 μM DPH (250 μL ×3), 250 μM AMP (250 μL ×6), 250 μM BPP (250 μL ×6), 250 μM MDMA (250 μL ×6), 250 μM 4-HMA (250 μL ×6), 250 μM NE (250 μL ×6), 250 μM EPI (250 μL ×6), 250 μM DOP (250 μL ×3), 250 μM TYM (250 μL ×3), 250 μM 4-HA (250 μL ×3), 250 μM TYR (250 μL ×3), 250 μM MPD (250 μL ×3), 100 μM ALP (250 μL ×3), 250 μM PSU (250 μL ×3), 250 μM METD (250 μL ×3), 250 μM MOR (250 μL ×3), 250 μM MDPV (250 μL ×3), 250 μM PHE (250 μL ×3), 250 μM SER (250 μL ×3), 250 μM DOPAC (250 μL ×3), 250 μM HVA (250 μL ×3)	27		100
**6**	300	30	Same as previous round	27		100
**7**	300	30	Same as previous round	27		100
**8**	300	30	Same as previous round	27		100
**9**	300	30	Same as previous round	27		100
**10**	300	30	Same as previous round	27		100
**11**	300	30	Same as previous round	27		100
**12**	300	30	Same as previous round	27		100
**13**	300	30	Same as previous round	27		100

PRO (Procaine), LIDO (Lidocaine), CAF (Caffeine), QUI (Quinine), DPH (Diphenhydramine), AMP (Amphetamine), DOPAC (3,4-Dihydroxyphenylacetic acid), HVA (Homovanillic acid), MPD (Methylphenidate), ALP (Alprazolam), PSU ((+)-Pseudoephedrine), NE (Norepinephrine), EPI (Epinephrine), BPP (Bupropion), METD (Methadone), MOR (Morphine), MDPV (3,4-Methylenedioxypyrovalerone), MDMA (3,4-Methylenedioxymethamphetamine), DOP (Dopamine), 4-HMA (4-Hydroxy methamphetamine), TYM (Tyramine), 4-HA (4-Hydroxy amphetamine), SER (Serotonin), TYR (Tyrosine), PHE (Phenylalanine), METH ((+)-Methamphetamine).

Table S6. Summary HTS statistics for methamphetamine-binding aptamer selection from Trials 1–4.

Pool	Total Reads	Filtered Reads	Unique Sequences	Unique Sequences (%)
**SELEX Trial 1**				
Round 13	462,902	436,550	189,869	43.5
Round 19	466,542	441,260	189,018	42.8
**SELEX Trial 2**				
Round 9	533,226	477,697	185,314	38.8
Round 11	449,060	384,057	82,997	21.6
**SELEX Trial 3**				
Round 7	382,954	366,805	170,657	46.5
Round 13	1,345,800	1,236,714	381,431	30.8
Round 18	1,269,964	1,159,331	216,220	18.7
**SELEX Trial 4**				
Round 9	431,950	392,633	172,750	44.0
Round 10	498,324	447,146	183,069	40.9
Round 11	504,238	460,649	147,713	32.1
Round 13	515,898	476,413	45,953	9.6
Round 14	407,918	389,749	28,394	7.3
Round 15	409,406	396,736	17,791	4.5

Table S7. Summary of ITC experimental conditions and measurements for methamphetamine aptamer sequences.

Name	Titration time	ΔH (kcal/mol)	ΔS (cal/mol·K)	K_D (μM)	Titration time	ΔH (kcal/mol)	ΔS (cal/mol·K)	K_D (μM)
	(+)-Methamphetamine				Amphetamine			
**SELEX Trial 1**								
MT1-R1	1×	N/A	N/A	N/A	-	-	-	-
MT1-R2	1×	N/A	N/A	N/A	-	-	-	-
MT1-R3	1×	N/A	N/A	N/A	-	-	-	-
MT1-R4	1×	N/A	N/A	N/A	-	-	-	-
MT1-R5	1×	N/A	N/A	N/A	-	-	-	-
**SELEX Trial 2**								
MT2-R1	1×	-14.9	-24.7	2.5 ± 0.1	1×	-14.8	-25.9	5.7 ± 0.2
MT2-R2	1×	-20.9	-46.2	5.0 ± 0.1	-	-	-	-
MT2-R3	1×	-6.8	-1.3	18.0 ± 1.1	-	-	-	-
MT2-R4	1×	-6.1	2.3	10.1 ± 0.7	-	-	-	-
**SELEX Trial 3**								
M1	1×	-10.3	-10.7	5.1 ± 0.5	1×	-12.4	-18.8	9.1 ± 0.5
M2	1×	-9.9	-8.5	3.6 ± 0.3	1×	-11.1	-13.6	6.6 ± 0.4
M3	1×	-12.5	-17.9	4.9 ± 0.5	1×	-4.8	2.5	78.5 ± 4.7
M4	1×	-9.2	-8.9	12.4 ± 0.8	1×	-9.0	-9.1	22.9 ± 1.5
M5	1×	-6.2	3.2	5.3 ± 0.5	1×	-2.5	10.9	56.4 ± 3.2
M6	1×	-12.1	-16.4	4.4 ± 0.2	1×	-11.8	-15.9	6.4 ± 0.2
M7	1×	-8.6	-5.7	6.0 ± 0.5	1×	-4.1	7.0	27.2 ± 1.1
M8	1×	-6.5	1.3	8.5 ± 0.8	1×	-3.7	9.0	19.3 ± 1.5
M9	1×	-8.3	-4.1	6.1 ± 0.4	1×	-7.1	-5.6	89.1 ± 6.2
M11	1×	-20.7	-46.7	8.9 ± 0.1	2×	-6.9	-5.7	127 ± 6.8
M12	1×	-14.3	-24.8	7.5 ± 0.2	2×	-19.2	-47.3	171 ± 16.1
M13	1×	-10.1	-8.9	3.3 ± 0.1	1×	-4.6	5.9	20.0 ± 0.9
M14	1×	-14.5	-24.1	3.7 ± 0.2	1×	-12.9	-20.0	7.6 ± 0.5
M18	1×	-7.0	-0.1	7.0 ± 0.6	1×	-3.1	8.9	60.3 ± 3.9
M20	1×	-6.8	0.7	6.4 ± 0.3	2×	-2.9	9.6	60.6 ± 2.9
M21	1×	-6.7	0.8	6.9 ± 0.3	2×	-3.5	7.1	70.8 ± 3.1
**SELEX Trial 4**								
ML1	1×	-42.8	-119.2	3.3 ± 0.1	1×	-27.4	-73.6	67.2 ± 1.1
ML2	1×	-31.4	-81.4	4.4 ± 0.2	1×	-15.2	-31.5	45.3 ± 1.3
ML3	1×	-12.7	-16.2	1.5 ± 0.1	2×	-4.8	2.4	80.6 ± 3.1
ML4	1×	-19.2	-39.2	2.5 ± 0.1	2×	-9.1	-12.7	117 ± 4.1
ML5	1×	- 13.1	-17.8	1.6 ± 0.1	1×	-10.9	-12.7	5.25 ± 0.3
ML6	1×	-41.6	-114.5	2.1 ± 0.1	1×	-25.0	-64.1	40.5 ± 0.6
ML7	1×	-24.6	-56.0	1.3 ± 0.1	1×	-12.7	-21.1	18.0 ± 0.6
ML8	1×	-9.8	-9.8	8.0 ± 0.4	1×	-6.7	-2.7	43.7 ± 1.8
ML10	1×	-40.0	-109.0	1.9 ± 0.1	1×	-25.0	-64.5	43.0 ± 1.6
ML11	1×	-43.0	-119.5	2.4 ± 0.1	1×	-25.6	-66.5	46.6 ± 0.7
ML12	1×	-7.2	-0.3	5.7 ± 0.3	1×	-8.4	-6.7	18.2 ± 0.5
ML13	1×	-34.5	-92.9	7.0 ± 0.4	2×	-24.5	-65.2	140 ± 3.9

Table S8. ITC results and conditions for determining affinity of methamphetamine aptamer sequences.

Aptamer	[Aptamer] (μM)	[(+)-METH] (μM)	ΔH (kcal/mol)	ΔS (cal/mol·K)	K_D (μM)	Buffer
ML3	50	1500	-20.0	-47	34.3 ± 1.9	0.5× PBS, 1 mM $MgCl_2$, 0.005% Triton X-100
	50	1250	-13.5	-23	9.9 ± 0.6	0.5× PBS, 2 mM $MgCl_2$, 0.005% Triton X-100
	20	500	-12.7	-16	1.5 ± 0.1	0.5× PBS, 5 mM $MgCl_2$, 0.005% Triton X-100
ML4	50	1500	-47.4	-141	65.2 ± 1.8	0.5× PBS, 1 mM $MgCl_2$, 0.005% Triton X-100
	50	1250	-22.2	-53	16.2 ± 0.5	0.5× PBS, 2 mM $MgCl_2$, 0.005% Triton X-100
	20	500	-19.2	-39	2.5 ± 0.1	0.5× PBS, 5 mM $MgCl_2$, 0.005% Triton X-100
ML3-mut1	50	2000	-4.4	0.5	408 ± 16	0.5× PBS, 5 mM $MgCl_2$, 0.005% Triton X-100
ML3-mut2	50	2000	-5.4	2.1	39.0 ± 1.8	
ML3-mut3	50	2000	-10.9	-10	1.4 ± 0.1	
ML3-mut4	50	2000	-5.1	1.6	72.5 ± 3.1	
ML3-mut5	50	2000	NA	NA	NA	
ML3-mut6	50	2000	NA	NA	NA	
ML4-mut1	50	2000	-9.9	-14	67.1 ± 4.1	0.5× PBS, 5 mM $MgCl_2$, 0.005% Triton X-100
ML4-mut2	50	2000	-12.1	-16	4.0 ± 0.1	
ML4-mut3	50	2000	-15.2	-26	3.2 ± 0.1	
ML4-mut4	50	2000	-16.2	-30	4.9 ± 0.2	
ML4-mut5	50	2000	-16.5	-31	3.3 ± 0.1	
ML4-mut6	50	2000	-14.6	-24	3.8 ± 0.1	
ML4-mut7	50	2000	-8.4	-9	64.3 ± 3.9	

Table S9. List of methamphetamine aptamer sequences used in this work.

**Aptamer Name**	**Sequence (5' – 3')**
84-nt aptaMETH	ACTGGAGCTCAATCAGTACACGACGGTTGCAAGTGGGACTCTGGTAGGCTGGGTTAATTTGGGACAAGCT TCAACCATGGAGTA
38-nt aptaMETH	ACGGTTGCAAGTGGGACTCTGGTAGGCTGGGTTAATTTGG
Aptamer-2	AGGAATTCAGATCTCCCTGCAGGTGGTGTTTTTTTGTGTGCTGTGTTTTTGTGATGCATGCTCGAGGAGC TCAGGATCCCG
Apta-4	ATACGAGCTTGTTCAATAGCGTTTCTATCTGGCTGTATCGTGATAGTAAGAGCACTAATGATAGTAAGAGCA ATC
	**SELEX Trial 1**
MT1-R1	GGGACGACCCCATTAGTGGTGCCGGGTGTTTATGGTGCGTCGTCCC
MT1-R2	GGGACGACCACTTTGGGCAGGTAGGTTGGGCAAGTAGTGTCGTCCC
MT1-R3	GGGACGACCCAGTTGGGTAGGTATGGTTGGGCACGGTGGTCGTCCC
MT1-R4	GGGACGACGGCACGGTTAGCATGGCTGTATTTGTGTAGGTCGTCCC
MT1-R5	GGGACGACCACGGTTAAAGTAGCACTTTGTATTTGGGTGTCGTCCC
	**SELEX Trial 2**
MT2-R1	GGGACGACGCTAGGGGATCTTTTGGGGTTTGTATGGGCGTCGTCCC
MT2-R2	GGGACGACGAGGGGATCCATCCAATGGGGTTTGTATACGTCGTCCC
MT2-R3	GGGACGACGGTACCCGCCGGTGCAGGATCTGGGGTGGTGTCGTCCC
MT2-R4	GGGACGACGGCAATTGCCGGTGCAGGATCTGAATTATCGTCGTCCC
	**SELEX Trial 3**
M1	GGGACGACGGGATGGTAAGTGGTAGGTCGTTGGAATTTCTACTGTATCGTCGTCCC
M2	GGGACGACGGGACGGTAAGTGGTAGGTCGTTGGAATTTCTACTGTATCGTCGTCCC
M3	GGGACGACCGTCCGAGTTTTAGGAGTGGTTGCATTGCGCTGGGAAGAGGTCGTCCC
M4	GGGACGACGCACTATGGCTAGGGTTGGTACGGTCGTGAACCGCGGGGTGTCGTCCC
M5	GGGACGACGGCACGGGGTTATTCGTTGGTTGCACTGCGCTGGGAGGCAGTCGTCCC
M6	GGGACGACGGGATGGTAAGTGGCAGGTCGTTGGAATTTCTACTGTATCGTCGTCCC
M7	GGGACGACGGCACGAGGTTATTCGTTGGTTGCACTGCGCTGGGAGGTAGTCGTCCC
M8	GGGACGACGGGGCTCTTACCCTGGAGGGTAGAAGGGGAGGTGTGGTCAGTCGTCCC
M9	GGGACGACGGCACGTGGTTATTCGTTGGTTGCACTGCGCTGGGAGGCAGTCGTCCC
M11	GGGACGACCGGCTGTCTGGATGCATTGCGCCGGGAACTCGGACGGATGGTCGTCCC
M12	GGGACGACCGTCCGAGTTATAGGAGTGGTTGCATTGCGCTGGGAAGAGGTCGTCCC
M13	GGGACGACGGCACGAGGTTATTCGTTGGTTGCACTGCGCTGGGAGGAAGTCGTCCC
M14	GGGACGACGGGACGGTAAGTGGCAGGTCGTTGGAATTTCTACTGTATCGTCGTCCC
M18	GGGACGACGGCACGAGGTTATTCGTTGGTTGCACTGCGCTGGGAGGCAGTCGTCCC
M20	GGGACGACGGCACGAGGTTAATCGTTGGTTGCACTGCGCTGGGAGGCAGTCGTCCC
M21	GGGACGACGGCACGAGGTAATTCGTTGGTTGCACTGCGCTGGGAGGCAGTCGTCCC
	**SELEX Trial 4**
ML1	TTCAAATCTGAGCAGCCTAGTTACTGACGGGTTTATAGTACTCGCATTGAGTCTAGATTTGAA
ML2	TTCAAATCTGGTGCCCTTTCGCAGTCGGTTGGTAATGAGACCACTTTAGAGTCTAGATTTGAA
ML3	TTCAAATCTTATGTACGTTCTATCGCATGGATGGGATTTTGGGTTACCTAGTCTAGATTTGAA
ML4	TTCAAATCTTGGTACGTTCTGCCTTTTCTTATTTTGCTTTGGGTTACCTAGTCTAGATTTGAA
ML5	TTCAAATCTATGGTGCAGTGCGCAGGGACGGAGGTAAAACATGAGTTTTAGTCTAGATTTGAA
ML6	TTCAAATCTGAGCAGCCTAGTTACTGACGGGTTTATAGTACTCGCATCGAGTCTAGATTTGAA
ML7	TTCAAATCTGGTGCCCTTTCGCAGTCGGTTGGTAATGAGATCACTTTAGAGTCTAGATTTGAA
ML8	TTCAAATCTCCACGCTGGACTGGATGCAATGCGCCGGGATATCGTCTAAAGTCTAGATTTGAA
ML10	TTCAAATCTGAGCAGCCTAGTTACTGACGGGTTTATAGTACTCACATTGAGTCTAGATTTGAA
ML11	TTCAAATCTGAGCAGCCTAGTTACTGACGGGTTTATAGTACTCGTATTGAGTCTAGATTTGAA
ML12	TTCAAATCTGCGGCGACTAATTCTTAGTGGTGGTTGCAATGCGCTGGGATGAGTCTAGATTTGAA
ML13	TTCAAATCTGAGCAGCCTAGTTACTGACGGGTTTATAGTACTCGCACTGAGTCTAGATTTGAA

Table S10. List of point-mutant sequences of methamphetamine aptamer used in this work.

Name	Sequence (5' – 3')
ML3-mut1	TTCAAATCTTATGTAAGTTCTATCGCATGGATGGGATTTTGGGTTACCTAGTCTAGATTTGAA
ML3-mut2	TTCAAATCTTATGTACGTACTATCGCATGGATGGGATTTTGGGTTACCTAGTCTAGATTTGAA
ML3-mut3	TTCAAATCTTATGTACGTTCTATCGCATGGATGTGATTTTGGGTTACCTAGTCTAGATTTGAA
ML3-mut4	TTCAAATCTTATGTACGTTCTATCGCATGGATGGGATTATGGGTTACCTAGTCTAGATTTGAA
ML3-mut5	TTCAAATCTTATGTACGTTCTATCGCATGGATGGGATTTTTGGTTACCTAGTCTAGATTTGAA
ML3-mut6	TTCAAATCTTATGTACGTTCTATCGCATGGATGGGATTTTGGGTTACCAAGTCTAGATTTGAA
ML4-mut1	TTCAAATCTTGGTACGTACTGCCTTTTCTTATTTTGCTTTGGGTTACCTAGTCTAGATTTGAA
ML4-mut2	TTCAAATCTTGGTACGTTCTGCATTTTCTTATTTTGCTTTGGGTTACCTAGTCTAGATTTGAA
ML4-mut3	TTCAAATCTTGGTACGTTCTGCCTATTCTTATTTTGCTTTGGGTTACCTAGTCTAGATTTGAA
ML4-mut4	TTCAAATCTTGGTACGTTCTGCCTTTACTTATTTTGCTTTGGGTTACCTAGTCTAGATTTGAA
ML4-mut5	TTCAAATCTTGGTACGTTCTGCCTTTTCTTATATTGCTTTGGGTTACCTAGTCTAGATTTGAA
ML4-mut6	TTCAAATCTTGGTACGTTCTGCCTTTTCTTATTTAGCTTTGGGTTACCTAGTCTAGATTTGAA
ML4-mut7	TTCAAATCTTGGTACGTTCTGCCTTTTCTTATTTTGCTATGGGTTACCTAGTCTAGATTTGAA

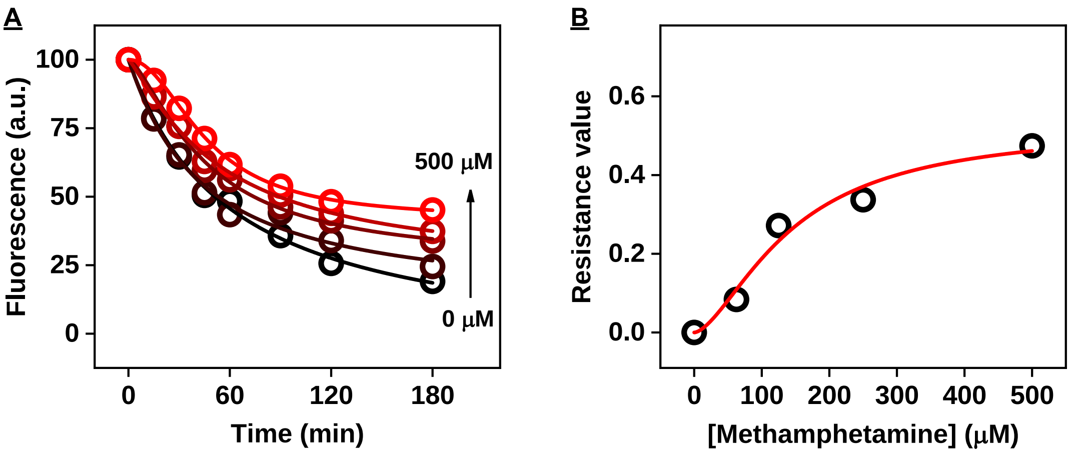

Figure S1. Assessing the binding of aptaMETH to (+)-methamphetamine using the exonuclease digestion assay in their reported selection buffer. (**A**) Time-course of aptaMETH digestion in the absence or presence of various concentrations (0–500 μM) of (+)-methamphetamine. The y-axis represents normalized fluorescence. (**B**) Resistance to exonuclease digestion is plotted as a function of target concentration. The data was fitted with the Hill equation.

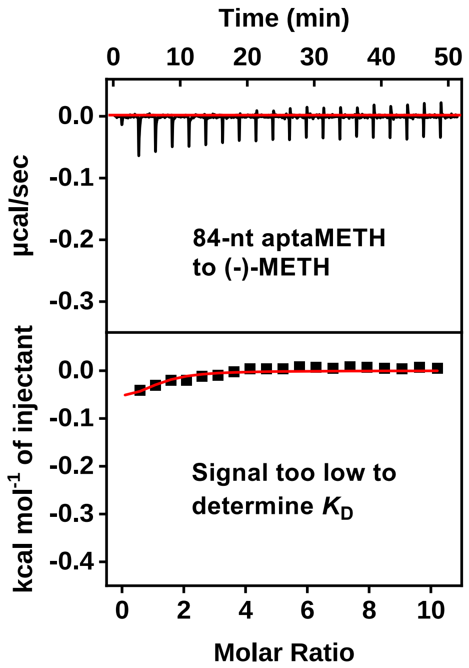

Figure S2. Binding affinity of aptaMETH for (-)-methamphetamine based on isothermal titration calorimetry (ITC) in their reported selection buffer. Top panel displays the heat generated from each titration of (-)-methamphetamine into aptaMETH. Bottom panel shows the integrated heat of each titration after correcting for the heat of dilution of the titrant. While the aptamer shows some degree of binding to this ligand, the signal to noise ratio is too low to quantify this affinity.

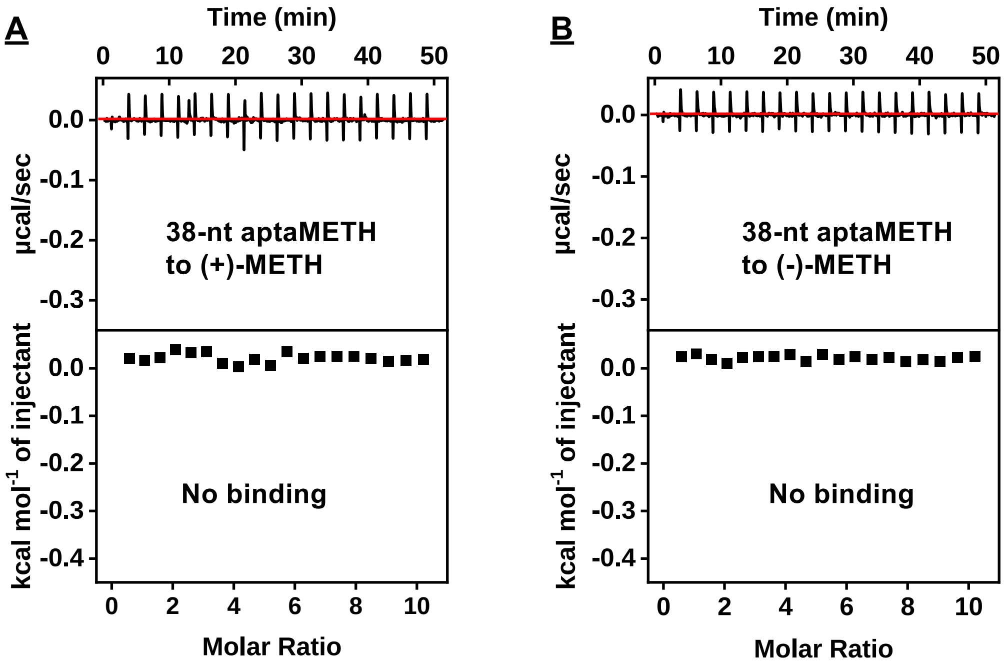

Figure S3. Binding affinity of 38-nt aptaMETH to methamphetamine based on ITC in their reported selection buffer. Top panels display the heat generated from each titration of (**A**) (+)-methamphetamine or (**B**) (-)-methamphetamine into 38-nt aptaMETH. Bottom panels show the integrated heat of each titration after correcting for the heat of dilution of the titrant.

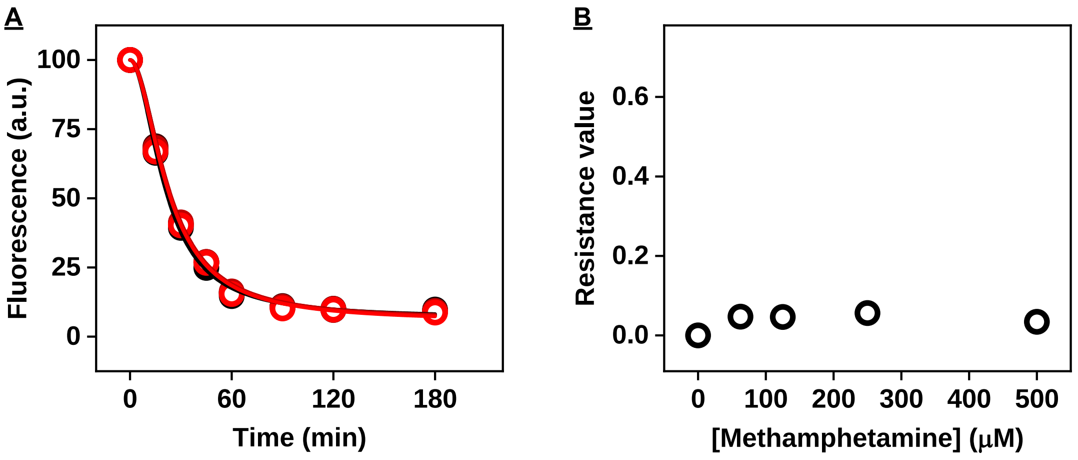

Figure S4. Assessing binding of Aptamer-2 to (+)-methamphetamine using the exonuclease digestion assay in their reported selection buffer. (**A**) Time-course plot of exonuclease digestion of Aptamer-2 in the absence or presence of 0–500 µM (+)-methamphetamine. The y-axis represents normalized fluorescence. (**B**) Resistance to digestion was plotted as a function of target concentration. The data were fitted with the Hill equation.

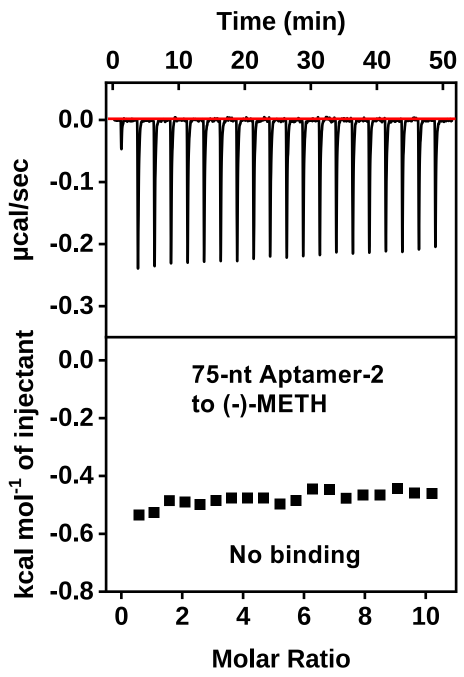

Figure S5. Binding affinity of Aptamer-2 for (-)-methamphetamine based on ITC in their reported selection buffer. Top panel displays the heat generated from each titration of (-)-methamphetamine into Aptamer-2. Bottom panel shows the integrated heat of each titration after correcting for the heat of dilution of the titrant.

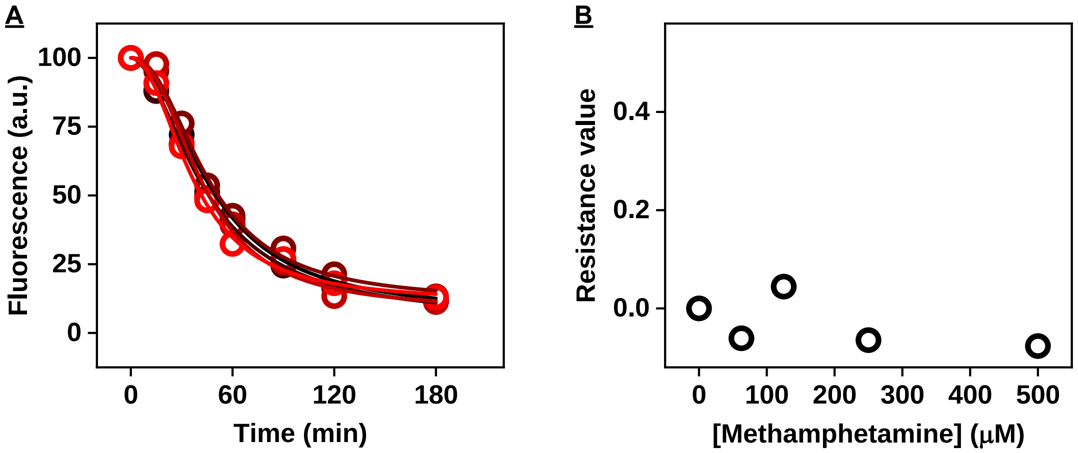

Figure S6. Assessing binding of Apta-4 to (+)-methamphetamine using the exonuclease digestion assay in their reported selection buffer. (**A**) Time-course of exonuclease digestion of Apta-4 in the absence or presence of 0–500 µM of (+)-methamphetamine. The y-axis represents normalized fluorescence. (**B**) Resistance to digestion is plotted as a function of target concentration. The data were fitted with the Hill equation.

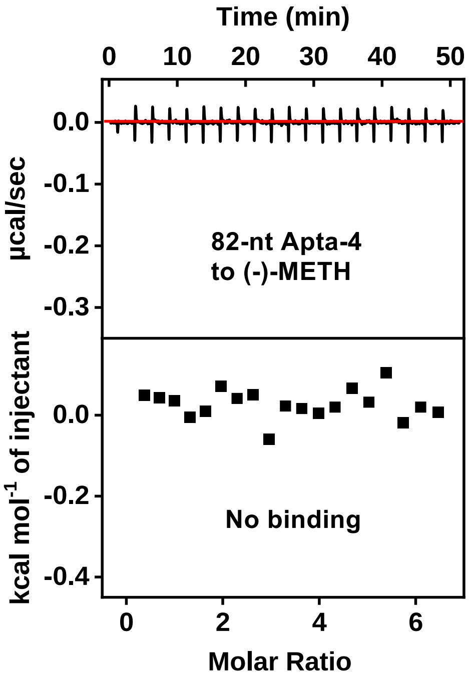

Figure S7. Binding affinity of Apta-4 for (-)-methamphetamine using ITC in their reported selection buffer. Top panel displays the heat generated from each titration of 950 µM (-)-methamphetamine into 30 µM Apta-4. Bottom panel shows the integrated heat of each titration after correcting for the heat of dilution of the titrant.

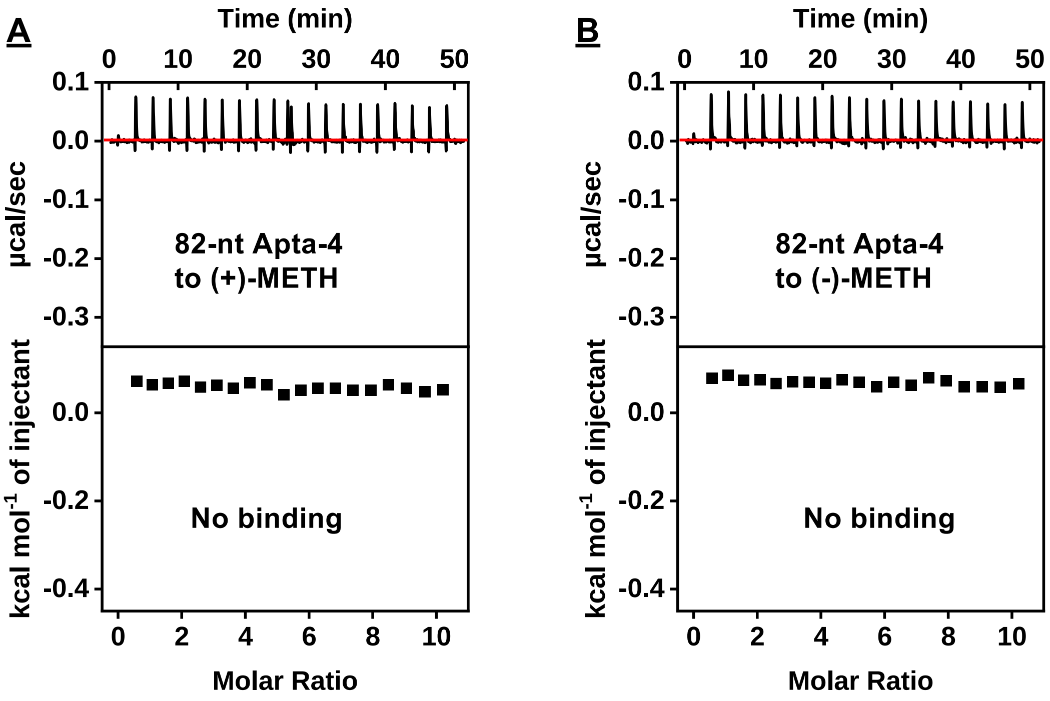

Figure S8. Binding affinity of Apta-4 for methamphetamine based on ITC in their reported selection buffer. Top panels display the heat generated from each titration of (**A**) 5 mM (+)-methamphetamine or (**B**) 5 mM (-)-methamphetamine into 100 µM Apta-4. Bottom panels show the integrated heat of each titration after correcting for the heat of dilution of the titrant.

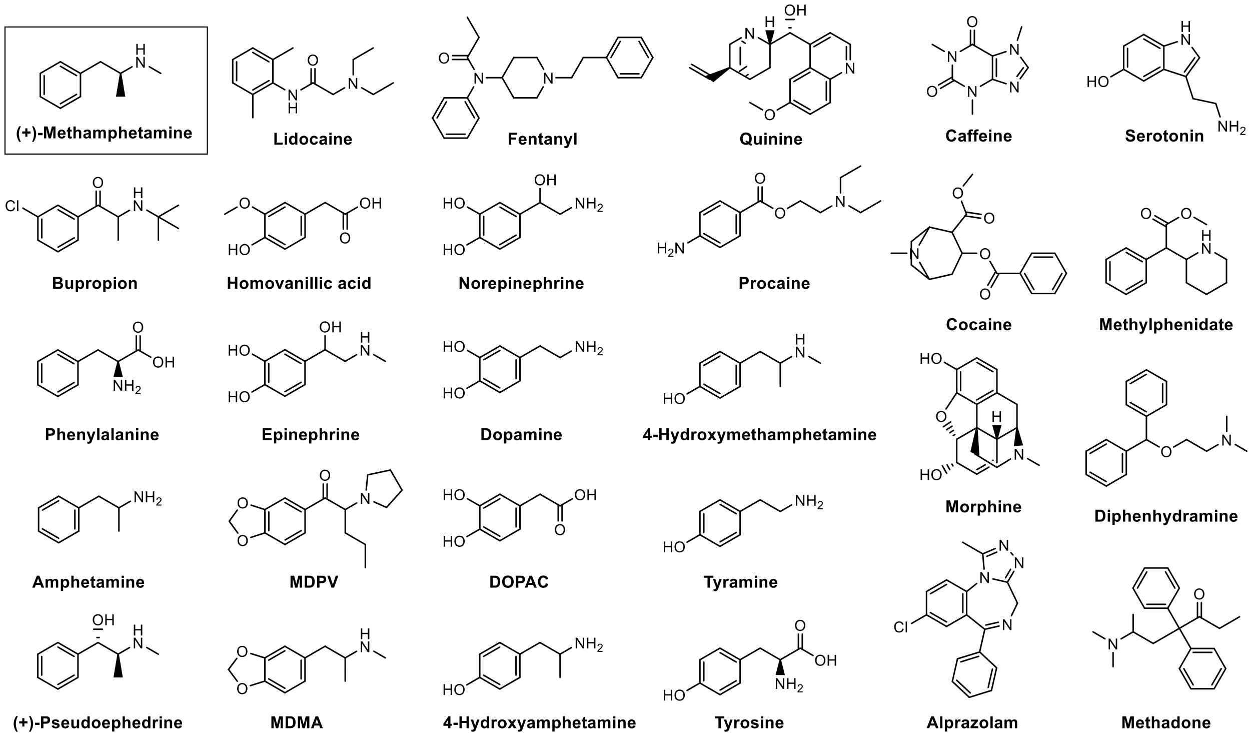

Figure S9. Chemical structures of the compounds used in this work.

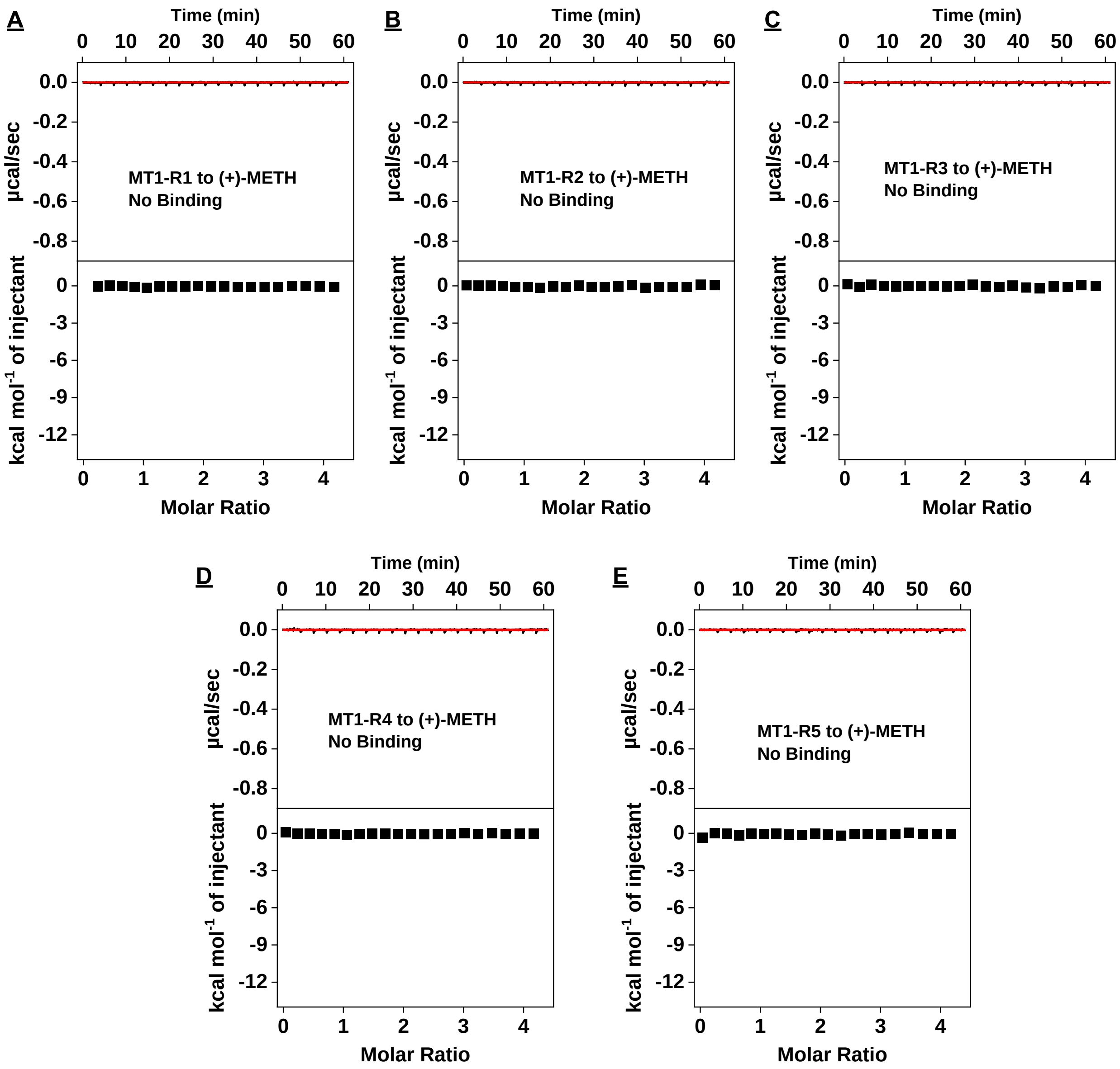

Figure S10. Binding affinity of aptamers discovered in the first trial of library-immobilized SELEX for (+)-methamphetamine based on ITC. Top panels display the heat generated from each titration of (+)-methamphetamine into (**A**) MT1-R1, (**B**) MT1-R2, (**C**) MT1-R3, (**D**) MT1-R4, and (**E**) MT1-R5. Bottom panels show the integrated heat of each titration after correcting for the heat of dilution of the titrant.

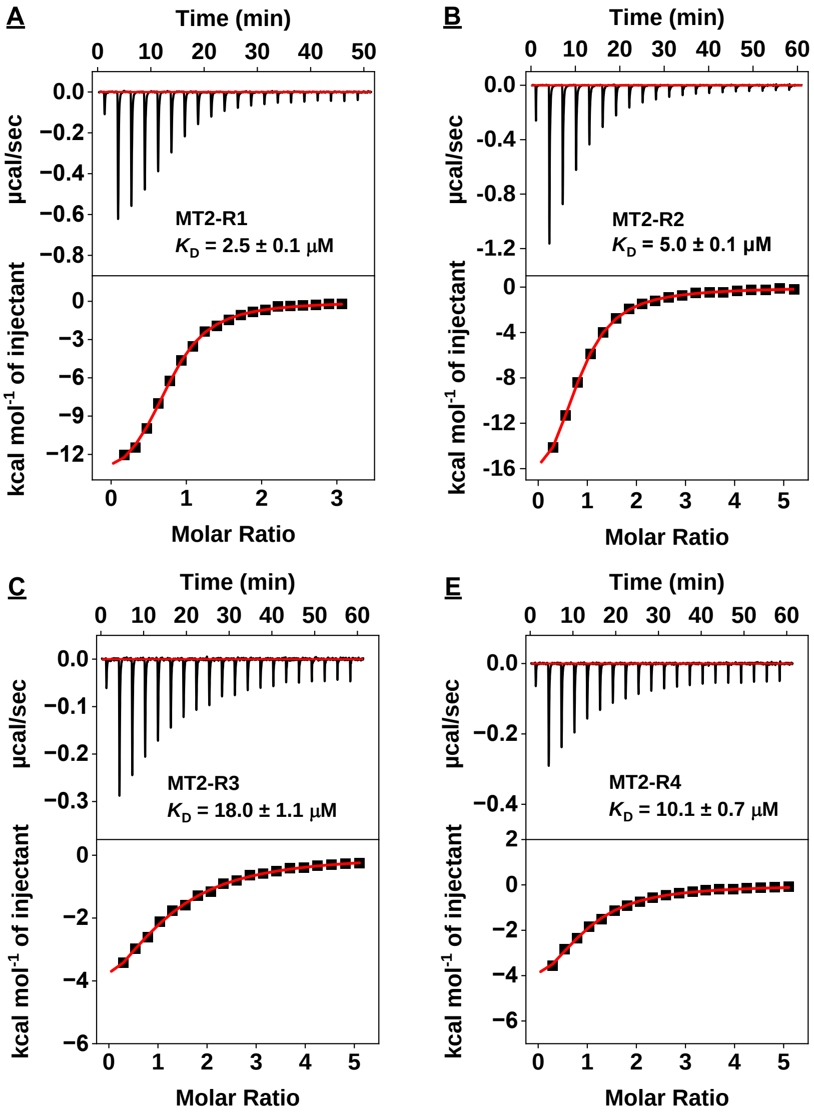

Figure S11. Binding affinity of aptamers discovered in the second trial of library-immobilized SELEX to (+)-methamphetamine based on ITC. Top panels display the heat generated from each titration of (+)-methamphetamine into (**A**) MT2-R1, (**B**) MT2-R2, (**C**) MT2-R3, and (**D**) MT2-R4. Bottom panels show the integrated heat of each titration after correcting for the heat of dilution of the titrant.

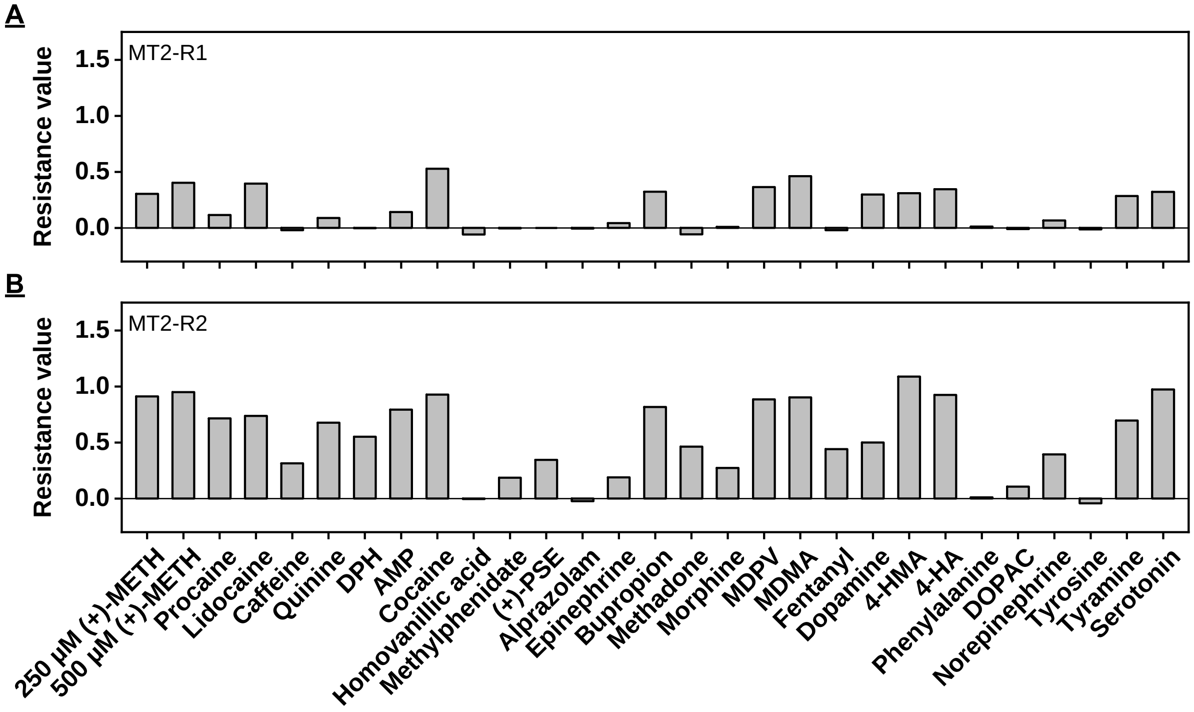

Figure S12. Screening the specificity of two aptamer candidates (MT2-R1 and MT2-R2) from the second SELEX trial using our exonuclease digestion assay. Plots show resistance value obtained from time-course digestion of (**A**) MT2-R1 and (**B**) MT2-R2 in the presence of 250 µM selection target and various interferents, except for alprazolam (50 µM).

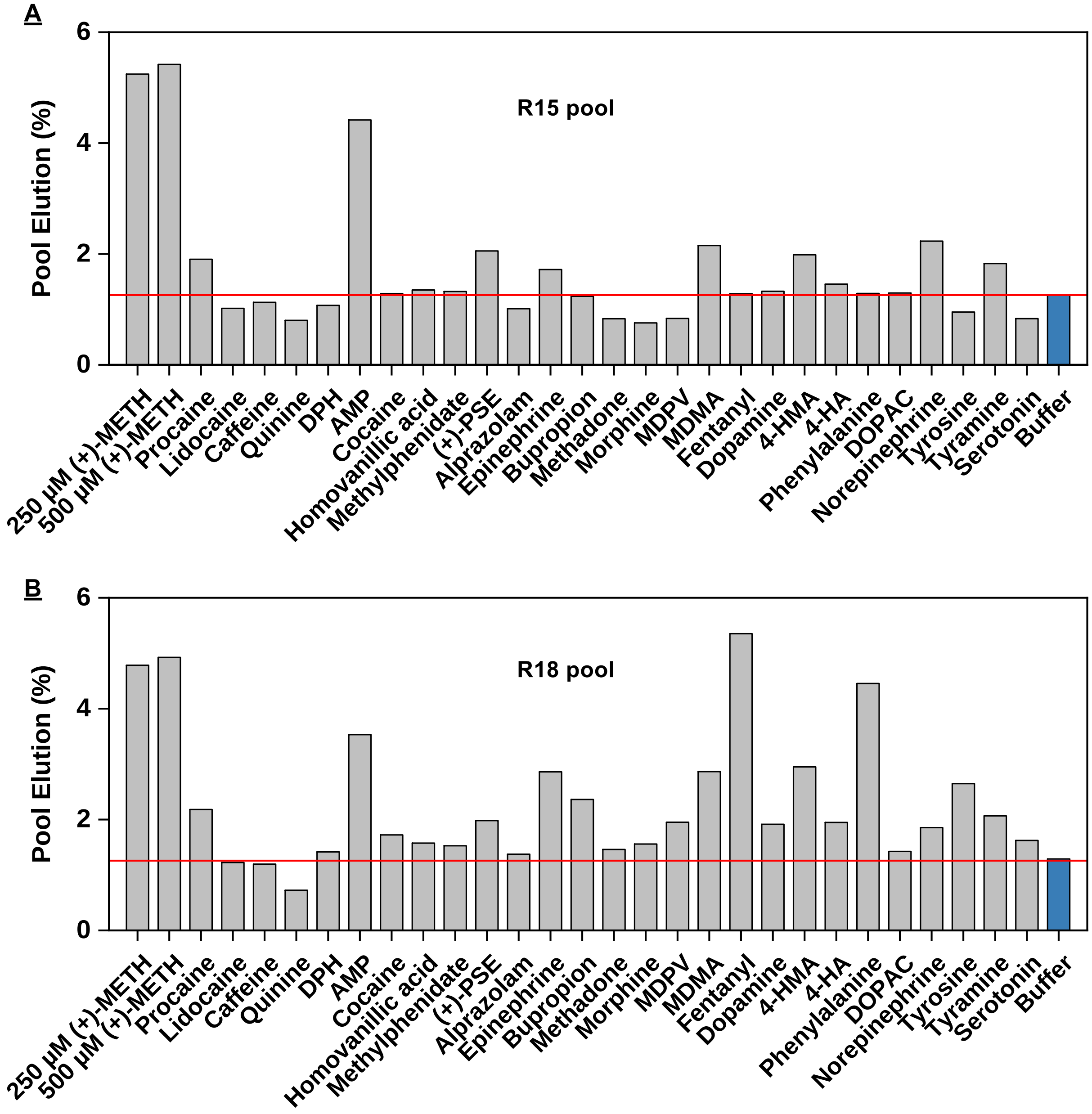

Figure S13. Specificity of the enriched pools from the third trial of library-immobilized SELEX for (+)-methamphetamine as determined using the gel-elution assay. Pool elution by methamphetamine, each counter-target, and buffer alone (blue bar and red line) is plotted for (**A**) Round 15 and (**B**) Round 18.

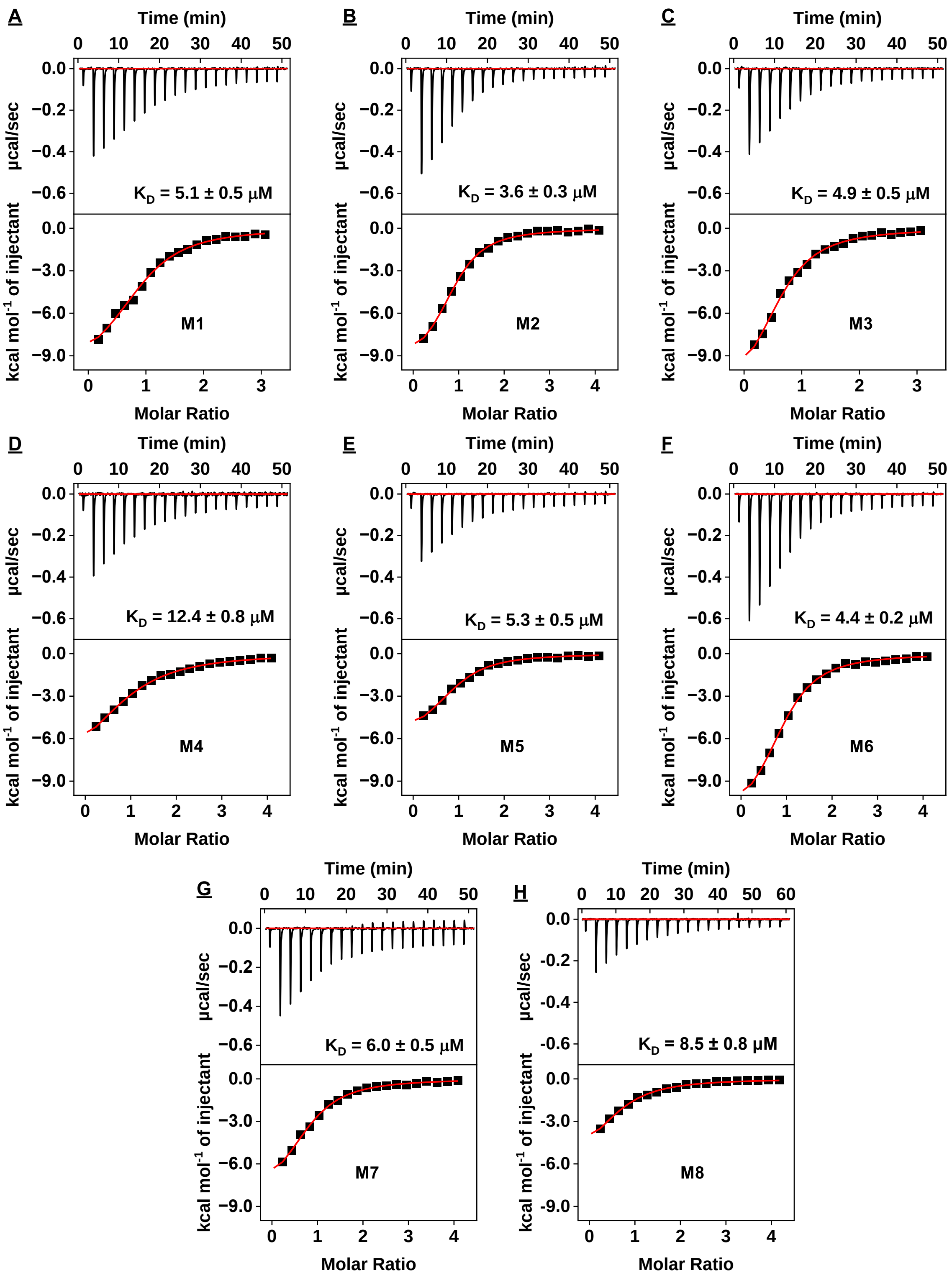

Figure S14. Binding affinity of aptamers discovered in the third trial of library-immobilized SELEX to (+)-methamphetamine based on ITC. Top panels display the heat generated from each titration of (+)-methamphetamine into (**A**) M1, (**B**) M2, (**C**) M3, (**D**) M4, (**E**) M5, (**F**) M6, (**G**) M7, and (**H**) M8. Bottom panels show the integrated heat of each titration after correcting for the heat of dilution of the titrant.

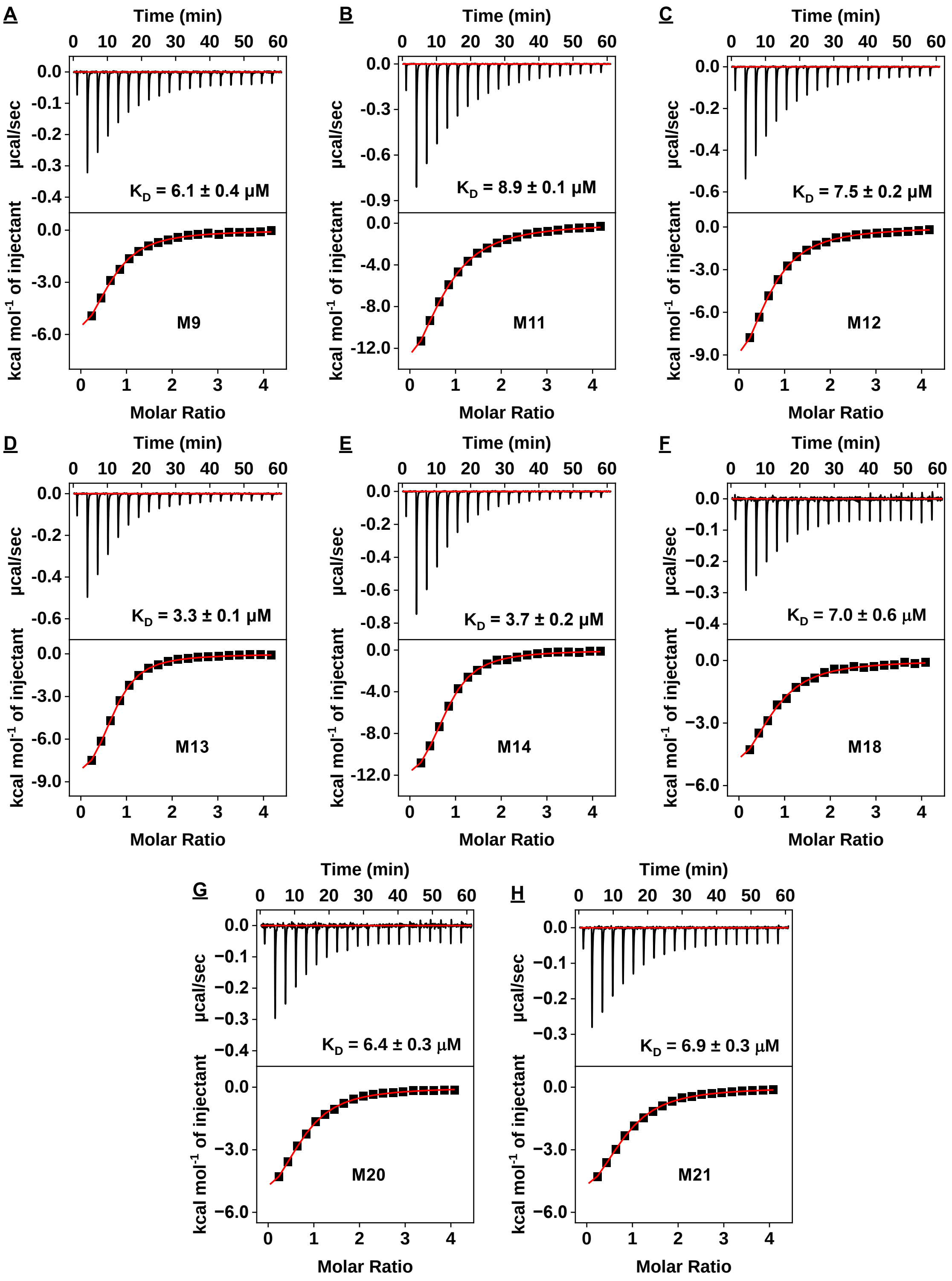

Figure S15. Binding affinity of aptamers discovered in the third trial of library-immobilized SELEX for (+)-methamphetamine based on ITC. Top panels display the heat generated from each titration of (+)-methamphetamine into (**A**) M9, (**B**) M11, (**C**) M12, (**D**) M13, (**E**) M14, (**F**) M18, (**G**) M20, and (**H**) M21. Bottom panels show the integrated heat of each titration after correcting for the heat of dilution of the titrant.

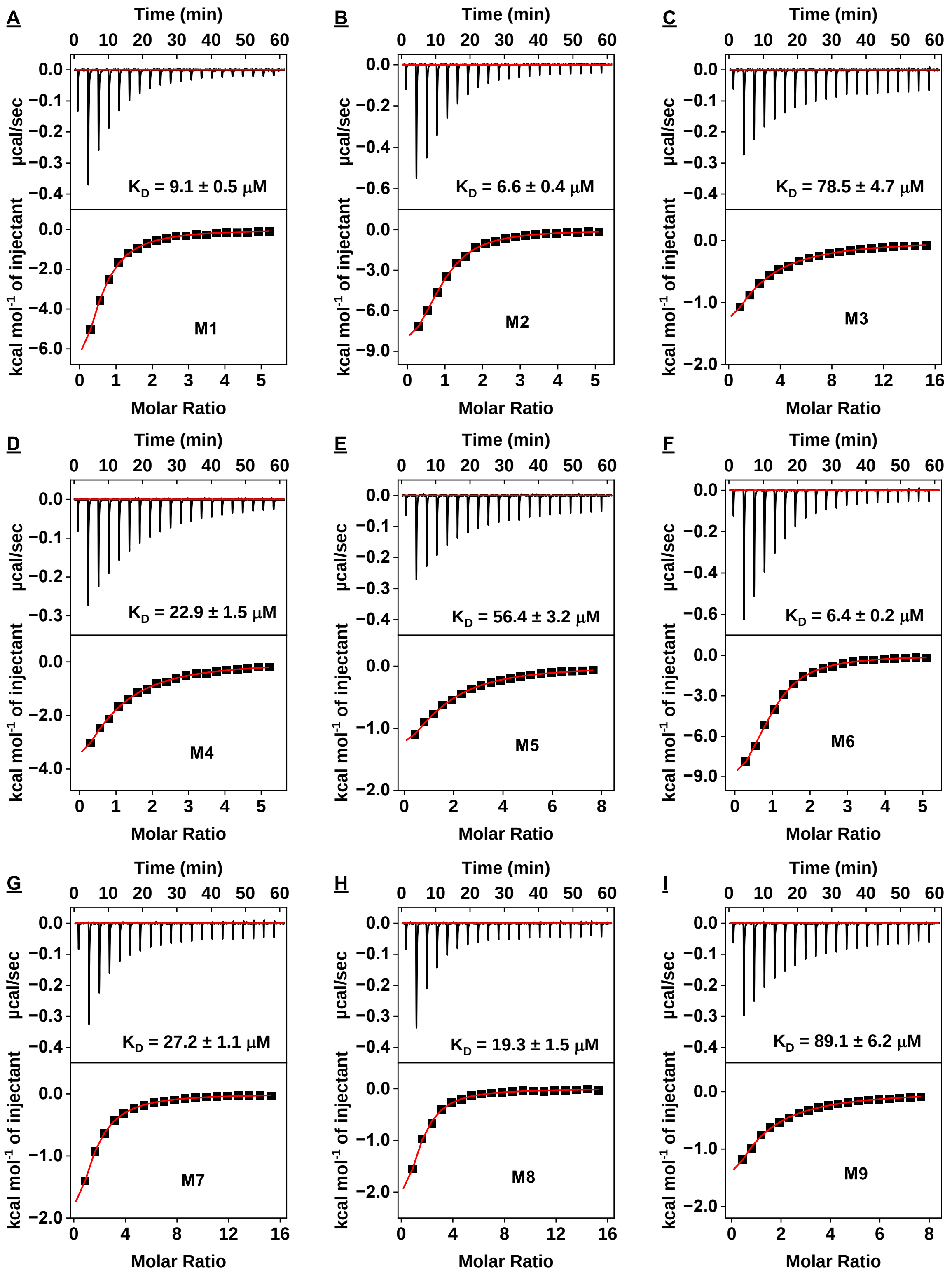

Figure S16. Binding affinity of aptamers discovered in the third trial of library-immobilized SELEX for amphetamine based on ITC. Top panels display the heat generated from each titration of amphetamine into (**A**) M1, (**B**) M2, (**C**) M3, (**D**) M4, (**E**) M5, (**F**) M6, (**G**) M7, (**H**) M8, and (**I**) M9. Bottom panels show the integrated heat of each titration after correcting for the heat of dilution of the titrant.

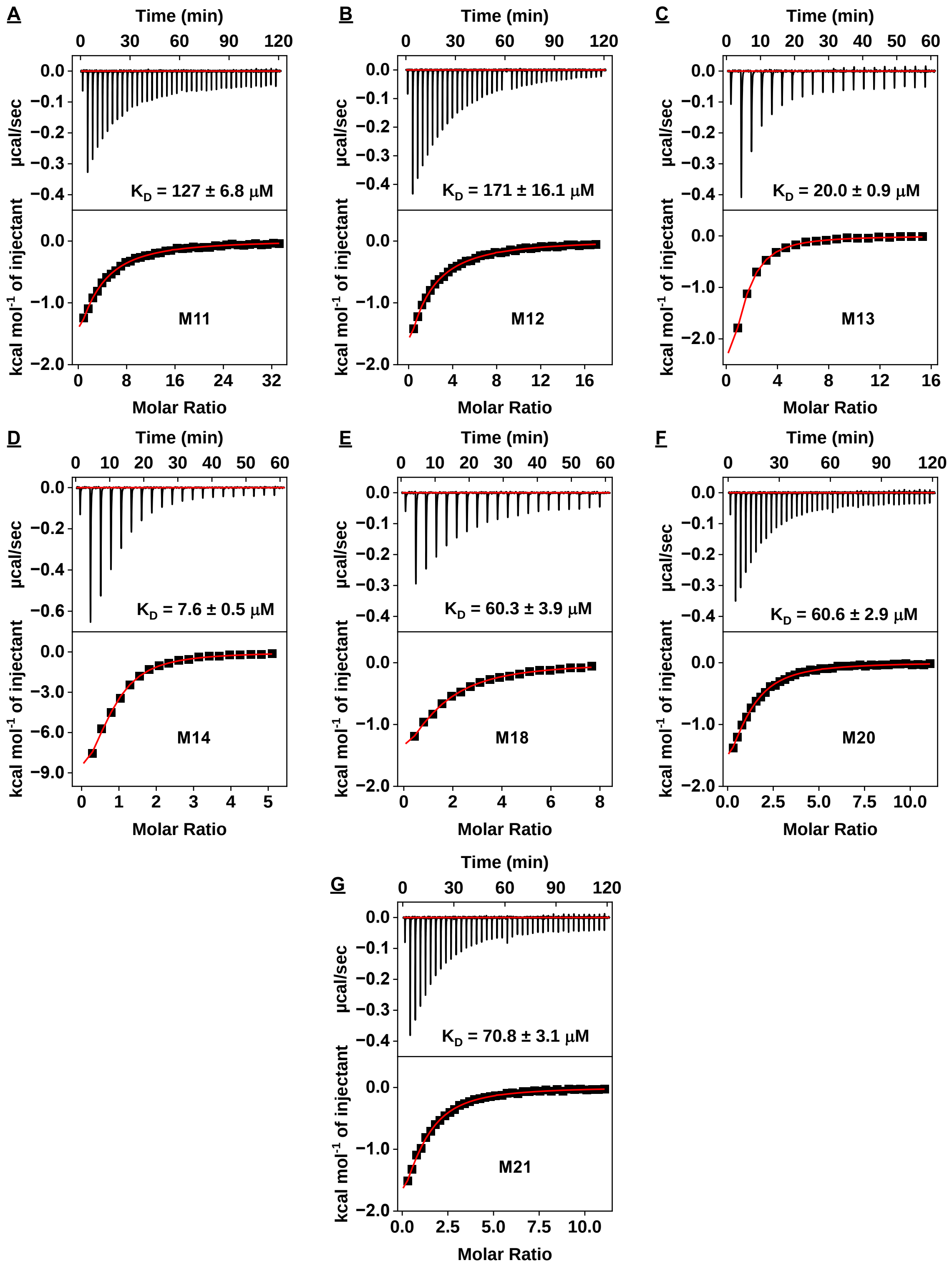

Figure S17. Binding affinity of aptamers discovered in the third trial of library-immobilized SELEX for amphetamine based on ITC. Top panels display the heat generated from each titration of amphetamine into (**A**) M11, (**B**) M12, (**C**) M13, (**D**) M14, (**E**) M18, (**F**) M20, and (**G**) M21. Bottom panels show the integrated heat of each titration after correcting for the heat of dilution of the titrant.

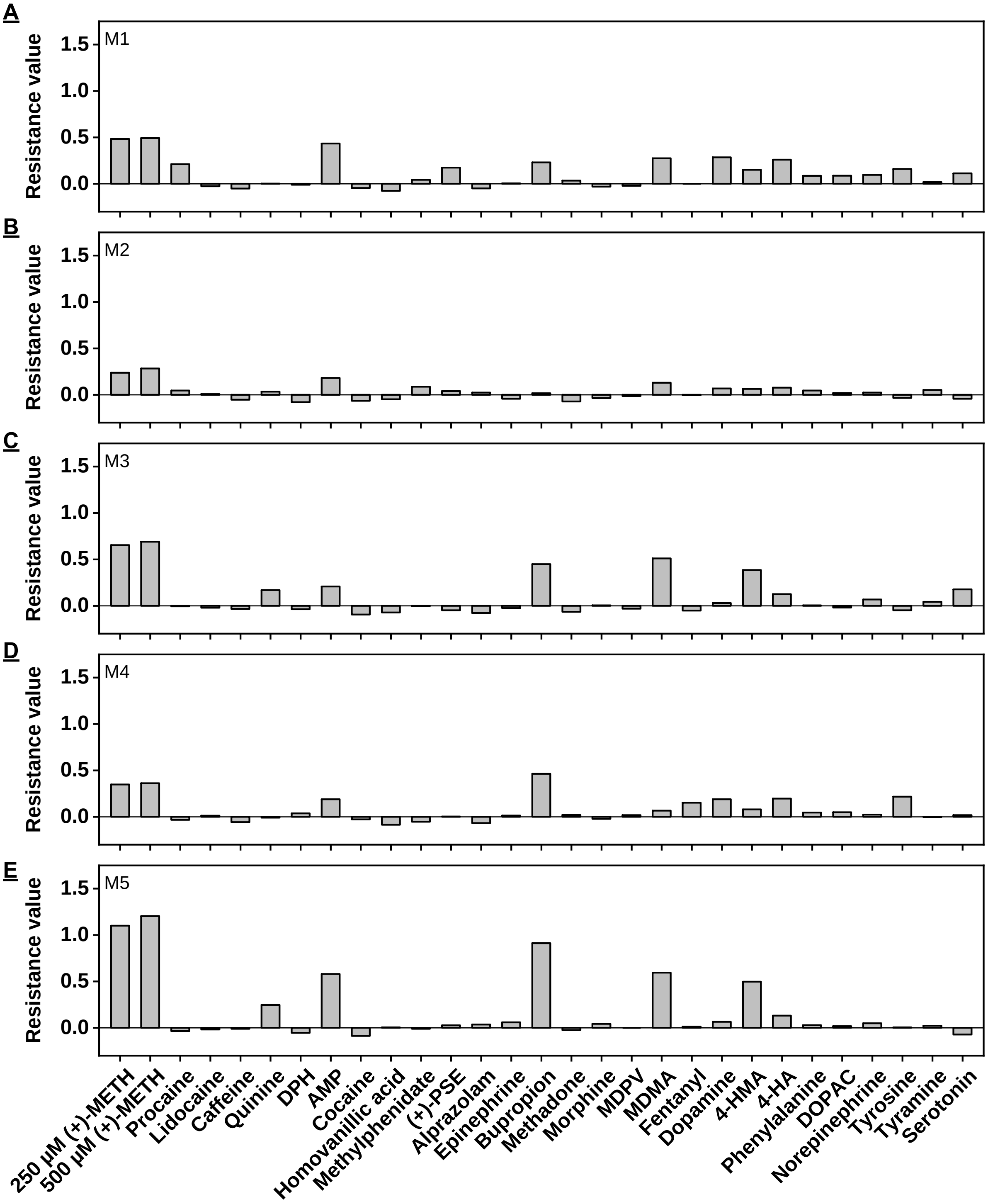

Figure S18. Screening the specificity of five aptamer candidates from the third SELEX trial using our exonuclease digestion assay. Plots show resistance value obtained from time-course digestion of (**A**) M1, (**B**) M2, (**C**) M3, (**D**) M4, and (**E**) M5 in the presence of 250 µM selection target and various interferents, except for alprazolam (50 µM).

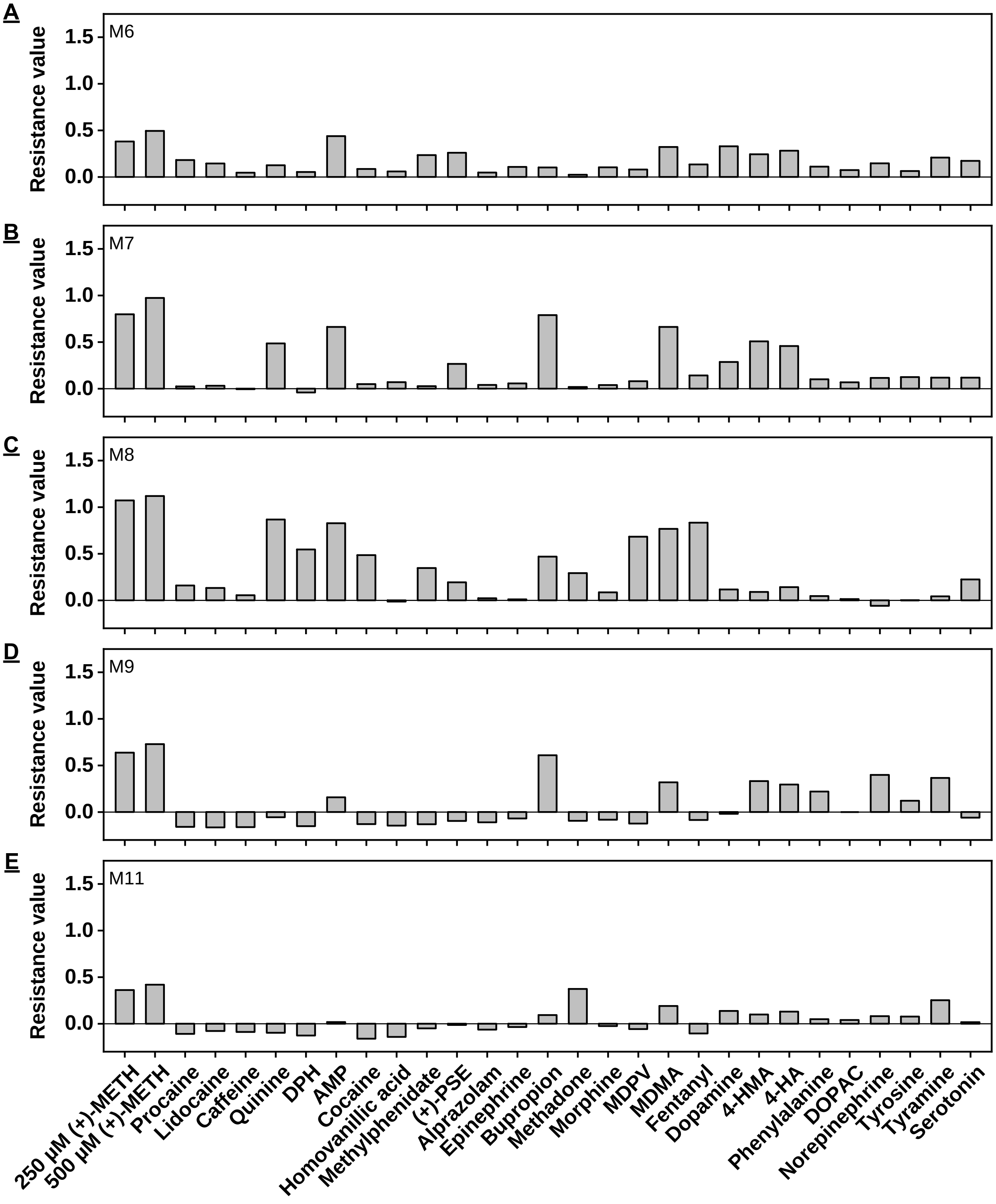

Figure S19. Screening the specificity of five aptamer candidates from the third SELEX trial using our exonuclease digestion assay. Plots show resistance value obtained from time-course digestion of (**A**) M6, (**B**) M7, (**C**) M8, (**D**) M9, and (**E**) M11 in the presence of 250 μM selection target and various interferents, except for alprazolam (50 μM).

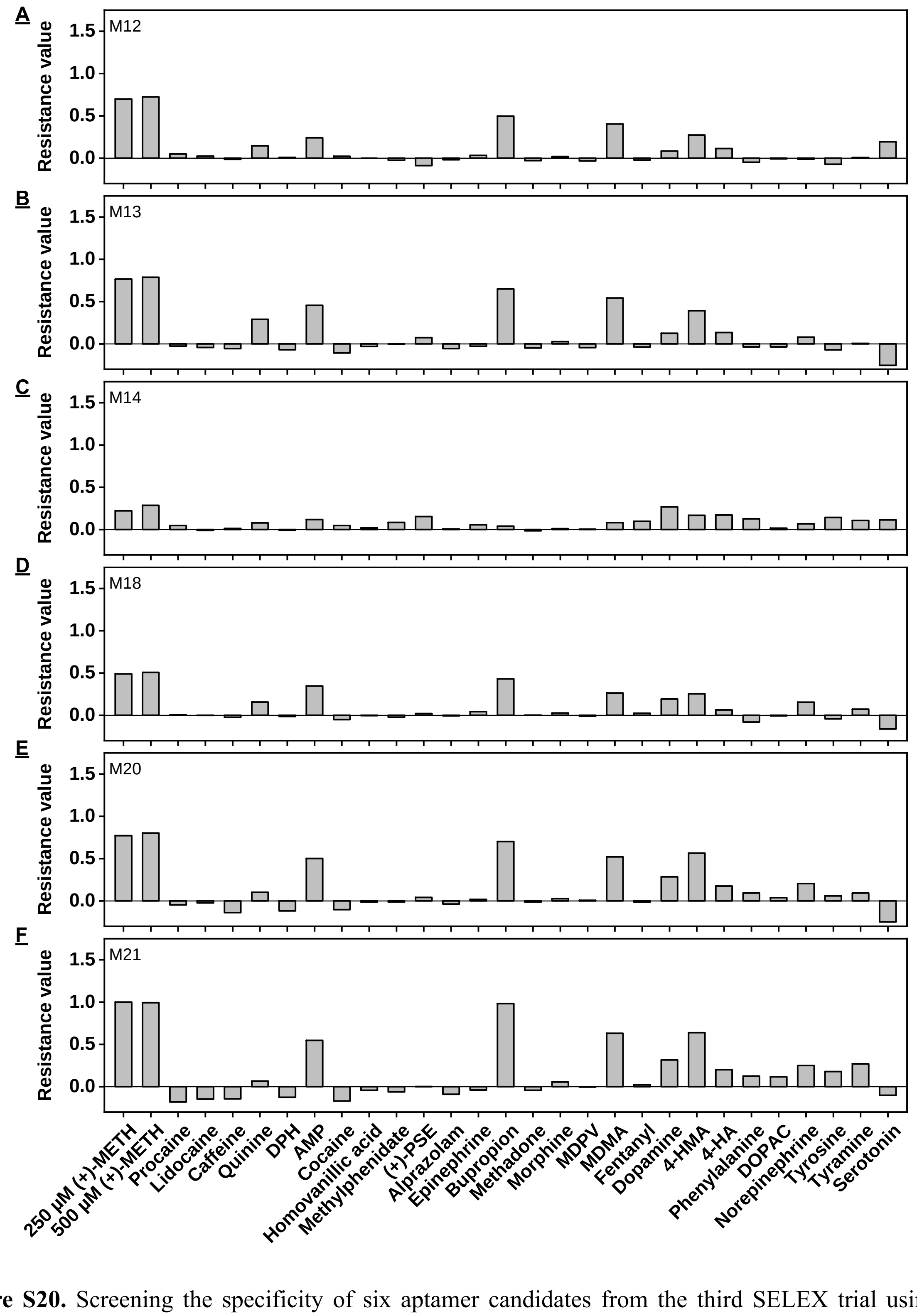

Figure S20. Screening the specificity of six aptamer candidates from the third SELEX trial using our exonuclease digestion assay. Plots show resistance value obtained from time-course digestion of (**A**) M12, (**B**) M13, (**C**) M14, (**D**) M18, (**E**) M20 and (**F**) M21 in the presence of 250 µM selection target and various interferents, except for alprazolam (50 µM).

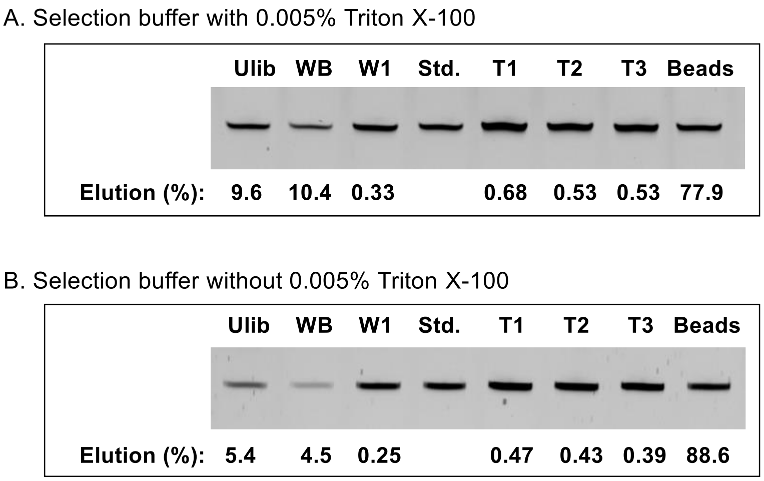

Figure S21. The effect of including Triton X-100 in the selection buffer on washing efficiency during library-immobilized SELEX. The naive library was hybridized with biotinylated cDNA, immobilized onto streptavidin-coated agarose beads, and then washed with selection buffer (**A**) with or (**B**) without 0.005% Triton-X100, and then finally challenged with methamphetamine. Samples eluted from the column were collected and analyzed by PAGE. ‘Ulib’ = non-immobilized library after incubating library-cDNA complexes with streptavidin-coated agarose. ‘WB’ = library washed away by ten washes with 250 µL selection buffer. ‘W1’ = library eluted by one wash of 250 µL selection buffer prior to challenging the library with target. ‘Std’ = a 10 nM standard made using the naïve library. ‘T1’, ‘T2’, and ‘T3’ = elution of library after challenging with three sequential 250 µL aliquots of methamphetamine in selection buffer (without Triton X-100). ‘Beads’ = library remaining on the beads at the end of the selection round.

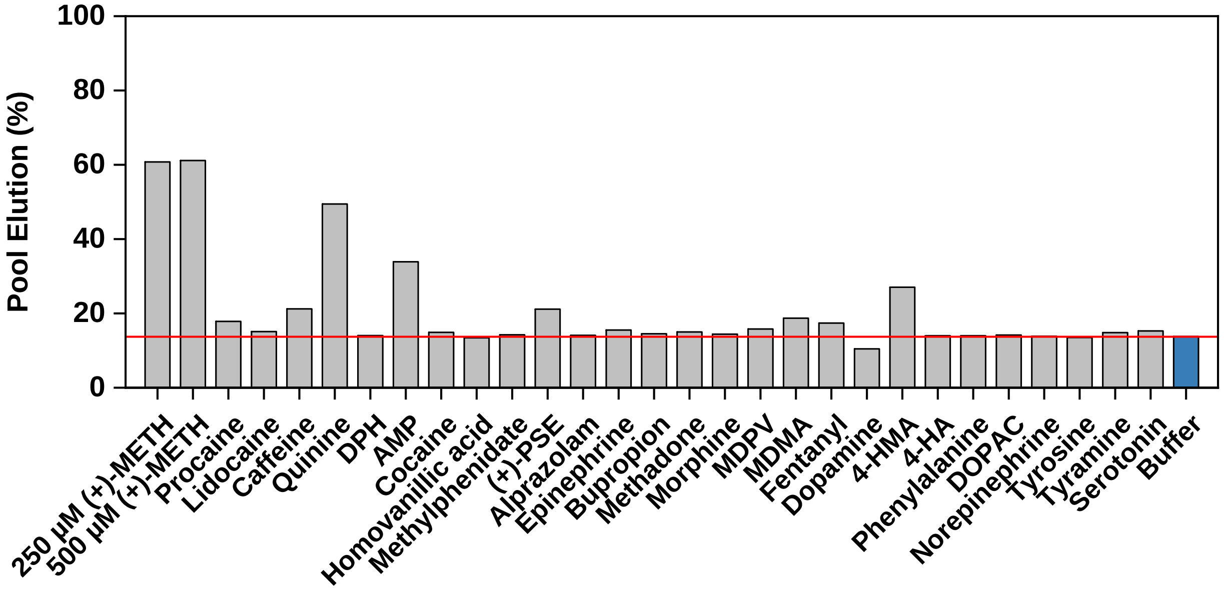

Figure S22. Specificity of the Round 13 pool from the fourth trial of library-immobilized SELEX for (+)-methamphetamine as determined using the gel-elution assay. Red line indicates the threshold of elution by the buffer-only negative control.

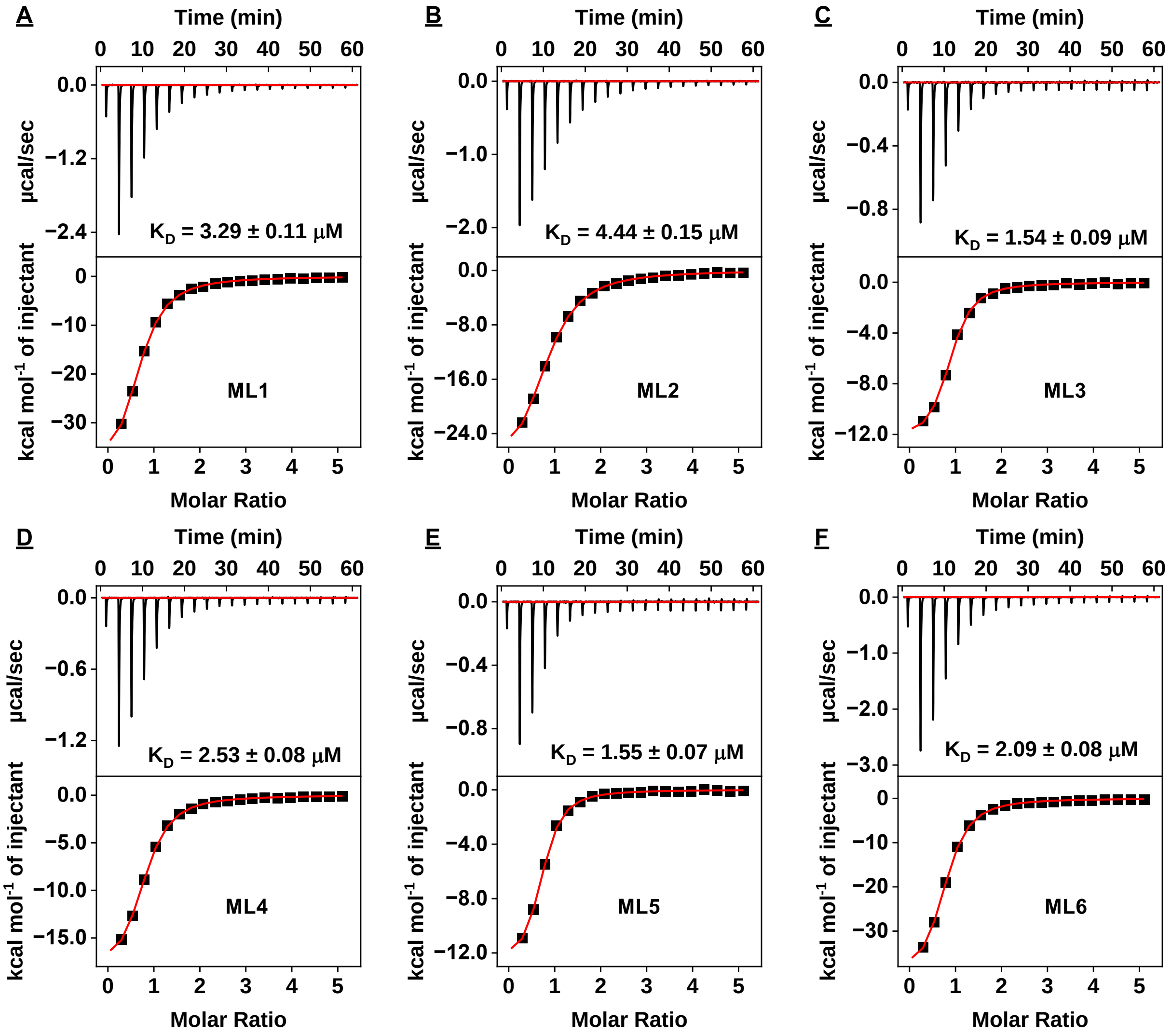

Figure S23. Binding affinity of aptamers isolated in the fourth trial of library-immobilized SELEX for (+)-methamphetamine based on ITC. Top panels display the heat generated from each titration of (+)-methamphetamine into (**A**) ML1, (**B**) ML2, (**C**) ML3, (**D**) ML4, (**E**) ML5, and (**F**) ML6. Bottom panels show the integrated heat of each titration after correcting for the heat of dilution of the titrant.

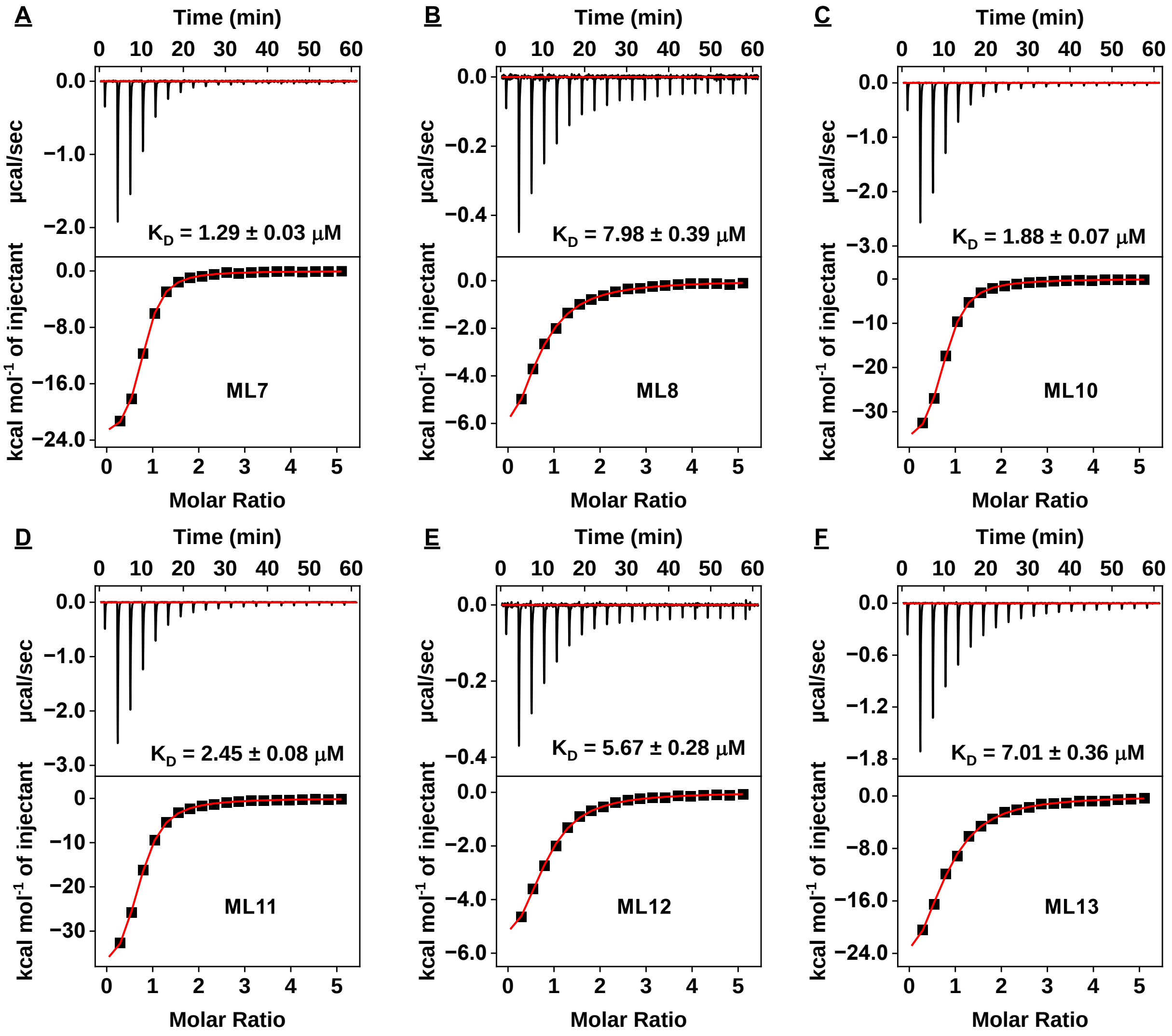

Figure S24. Binding affinity of aptamers isolated in the fourth trial of library-immobilized SELEX for (+)-methamphetamine based on ITC. Top panels display the heat generated from each titration of (+)-methamphetamine into (**A**) ML7, (**B**) ML8, (**C**) ML10, (**D**) ML11, (**E**) ML12, and (**F**) ML13. Bottom panels show the integrated heat of each titration after correcting for the heat of dilution of the titrant.

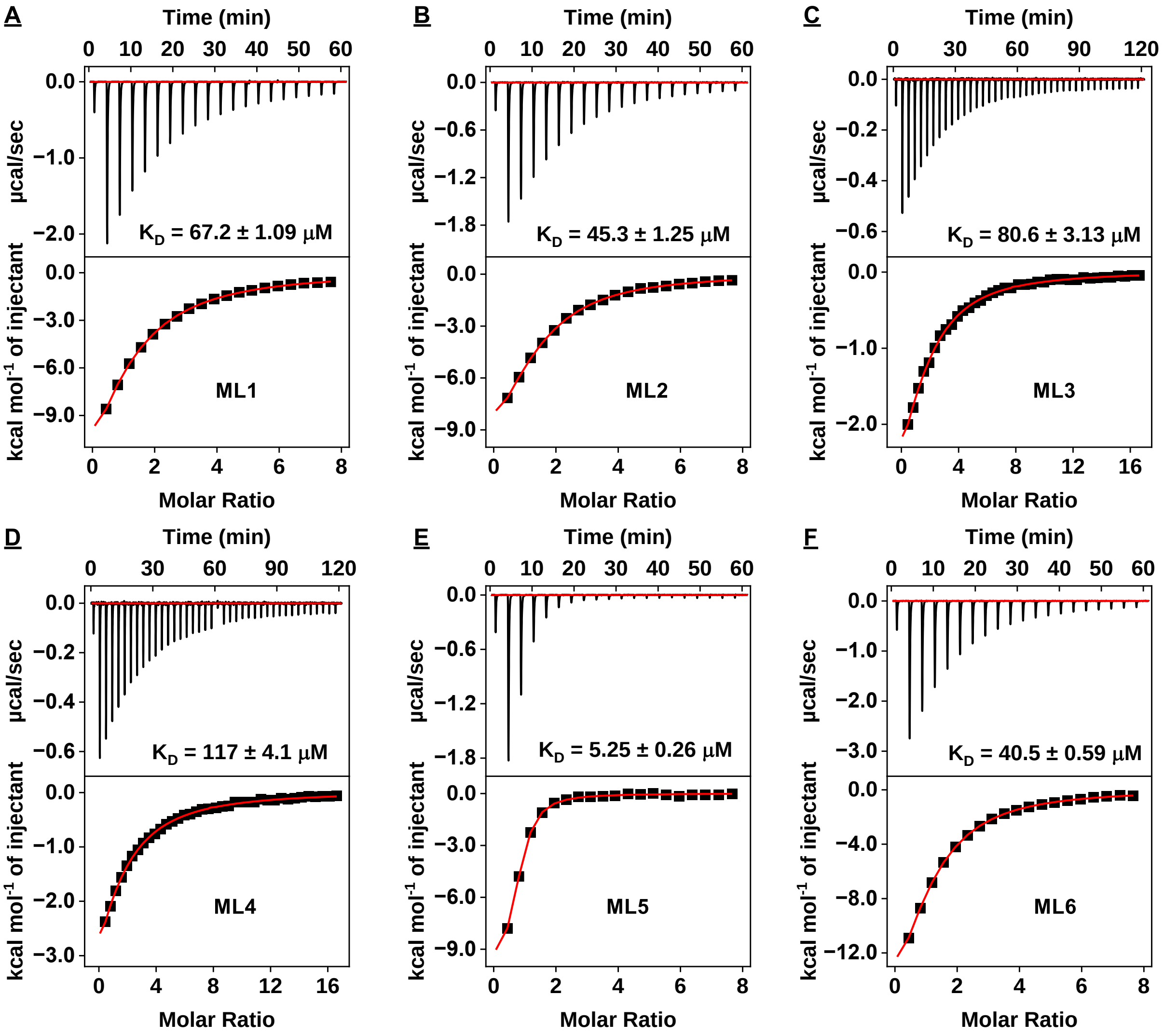

Figure S25. Binding affinity of aptamers discovered in the fourth trial of library-immobilized SELEX for amphetamine based on ITC. Top panels display the heat generated from each titration of amphetamine into (**A**) ML1, (**B**) ML2, (**C**) ML3, (**D**) ML4, (**E**) ML5, and (**F**) ML6. Bottom panels show the integrated heat of each titration after correcting for the heat of dilution of the titrant.

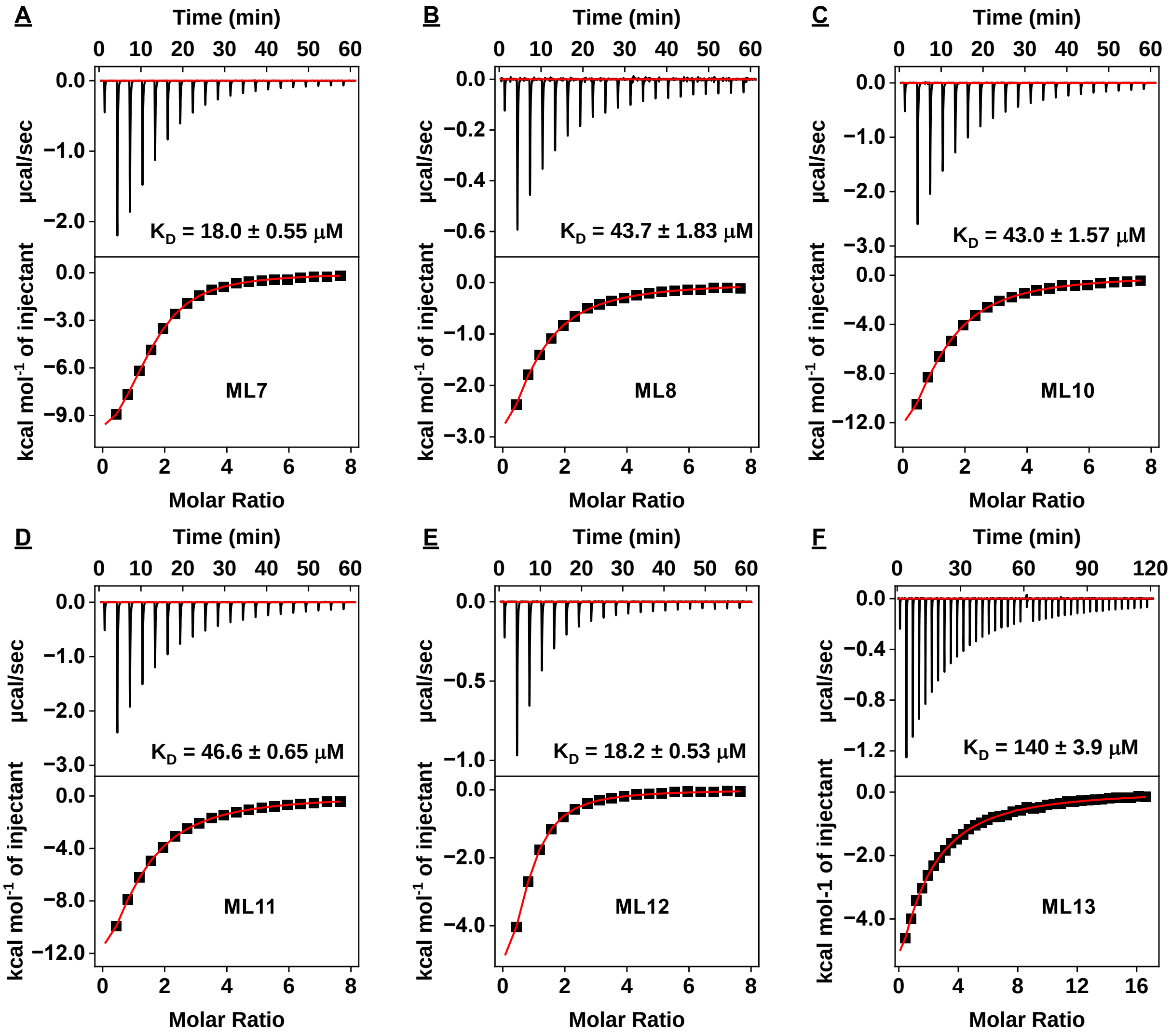

Figure S26. Binding affinity of aptamers discovered in the fourth trial of library-immobilized SELEX for amphetamine based on ITC. Top panels display the heat generated from each titration of amphetamine into (**A**) ML7, (**B**) ML8, (**C**) ML10, (**D**) ML11, (**E**) ML12, and (**F**) ML13. Bottom panels show the integrated heat of each titration after correcting for the heat of dilution of the titrant.

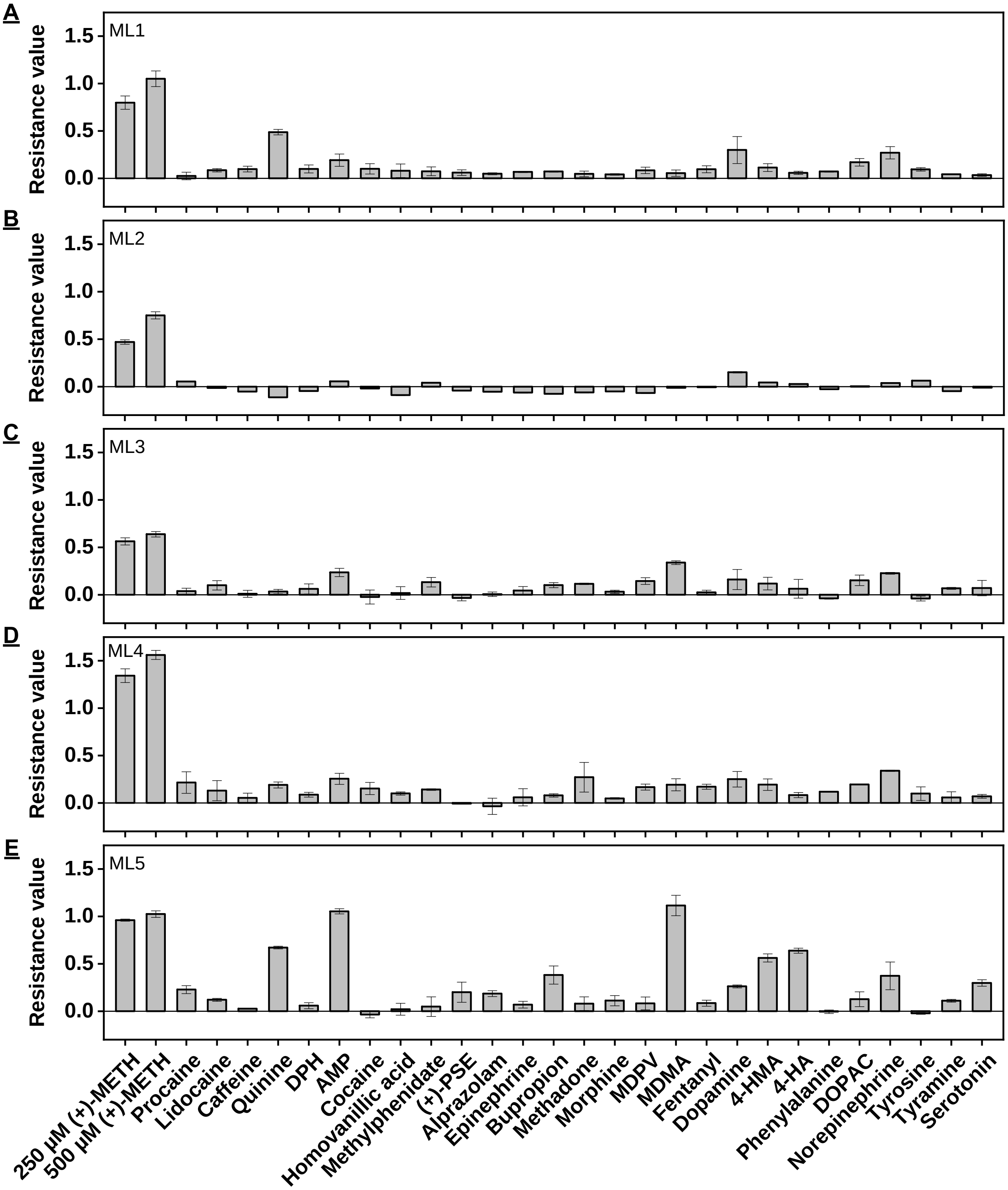

Figure S27. Screening the specificity of five aptamer candidates from the fourth SELEX trial using our exonuclease digestion assay. Plots show resistance value obtained from time-course digestion of (**A**) ML1, (**B**) ML2, (**C**) ML3, (**D**) ML4, and (**E**) ML5 in the presence of 250 μM selection target and various interferents, except for alprazolam (50 μM).

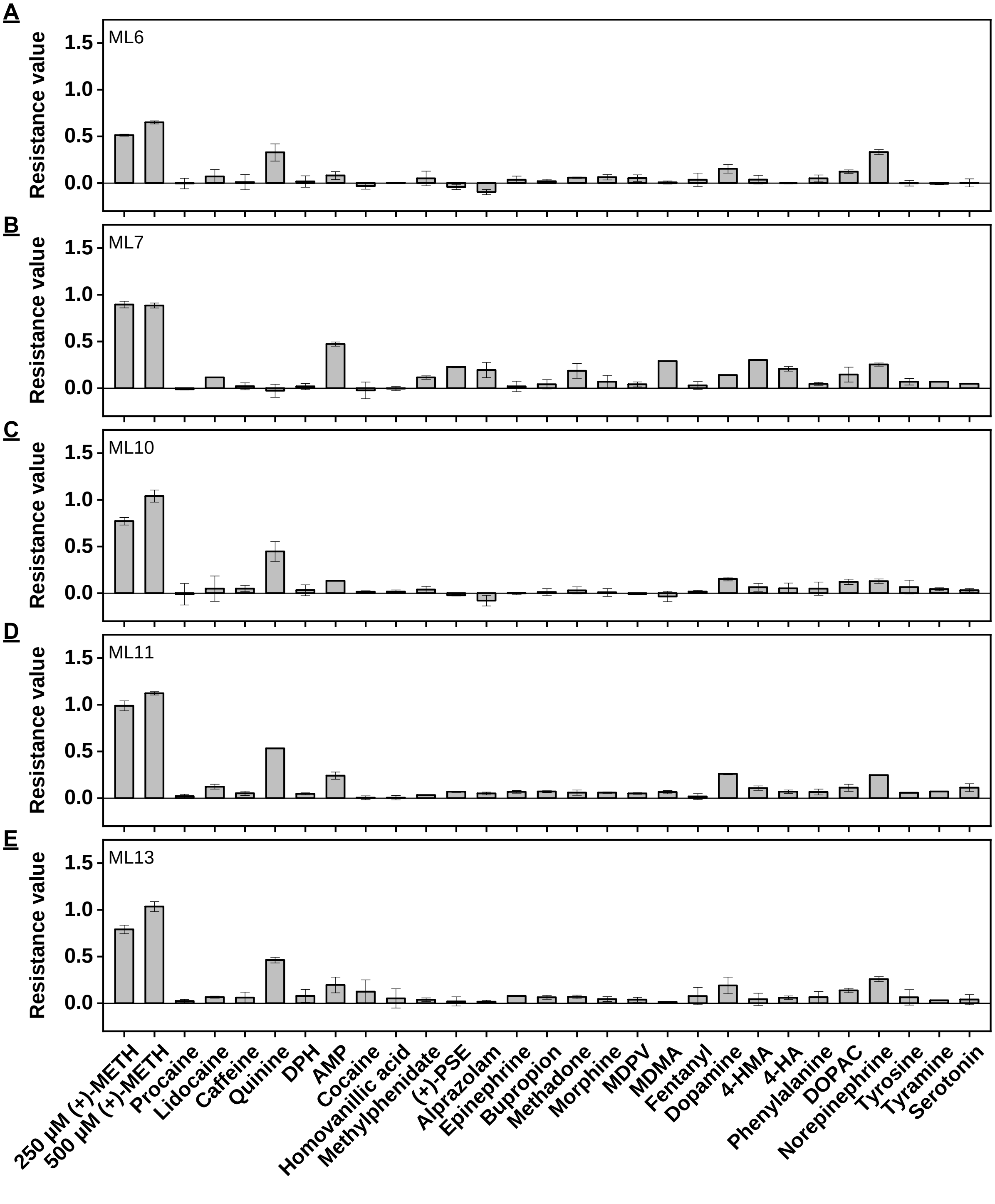

Figure S28. Screening the specificity of five aptamer candidates from the fourth SELEX trial using our exonuclease digestion assay. Plots show resistance value obtained from time-course digestion of (**A**) ML6, (**B**) ML7, (**C**) ML10, (**D**) ML11, and (**E**) ML13 in the presence of 250 µM selection target and various interferents, except for alprazolam (50 µM).

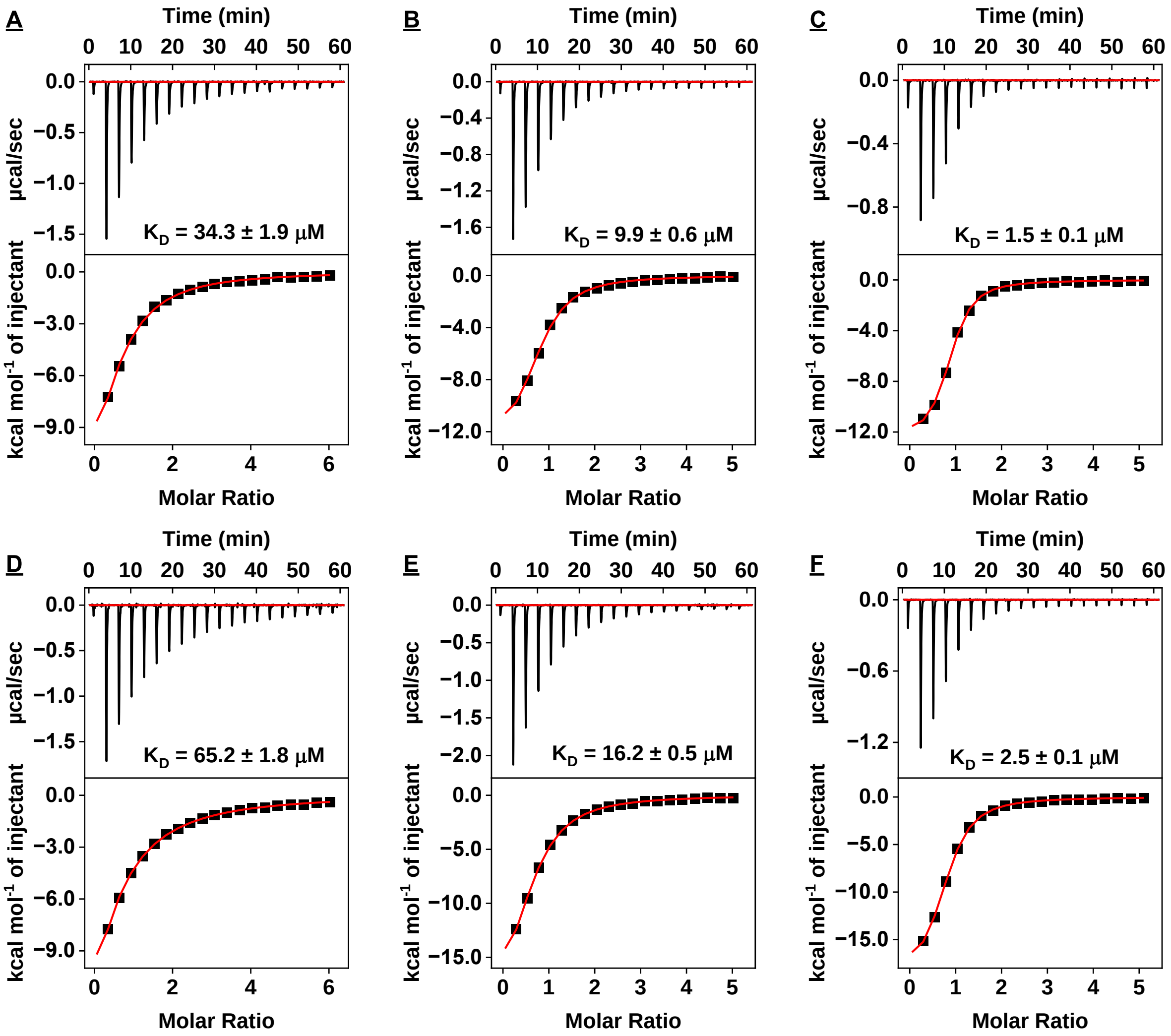

Figure S29. Binding affinity of aptamers for (+)-methamphetamine based on ITC. Top panels display the heat generated from each titration of (+)-methamphetamine into (**A–C**) ML3 and **(D–E**) ML4 in the presence of 1, 2 and 5 mM Mg^{2+}, respectively. Bottom panels show the integrated heat of each titration after correcting for the heat of titrant dilution.

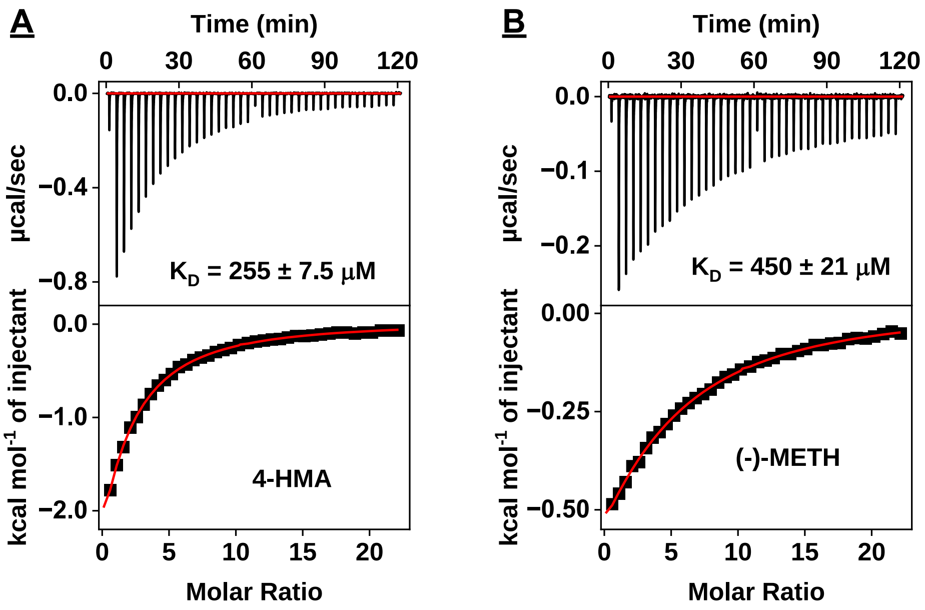

Figure S30. Binding affinity of ML4, an aptamer discovered in the fourth trial of library-immobilized SELEX, for interferents based on ITC. Top panels display the heat generated from each titration of ML4 into (**A**) 4-HMA and (**B**) (-)-METH. Bottom panels show the integrated heat of each titration after correcting for the heat of dilution of the titrant.

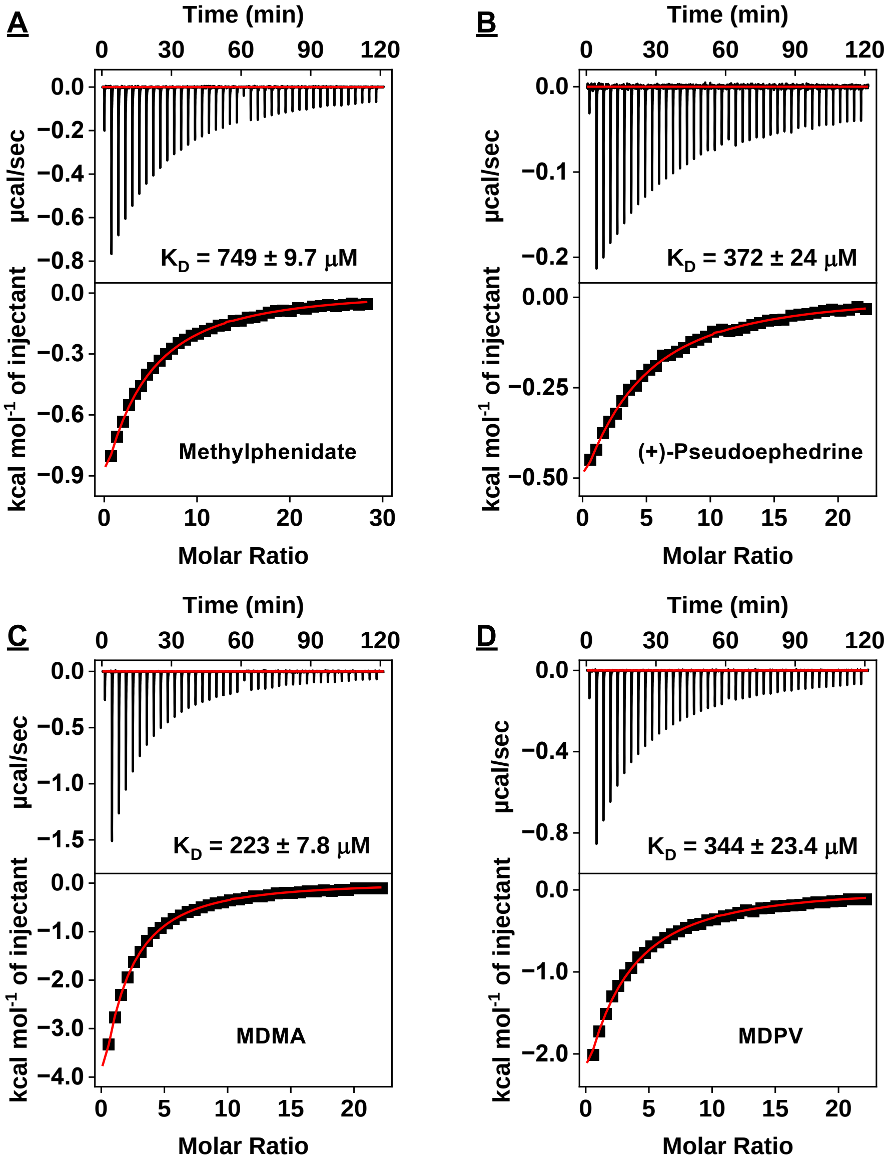

Figure S31. Binding affinity of ML4, an aptamer discovered in the fourth trial of library-immobilized SELEX, for interferents based on ITC. Top panels display the heat generated from each titration of ML4 into (**A**) methylphenidate, (**B**) (+)-pseudoephedrine, (C) MDMA, and (D) MDPV. Bottom panels show the integrated heat of each titration after correcting for the heat of dilution of the titrant.

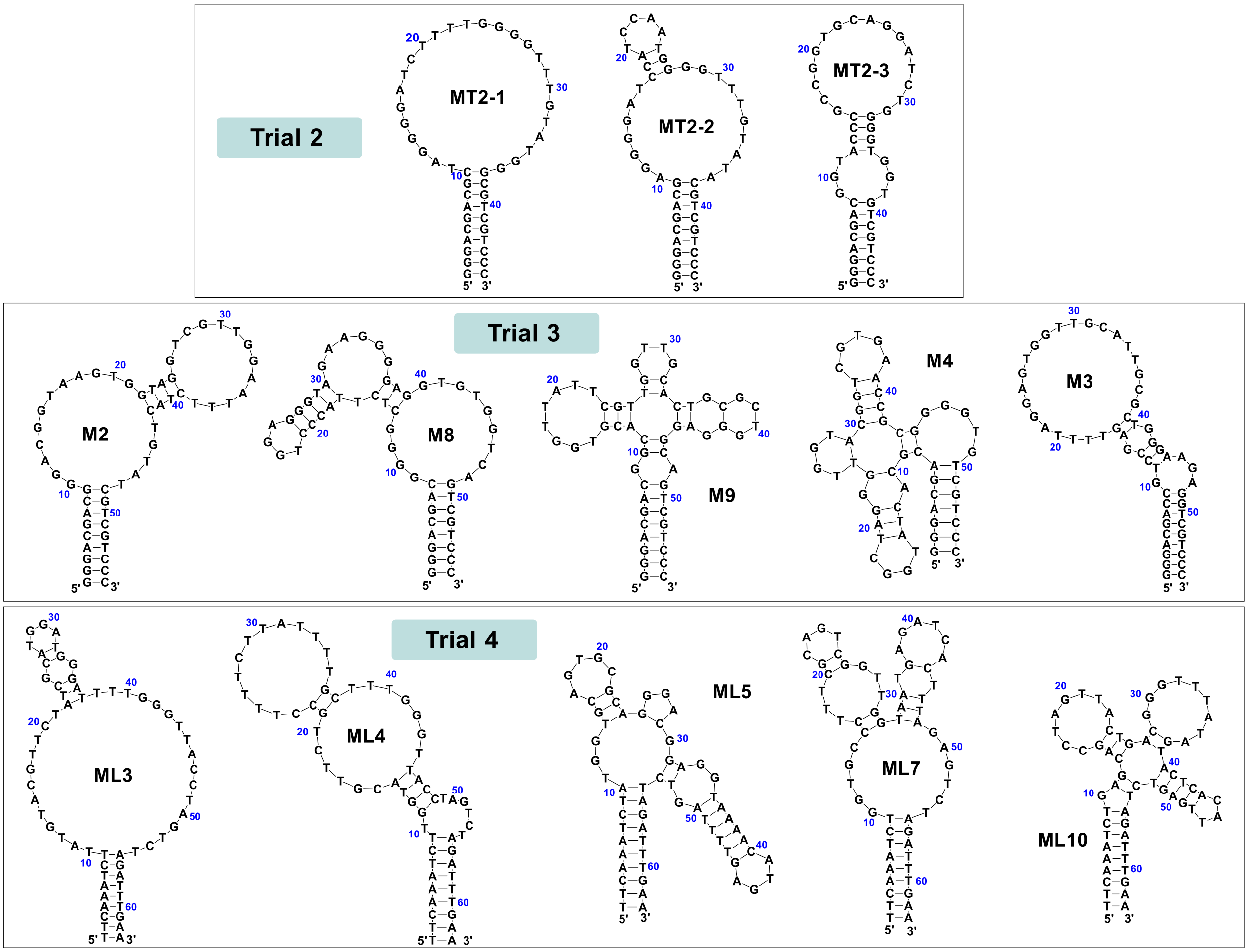

Figure S32. Secondary structures of methamphetamine aptamers obtained from different SELEX trials predicted by NUPACK.

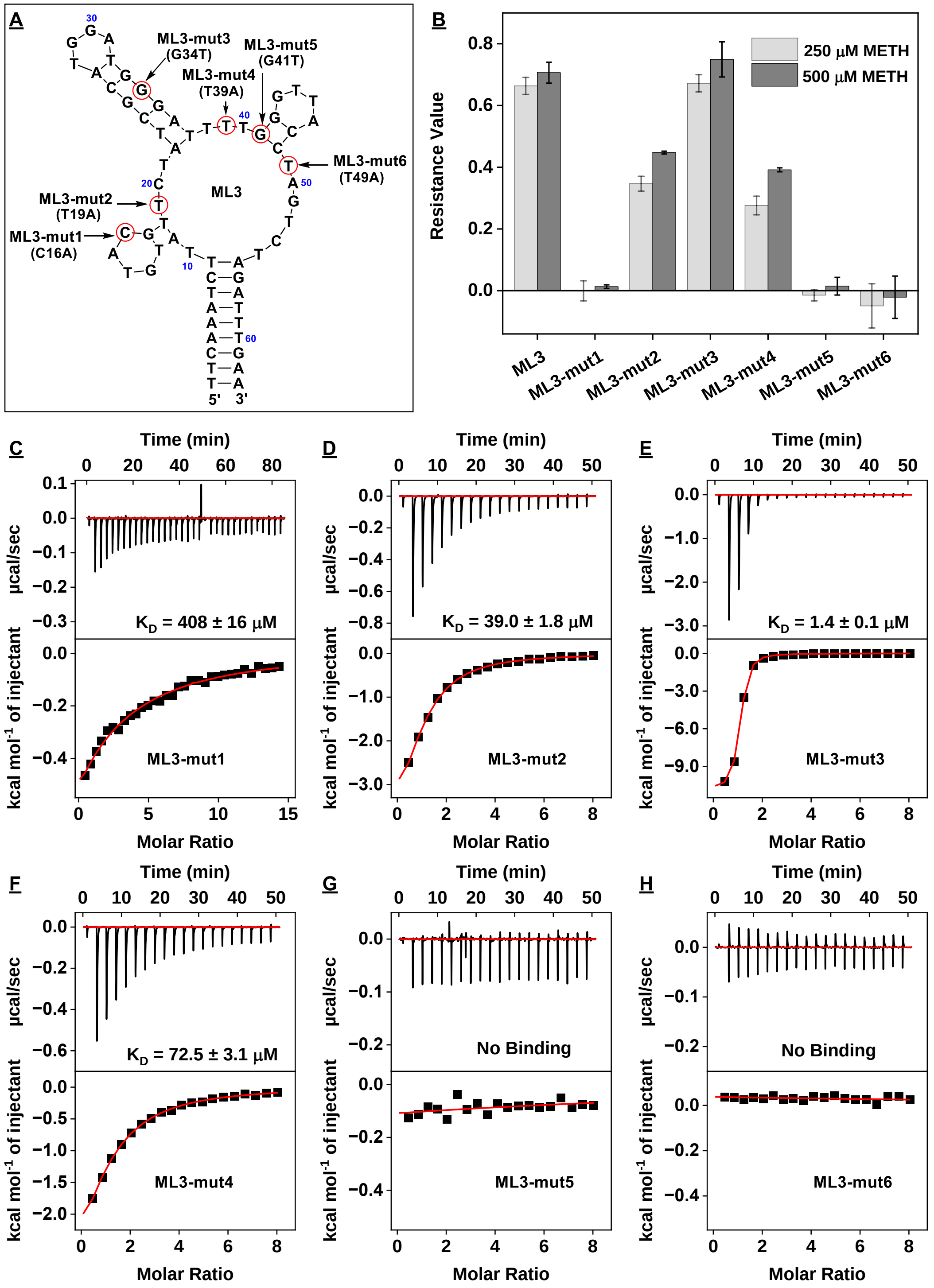

Figure S33. Mutational analysis of ML3. (**A**) Design of six point-mutants derived from ML3. (**B**) Resistance values of ML3 and six mutants determined by T5 exonuclease/exonuclease I digestion assay. (**C–H**) Binding affinity of mutated aptamers for (+)-methamphetamine based on ITC. Top panels display the heat generated from each titration of (+)-methamphetamine into (**C**) ML3-mut1, (**D**) ML3-mut2, (**E**) ML3-mut3, (**F**) ML3-mut4, (**G**) ML3-mut5, and (**H**) ML3-mut6. Bottom panels show the integrated heat of each titration after correcting for the heat of titrant dilution.

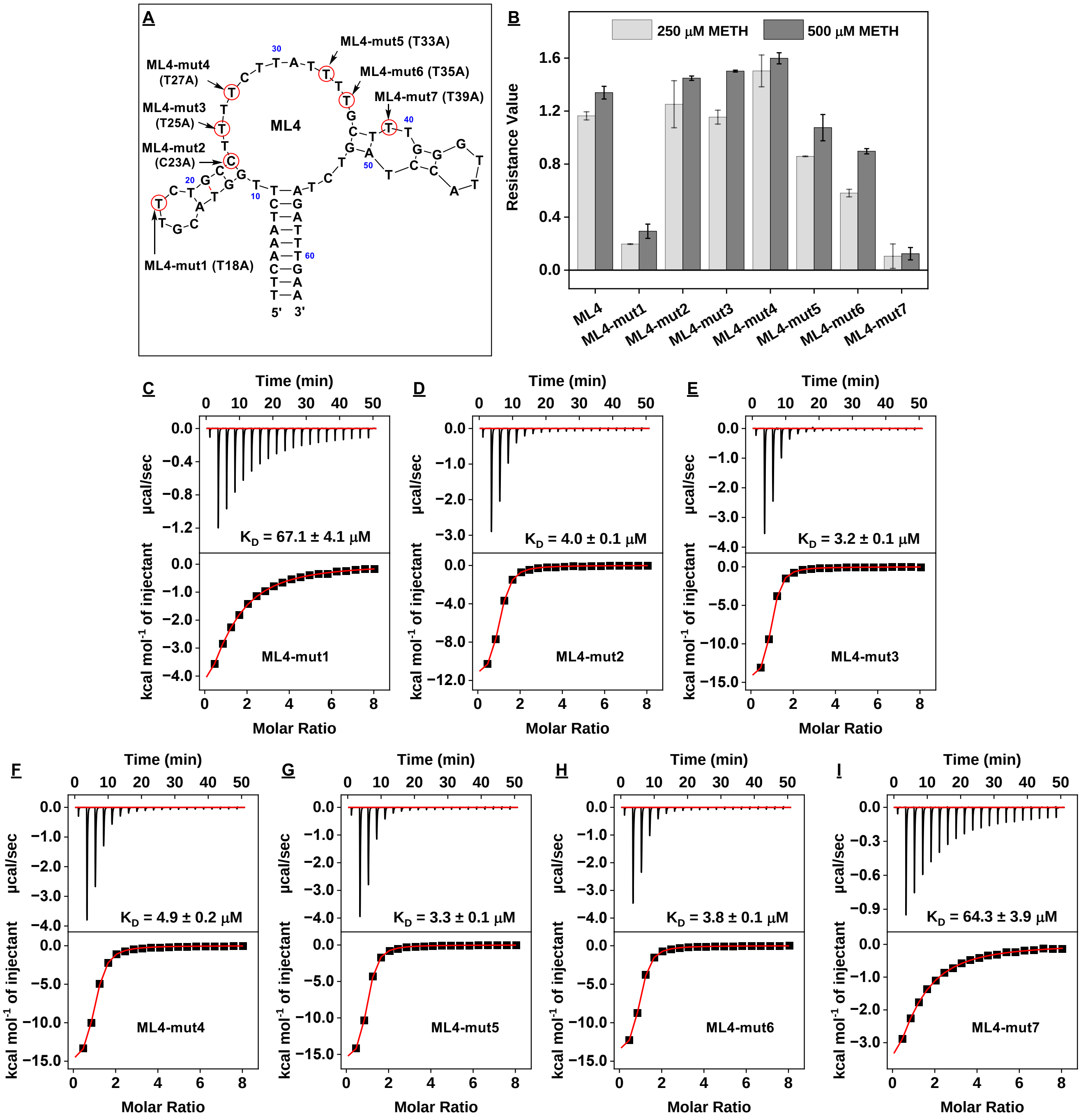

Figure S34. Mutational analysis of ML4. (**A**) Design of seven point-mutants derived from ML4. (**B**) Resistance values of ML4 and seven mutants determined by T5 exonuclease/exonuclease I digestion assay. (**C–I**) Binding affinity of mutated aptamers for (+)-methamphetamine based on ITC. Top panels display the heat generated from each titration of (+)-methamphetamine into (**C**) ML4-mut1, (**D**) ML4-mut2, (**E**) ML4-mut3, (**F**) ML4-mut4, (**G**) ML4-mut5, (**H**) ML4-mut6, and (**I**) ML4-mut7. Bottom panels show the integrated heat of each titration after correcting for the heat of titrant dilution.

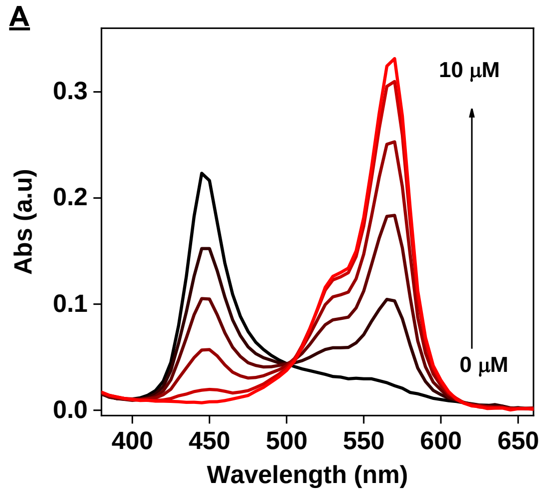

Figure S35. Binding of X-732-91B to aptamer ML4. (**A**) Absorbance spectra of solutions containing 4 μM X-732-91B with 0, 2, 4, 6, 8 or 10 μM ML4, where the black-to-red color gradient shows increasing aptamer concentrations. (**B**) Absorbance of X-732-91B monomers at 568 nm and H-aggregates at 450 nm plotted against the concentration of ML4.

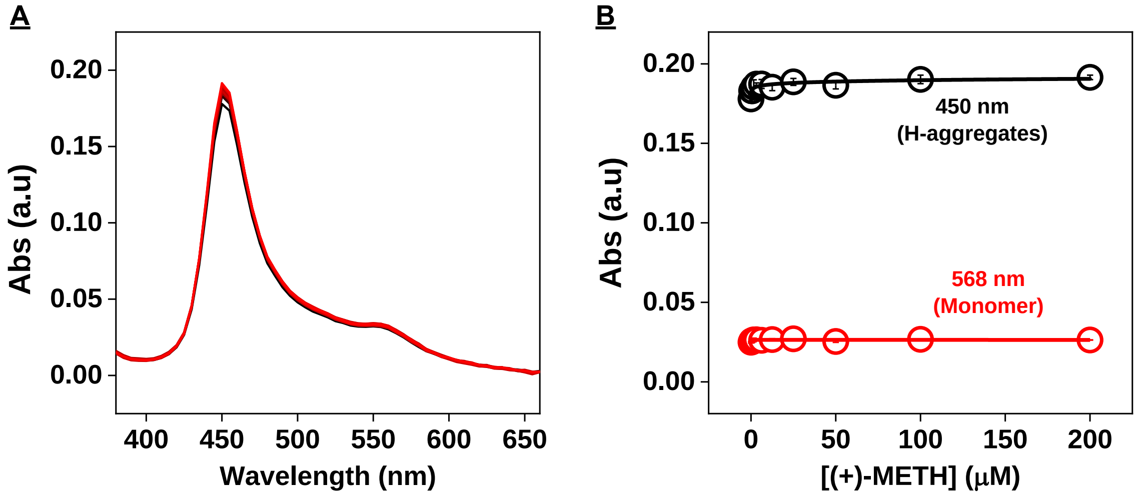

Figure S36. Effect of (+)-methamphetamine on the absorbance spectrum of X-732-91B alone. **(A)** Spectra of 4 μM X-732-91B in the presence of 0, 0.39, 0.78, 1.56, 3.13, 6.25, 12.5, 25, 50, 100, and 200 μM (+)-methamphetamine with no aptamer present, where the black-to-red color gradient represents increasing target concentrations. (**B**) Peak absorbance at 450 (black) and 568 (red) nm as a function of (+)-methamphetamine concentration.

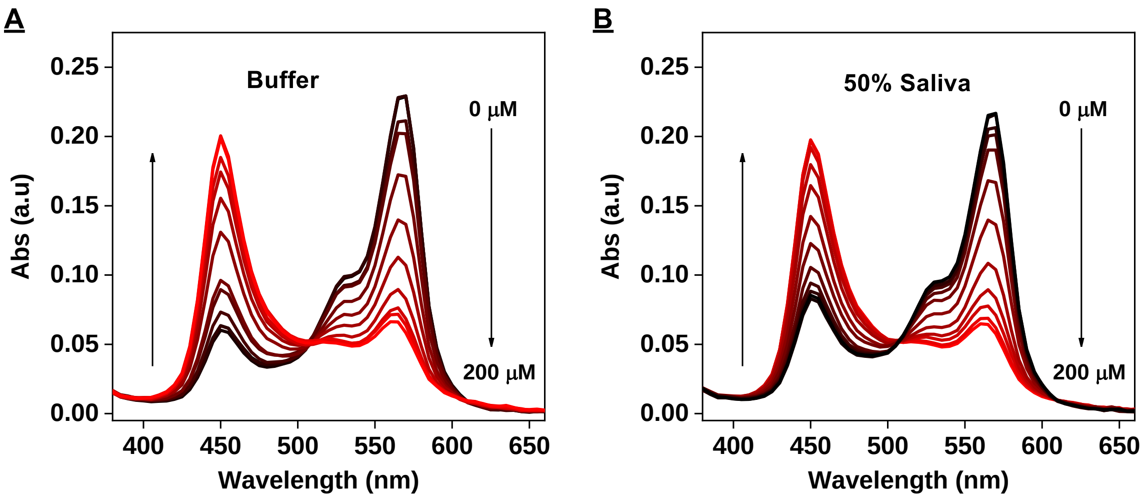

Figure S37. Detection of (+)-methamphetamine using X-732-91B and aptamer ML4. Absorbance spectra in (**A**) buffer and (**B**) 50% saliva for samples containing 4 μM X-732-91B and 6 μM ML4 in the presence of 0, 0.39, 0.78, 1.56, 3.13, 6.25, 12.5, 25, 50, 100, and 200 μM (+)-methamphetamine, where the black-to-red color gradient represents increasing target concentrations.

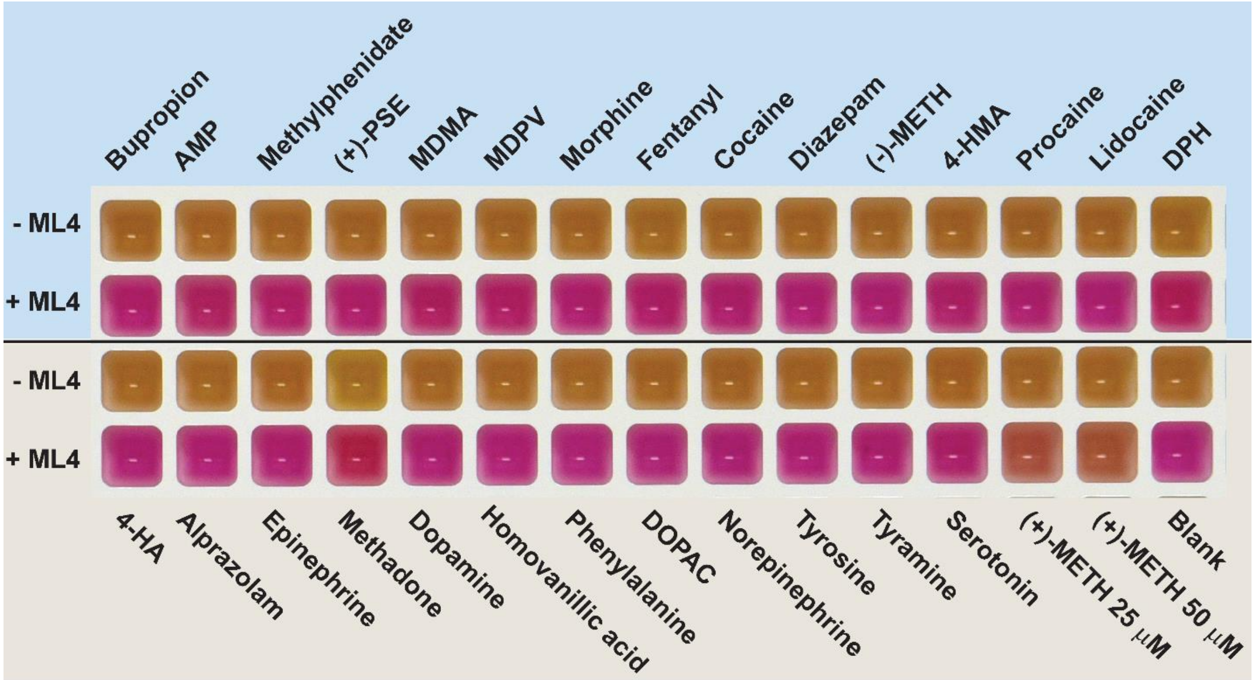

Figure S38. Specificity of a dye-displacement assay for (+)-methamphetamine based on aptamer ML4 and dye X-732-91B**.** Photographs were taken after samples containing dye without aptamer (-ML4) or dye-aptamer complex (+ML4) were challenged with (+)-methamphetamine or various interferents for 10 minutes.

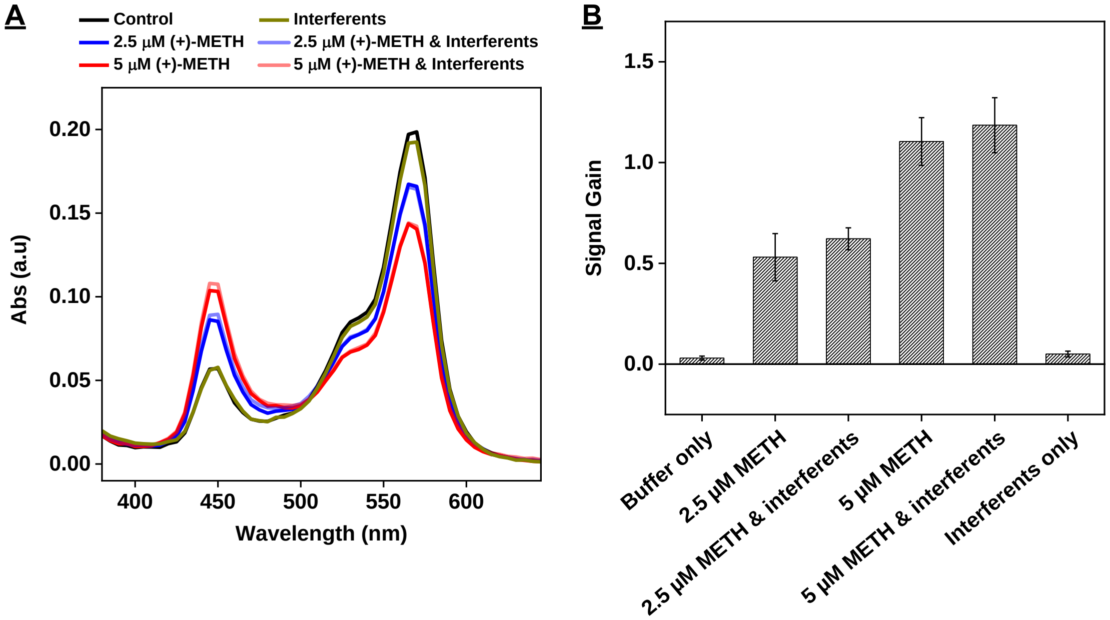

Figure S39. Detection of (+)-methamphetamine in a mixture of interferents using an aptamer-based dye-displacement assay. (**A**) Absorbance spectra in buffer for samples containing 4 μM X-732-91B and 6 μM aptamer ML4 in the presence of 2.5 and 5 μM (+)-methamphetamine alone or with a mixture of 1 μM morphine, 3 μM cocaine, 2 μM methadone, and 1 μM fentanyl. (**B**) Signal gain was calculated as $(R-R_0)/R_0$, where R and R_0 are the ratio of the area under the curve (AUC) for dye aggregates (400–505 nm) relative to the AUC for monomers (505–620 nm) with and without target/interferent, respectively.

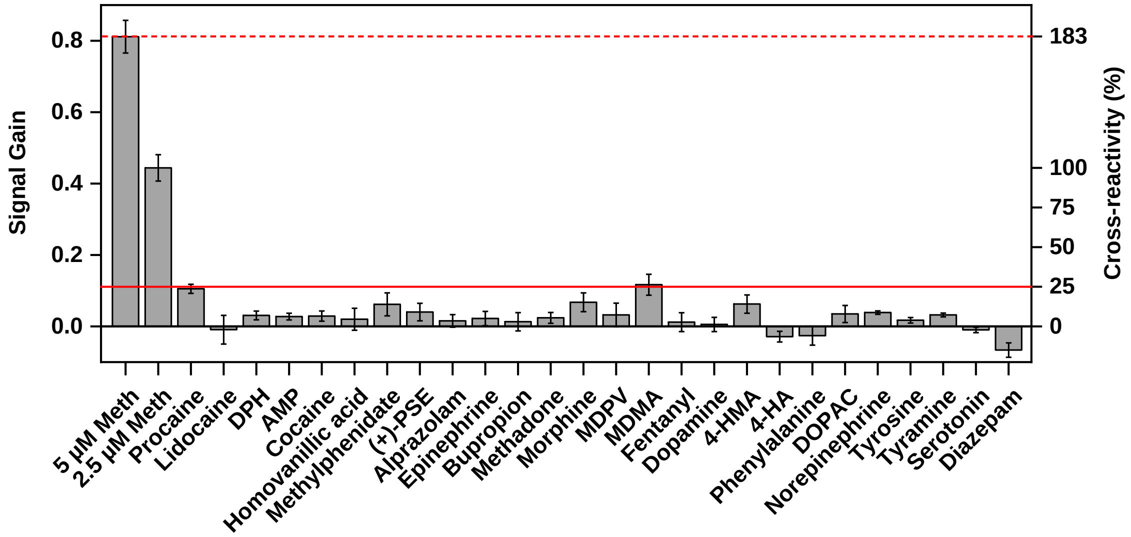

Figure S40. Specificity of a dye-displacement assay for (+)-methamphetamine based on aptamer ML4 and dye X-732-91B. The plot shows signal gain and assay cross-reactivity to clinically relevant target concentrations as well as much higher concentrations of various interferents. The red line demarcates 25% cross-reactivity relative to 2.5 μM (+)-methamphetamine.
